# ETS-1-activated LINC01016 over-expression promotes tumor progression via suppression of RFFL-mediated DHX9 ubiquitination degradation in breast cancers

**DOI:** 10.1038/s41419-023-06016-3

**Published:** 2023-08-08

**Authors:** Ying Sun, Hui Zhang, Ranran Ma, Xiangyu Guo, Guohao Zhang, Sen Liu, Wenjie Zhu, Haiting Liu, Peng Gao

**Affiliations:** 1 Department of Pathology, Qilu Hospital of Shandong University, Jinan, Shandong PR China; 2 Department of Medical Oncology, Qilu Hospital of Shandong University (Qingdao), Qingdao, Shandong PR China

**Keywords:** Breast cancer, Cell growth

## Abstract

Long non-coding RNAs (lncRNAs) are key regulators during the development of breast cancer (BC) and thus may be viable treatment targets. In this study, we found that the expression of the long intergenic non-coding RNA 01016 (LINC01016) was significantly higher in BC tissue samples with positive lymph node metastasis. LINC01016, which is activated by the transcription factor ETS-1, contributes to the overt promotion of cell proliferation activity, enhanced cell migratory ability, S phase cell cycle arrest, and decreased apoptosis rate. By RNA pull-down assays and mass spectrometry analyses, we determined that LINC01016 competitively bound and stabilized DHX9 protein by preventing the E3 ubiquitin ligase RFFL from binding to DHX9, thereby inhibiting DHX9 proteasomal degradation. This ultimately led to an increase in intracellular DHX9 expression and activated PI3K/AKT signaling, with p-AKT, Bcl-2, and MMP-9 involvement. This is the first study to reveal that the LINC01016/DHX9/PI3K/AKT axis plays a critical role in the progression of BC, and thus, LINC01016 may serve as a potential therapeutic target for patients with BC.

## Introduction

Breast cancer (BC) is one of the most common malignant cancers, with 2.3 million newly diagnosed cases and over 685,000 deaths among women in 2020 [1]. Triple-negative breast cancer (TNBC) accounts for 15% of all breast cancers and is associated with poor outcomes compared with other subtypes [2]. Many studies have explored the pathogenic basis of TNBC at the genetic and epigenetic levels [3, 4]. However, the detailed molecular mechanisms governing tumor development and progression remain unclear, and further research works is urgently needed.

Protein-coding genes account for approximately 2% of the human genome, with most intracellular transcripts serving as non-coding RNAs such as long non-coding RNAs (lncRNAs), which are >200 nucleotides in length and participate in a variety of biological processes [5–8]. Abnormal expression of lncRNAs play an important role in carcinogenesis, tumor progression or metastasis [9–11]. Despite these findings, the specific roles of individual lncRNA in BC remain elusive.

Differentially expressed lncRNAs in gastric cancer (GC) samples have been identified by our research team and submitted to the Gene Expression Omnibus (GEO) database (GSE72307). LINC01016 was found to be upregulated in GC tissues from patients with lymph node metastasis (LNM) compared to the GC tissues from patients without LNM. LINC01016 is located on chromosome 6q21.31, and its upregulation has been detected in various tumor types. Yun et al. identified LINC01016 as one of several lncRNAs that were upregulated in thyroid cancer and associated with tumor development [12]. Philip et al. revealed that LINC01016 was upregulated in estrogen receptor (ER) positive subtype breast cancer, which may be related to a poor prognosis [13]. However, its function and potential regulatory mechanism have not been explored. Whether LINC01016 plays a role in the TNBC has not been reported.

This study showed that LINC01016 was upregulated in BC tissue, including the TNBC subtype, and was associated with LNM. Our results highlighted the potential oncogenic role of LINC01016 in human BC. We determined that LINC01016 promoted TNBC cell proliferation and invasion by binding to and stabilizing DHX9 protein through competitive inhibition of the E3 ubiquitin ligase RFFL, thus activated DHX9-mediated PI3K/AKT signaling to accelerate breast tumor progression.

## Materials and methods

### Clinical samples

A total of 101 primary BC samples (including 52 cases with LNM and 49 cases without LNM) were obtained from patients who underwent modified radical mastectomy at the Qilu Hospital of Shandong University (Jinan, Shandong, China) between 2014 and 2018. Our sample size estimation is based on http://powerandsamplesize.com/Website. The Research Ethics Committee of Shandong University oversaw and approved the present study, which conformed with the Declaration of Helsinki. Informed consent has been obtained from all subjects.

### Cell culture and cell transfection

The MDA-MB-231, MDA-MB-468, T47D, MDA-MB-453 human BC cell lines and MCF-10A cell line were purchased from ATCC. Three different Antisense oligonucleotides (ASOs) specific for LINC01016 (si-LINC01016-1, si-LINC01016-2, si-LINC01016-3 and si-LINC01016-4) were obtained from RiboBio (Guangzhou, China). The Turbofect transfection reagent (Thermo Fisher Scientific, USA) was used for plasmid transfection based on provided directions. For in vivo research, a short hairpin RNA (shRNA) construct was used to stably knock down LINC01016 and was expressed with the pGPU6/GFP/Neo vector (GenePharma, Shanghai, China). Sequences for primers and oligonucleotides were listed in Supplementary Table 1.

### Fluorescence in situ hybridization (FISH)

RNA fluorescent in situ hybridization (FISH) assay was performed by using RNA FISH Kit (RiboBio, China). After fixation and permeation, pre-hybridization BC cells were incubated with cy3-labeled probe against LINC01016, U6 snRNA and 18 s rRNA at 37 °C overnight. Washing repeatedly, cells were stained with DAPI. Positive control probes U6 and 18 s (nuclear and cytoplasmic components) were set. DAPI nucleus (blue), U6 nucleus (red), 18 s cytoplasm (red) and LINC01016 (red). Finally, RNA localization were imaged using a fluorescence microscope (Olympus, Tokyo, Japan).

### Subcellular fractionation and quantitative real-time PCR (qRT-PCR)

Cytoplasmic and nuclear RNA fractions were isolated with a PARIS kit (Invitrogen). For quantification of RNA yield, U6 and GAPDH were used as nuclear and cytoplasmic controls, respectively. FastStart Universal SYBR Green Master (Roche Diagnostic GmbH, Mannheim, Germany) was used for qRT-PCR, using GAPDH in data normalization of LINC01016 expression via the 2^−ΔCT^ method.

### Cell proliferation, wound healing and Transwell® assays

Colony formation assay, MTS and EDU proliferation assay, wound healing and Transwell assays were carried out as previously described [14].

### Apoptosis and cell cycle progression analysis

Cell progression was assessed via propidium iodide (PI; Beyotime, Shanghai, China) staining for 30 min at 4 °C. Apoptotic cell death was assessed with a FITC-Annexin V Apoptosis Detection kit (Beyotime, Shanghai, China). Cells were then analyzed with a FACS can flow cytometer (BD Biosciences).

### Western blotting

Western blotting was conducted as in prior studies using antibodies from Abcam (anti-ETS-1 [ab307672], -DHX9 [Ab26271]), Proteintech (anti-RFFL [Cat No. 12687-1-AP], -HA [Cat No. 81290-1-RR], -bcl-2 [Cat No. 12789-1-AP], -MMP-9 [Cat No. 10375-2-AP], -p-AKT (Ser 473) [Cat No. 80455-1-RR], and -AKT [Cat No. 60203-2-Ig]), and Bioss (anti-tubulin) [15]. Antibodies were diluted within the 1:800–1:2000 range, using GAPDH as a loading control.

### Immunohistochemistry

A streptavidin-peroxidase (S-P) approach was used to stain sections. Monoclonal rabbit anti-ki67 (1:500, Proteintech) was used to stain cells overnight at 4 °C. Staining intensity was scored (0 = negative, 1 = weak, 2 = moderate, and 3 = strong), and the percentage of positively stained cells was determined (0 = 0%, 1 = 1%–25%, 2 = 26%–50%, 3 = 51%–75%, and 4 = 76%–100%). The IHC score was calculated using the equation: IHC score = P1 × 1 + P2 × 2 + P3 × 3 (P: percentage). These scores were added to produce the final score: high (score ≥ 4) and low or none (score = 0–3).

### Co-immunoprecipitation (Co-IP) assay

Whole-cell lysates containing 200–400 μg total protein were combined with 1–2 μg primary antibodies (anti-DHX9 [Abcam, Ab26271], anti-RFFL [Proteintech, 12687-1-AP], or anti-FLAG/DYKDDDDK Tag [Cell Signaling Technology, 14793]) overnight, after which 20 μL of protein A/G agarose beads (P2012, Beyotime) was added to each sample and incubated for 1 h at 4 °C. Samples were then centrifuged to collect immune complexes, which were analyzed via western blotting or mass spectrometry (MS) as appropriate.

### In vitro ubiquitination assay

BC cell lines were co-transfected with LINC01016, HA, and DHX9 plasmids. Following a 48-h incubation, anti-DHX9 was used to precipitate prepared cell lysates at 4 °C overnight, after which precipitates were assessed by western blotting.

### Vector construction

Portions of the LINC01016 promoter (−1,000/0, −750/0, −500/0, −250/0, and −125/0) were PCR amplified from MDA-MB-231 cell genomic DNA. These sequences were then inserted into the pGL3-Basic vector (Promega) upstream of firefly luciferase using the *Kpn*I-*Xho*I sites. Construct naming was based on the promoter fragment location relative to the transcription start site (TSS). Overexpression plasmids for ETS-1, DHX9, CEBP-β, and MEIS-1 were obtained from Vigene Biosciences (Rockville, MD, USA). Overexpression plasmids for ELK1, FOS, JUN, SP1, and E2F-1 were produced in our laboratory. The ubiquitin HA plasmid was obtained from Dr. Li (Shandong University). Sequencing was used to confirm vector integrity using primers shown in Supplementary Table 1.

### Luciferase assay

MDA-MB-231 and MDA-MB-468 cells were seeded into 24-well plates and co-transfected with a transcription factor construct (0.5 μg), LINC01016 promoter-luciferase reporter plasmid (0.5 μg), and pRL-TK plasmid (0.01 μg). After 48 h, a dual-luciferase reporter assay (Promega) was used following the manufacturer’s instructions. Firefly luciferase activity was normalized to *Renilla* luciferase activity.

### Chromatin immunoprecipitation (ChIP) and RNA immunoprecipitation (RIP)

Chromatin immunoprecipitation (ChIP) and RNA immunoprecipitation (RIP) assays were performed as previously described [16, 17]. Eluted DNA or RNA was purified and assessed via qRT-PCR. The primer sequences are shown in Supplementary Table 1.

### RNA pull-down assay and mass spectrometry

A MEGAscript T7 Transcription Kit (Ambion, CA, USA) was used to in vitro transcribe and biotin label LINC01016 and its antisense RNA, followed by purification with a MEGAclear Kit (Ambion). These RNA oligomers (3 μg) were then combined with 1 mg of protein lysate and 60 μL of streptavidin beads (Invitrogen) for 1 h with constant agitation. RIP buffer was then used to wash the samples five times and then boiled in sodium dodecyl sulfate (SDS). The associated proteins were resolved by SDS-polyacrylamide gel electrophoresis, and the differential bands were analyzed by MS. The candidate proteins were finally confirmed by western blotting. The proteins were selected according to the following criteria: 1) at least two-fold score difference in the sense LncRNA group compared with the antisense LncRNA group; 2) molecular weight between 130 kDa and 170 kDa; 3) mainly located in the nucleus of cells; and 4) oncogenic function.

### Tumor xenograft model

The right dorsal flank or the lateral tail vein of nude athymic mice (female; 4 weeks old) from Weitonglihua Biotechnology (Beijing, China) was implanted with MDA-MB-231 cells (LV-NC or LV-siLINC01016) (10^7^ or 10^5^ cells, respectively). The nude mice were randomly assigned to the experimental group and the control group by simple randomization method. Each group had 6 nude mice. Tumor volume (V) was monitored weekly based on the formula V = (Tumor length × Width^2^)/2. At 8 weeks post-implantation, animals were euthanized, and lungs, livers, and tumor tissues were collected. Because the tumor cells contain GFP, the site and number of metastases can be observed using an animal imaging system. For animal trials, we estimated the sample size by obtaining relevant information from pre-experiments or published articles, using a double-blind method for analysis.

### Statistical analysis

SPSS 22.0 and GraphPad Prism (v5.0, USA) were used for statistical analysis. The significance of the differences was confirmed using Student’s *t* test between two groups or with one-way ANOVA for the three groups. Chi-square and Fisher’s exact tests were used to analyze the association between LINC01016 expression and clinicopathological characteristics. The data were presented as the mean ± standard deviation (SD) of three independent experiments. Differences with a two-sided *P* ≤ 0.05 were considered statistically significant.

## Results

### LINC01016 is upregulated in BC tissue with LNM and is associated with poor prognosis in patients with BC

**Fig. 1:**
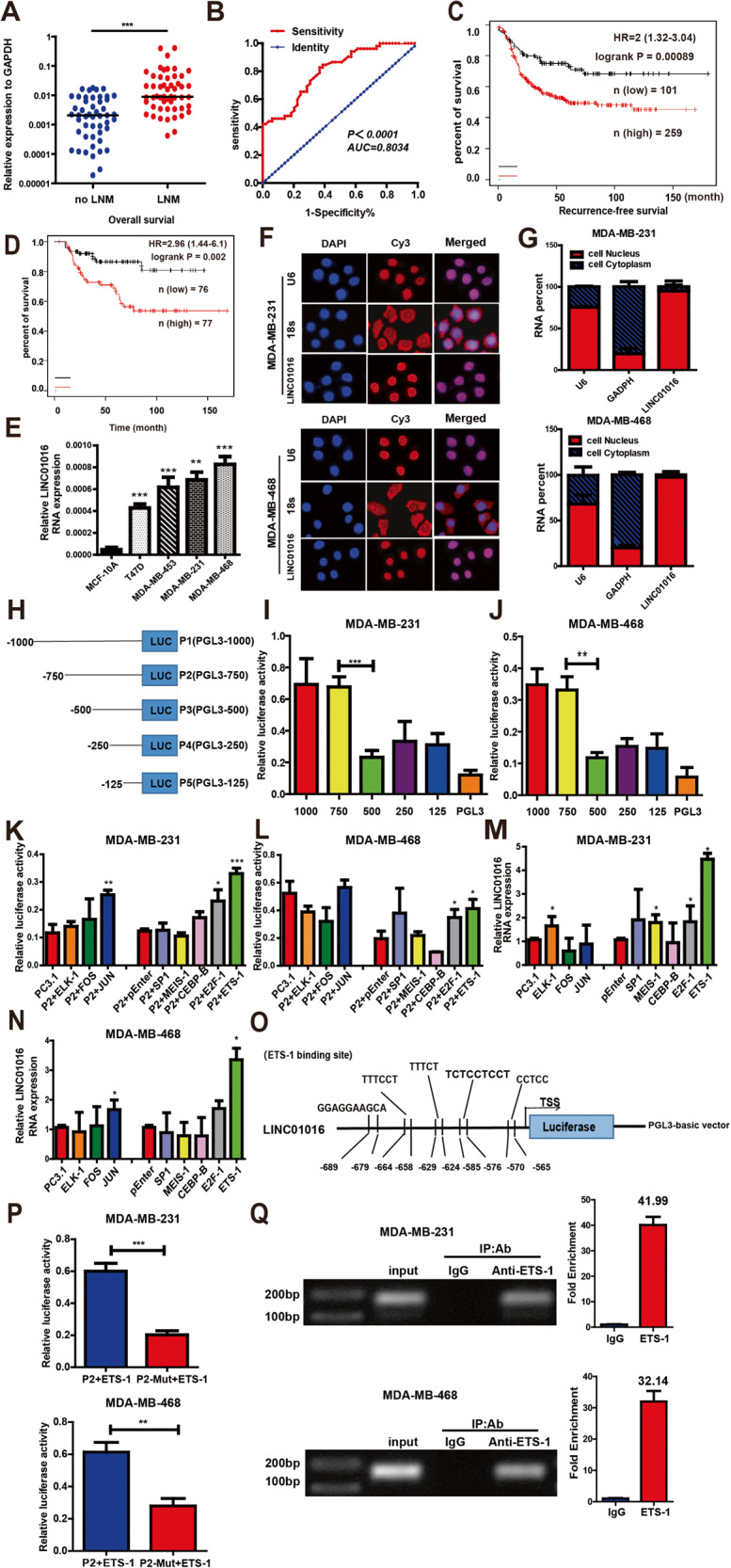
LINC01016 is upregulated in BC tissues exhibiting LNM and is associated with BC progression. **A** The expression of LINC01016 is compared in BC tissues with LNM (n = 52) and without LNM (n = 49). **B** ROC curve is used to identify that LINC01016 could be used to distinguish the patients with LNM. **C**, **D** In the basal-like subtype of BC, patients with higher LINC01016 expression have worse recurrence-free survival (RFS) and overall survival (OS) rates, according to the analysis results of TCGA database. Higher than median LINC01016 expression was defined as high expression. On the contrary, the definition is low expression. **E** Compared with the human mammary epithelial cell line MCF-10A, the relative expression of LINC01016 in TNBC cell lines MDA-MB-231 and MDA-MB-468 was significantly upregulated. **F** RNA FISH assays reveals that LINC01016 is primarily located in the nucleus. U6 and 18 S are utilized as controls for these localization analyses. **G** LINC01016, U6, and GAPDH levels are assessed in nuclear and cytoplasmic BC cell fractions by qRT-PCR. **H** Fragments of the LINC01016 promoter are cloned into the pGL3-basic vector upstream of firefly luciferase. (pGL3-1000, pGL3-750, pGL3-500, pGL3-250, pGL3-125) **I**, **J** Luciferase activity assays disclose that the promoter activity is significantly decreased from pGL3-750 to pGL3-500. **K**, **L** ETS-1 and E2F-1 overexpression, respectively, lead to a significant increase in luciferase activity. **M**, **N** ETS-1 overexpression promote the expression of LINC01016 via qRT-PCR in BC cells. **O** The JASPAR website identifies the potential ETS-1 binding sites in the LINC01016 promoter (from −689 to −565 bp). **P** LINC01016 binding site mutants exhibite reduced luciferase activity. **Q** ETS-1 enrichment at the LINC01016 promoter is detected via ChIP assay. Data are presented as means ± SEM, from three independent experiments. *P < 0.05, **P < 0.01,***P < 0.001.

In this study, we extended the results of RNA-seq analysis of previous GC tissue samples to BC. We found that the expression of LINC01016 was nine-fold higher in BC patients with LNM relative to that in patients without LNM. Similar results were seen in TNBC, in which LINC01016 expression was 8.3-fold higher in patients with LNM than in those without LNM (Fig. 1A, Supplementary Fig. 1A). Additionally, we found the diagnostic value of LINC01016 to distinguish patients with or without LNM by conducting Receiver Operating Characteristic (ROC) curve analysis, with the area under the curve reaching up to 0.8034 (Fig. 1B).

The relationships between LINC01016 and clinicopathological features were also analyzed. High LINC01016 expression was found to be associated with higher Ki67 index and positive LNM (Table 1). In the TNBC subtype, recurrence-free survival (RFS) and overall survival (OS) rates were significantly lower in patients with higher LINC01016 levels, according to an analysis of data from the TCGA database (Fig. 1C, D).

**Table 1:** Correlation between LINC01016 expression and patients’ clinicopathological characteristics.

Character		LINC01016 Expression		
	Number	High	Low	*P*
Age				
<60	55	29	26	0.1040
≥60	46	32	14	
Histological grade				
I–II	49	27	22	0.3153
III	52	34	18	
ER				
Negative	51	35	16	0.1057
Positive	50	26	24	
PR				
Negative	53	28	25	0.1096
Positive	48	33	15	
Her-2				
Negative	46	29	17	0.6852
Positive	55	32	23	
Ki-67				
<15	45	19	26	**0.0011**
≥15	56	42	14	
Tumer size(cm)				
≤2 cm	40	21	19	0.2161
Å 2 cm	61	40	21	
Lymphatic metastasis				
Negative	49	18	31	**<0.0001**
Positive	52	43	9	

Compared with a non-tumorigenic human breast epithelial cell line (MCF-10A), we found that the expression of LINC01016 in BC cell lines was significantly upregulated, especially in TNBC (Fig. 1E). The intracellular localization of LINC01016 was assessed via FISH and qRT-PCR. We determined that LINC01016 was mainly enriched in the nuclei of BC cells (Fig. 1F, G), suggesting that it may have nucleoprotein binding activities as a means of regulating gene transcription.

### ETS-1 activates LINC01016 transcription and promotes BC progression

Based on the human LINC01016 promoter sequence from the UCSC Genome Browser (http://genome.ucsc.edu/), five regions upstream of the TSS were constructed into the pGL3-basic vector to identify the core promoter region using a luciferase activity assay (Fig. 1H). The results showed that promoter activity significantly decreased from pGL3-750 to pGL3-500, suggesting that the upstream region from −750 bp to −500 bp was required for LINC01016 transcription (Fig. 1I, J).

We next utilized the JASPAR program (http://jaspar.genereg.net/downloads/) to identify the putative transcription factors (TFs) binding to the core promoter region. Within the −750 bp to −500 bp region, putative TFs with high scores included ELK-1, FOS, JUN, SP1, MEIS-1, CEBP-β, E2F-1, and ETS-1. Using a luciferase assay, we found that overexpression of ETS-1 and E2F-1 in both BC cell lines significantly enhanced the promoter activities of pGL3-750 (Fig. 1K, L). However, only ETS-1 remarkably increased LINC01016 expression, as indicated by a qRT-PCR assay (Fig. 1M, N). Potential ETS-1 binding sites were identified via the JASPAR program, primarily between −689 bp and −565 bp (Fig. 1O). To further confirm the direct interaction between ETS-1 and the LINC01016 promoter, we generated a deletion mutant construct (P2-Mut). ETS-1-overexpressing cells with P2-Mut showed a 50–70% reduction in luciferase activity relative to P2 wild-type (Fig. 1P). Next, ETS-1 was confirmed to directly bind to the LINC01016 promoter using a ChIP assay (Fig. 1Q). Together, these results suggested that ETS-1 activated LINC01016 transcription.

**Fig. 2:**
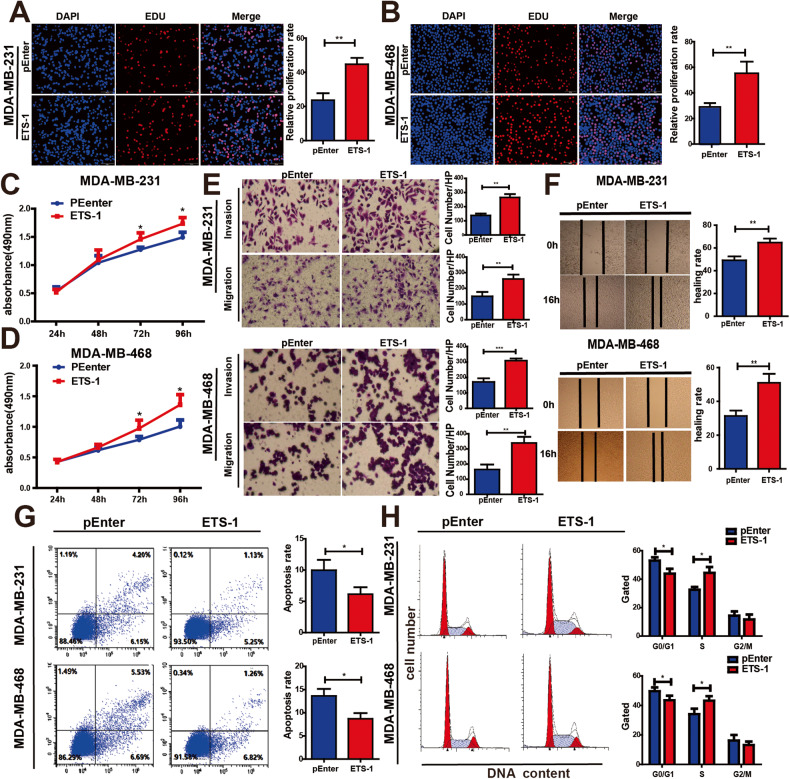
ETS-1 accelerates BC cell malignant progression. **A**, **B** EdU assay reveals that ETS-1 overexpression significantly enhances BC cell proliferation. **C**, **D** MTS assay shows that ETS-1 upregulation remarkably accelerates BC cell proliferation. **E** Transwell assays show that overexpression of ETS-1 enhances the ability of cell migration and invasion. **F** Wound healing assays confirm that ETS-1 overexpression enhances cell migration. **G** Flow cytometry reveals that ETS-1 overexpression reduces the rate of BC cell apoptosis. **H** Overexpression of ETS-1 promotes cell cycle progression from the G0/G1 phase to the S phase in BC cells. Data are represent as the mean ± SEM. *P < 0.05, **P < 0.01,***P < 0.001.

A subsequent EdU assay and MTS assay revealed that ETS-1 significantly enhanced BC cell proliferation (Fig. 2A–D). Similarly, ETS-1 enhanced BC cell migration and invasion in Transwell assays and wound healing (Fig. 2E, F). ETS-1 overexpression reduced the apoptotic rate of BC cells and promoted cell cycle progression into the S phase (Fig. 2G, H). These results suggest that ETS-1 activated LINC01016 transcription and promoted BC progression.

### LINC01016 enhances the proliferative and invasive activities of BC cells

We next modulated the expression levels of LINC01016 in two BC cell lines (MDA-MB-231 and MDA-MB-468) using ASO and PCDNA3.1-LINC01016 vector constructs to achieve lncRNA knockdown and overexpression, respectively, with the efficiency confirmed via qRT-PCR (Supplementary Fig. 1B, C and Supplementary Fig. 3A). Of the four tested ASO constructs, si-LINC01016-3 and si-LINC01016-4 successfully reduced LINC01016 expression by > 50% and thus was used in the subsequent functional studies (Supplementary Fig. 1C and Supplementary Fig. 3A).

**Fig. 3:**
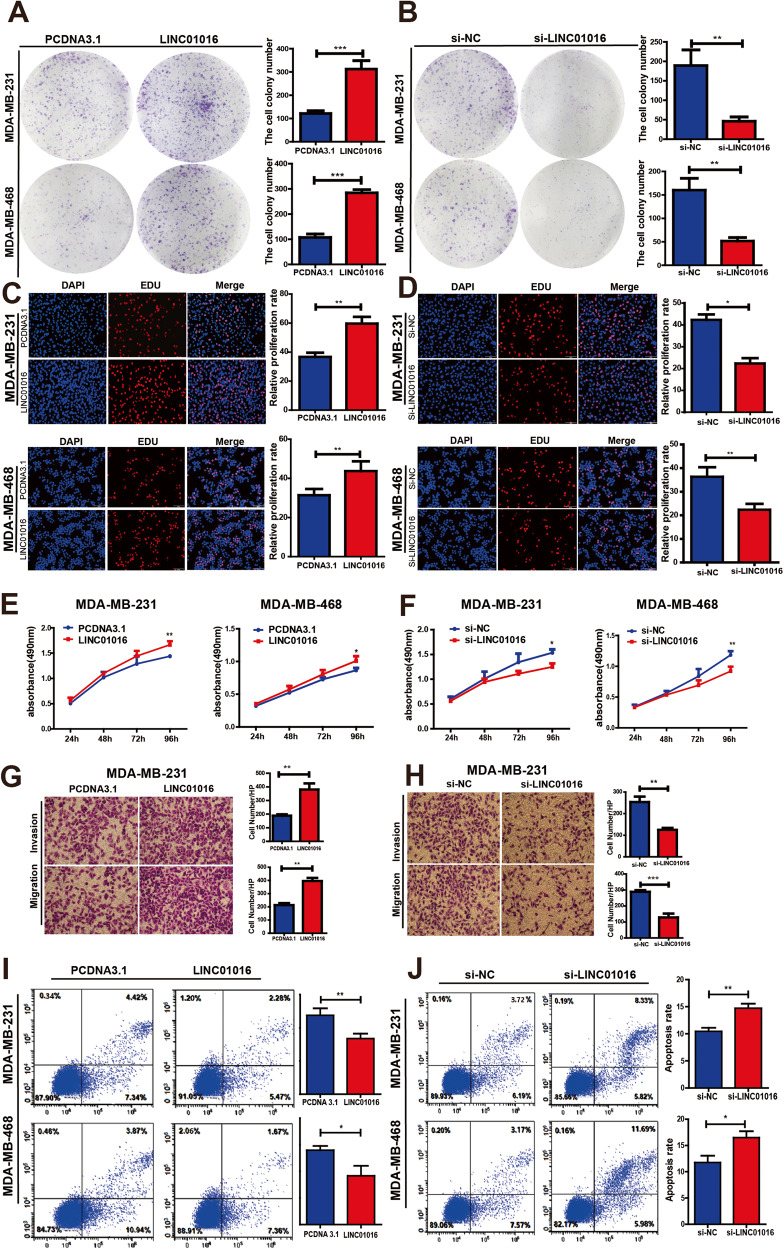
LINC01016 is is associated with BC progression. **A**, **B** The results of clone formation experiment discover that overexpression of LINC01016 promotes cell proliferation, while konckdown of LINC01016 inhibits cell proliferation. **C**, **D** The results of EdU assays indicate that the up/downregulation of LINC01016 could enhance and repress the proliferation of BC cells. **E**, **F** The results of MTS assays show that up/downregulation of LINC01016 accelerates and inhibits the proliferation of BC cells. **G**, **H** The results of transwell assays show that LINC01016 overexpression enhances the migration and invasion of BC cells, while silencing suppresses both activities. **I**, **J** Apoptotic rates are decreased when LINC01016 is overexpressed and are increased when it is knockdown. Data are shown as mean ± SEM of three independent experiments, *P < 0.05, **P < 0.01, ***P < 0.001, ns not significant.

LINC01016 overexpression enhanced the size and number of BC cell colonies in a colony formation assay (Fig. 3A), whereas knockdown of LINC01016 had the opposite effect (Fig. 3B). EdU incorporation assays further suggested that LINC01016 overexpression enhanced proliferation, whereas LINC01016 knockdown dramatically inhibited the growth capabilities of BC cells (Fig. 3C, D and Supplementary Fig. 3B). Consistent results were also obtained with an MTS assay, thus supporting the proliferation-enhancing role of LINC01016 in BC (Fig. 3E, F and Supplementary Fig. 3C).

Given that we found LINC01016 levels were correlated with the LNM status of BC patients, we next explored the relationship between LINC01016 and the migratory or invasive capabilities of BC cells. Transwell® assays confirmed that LINC01016 overexpression enhanced the migration and invasion of BC cells, whereas silencing its expression suppressed both of these activities (Fig. 3G, H; Supplementary Fig. 1E; Supplementary Fig. 3D). Together, these findings suggested that LINC01016 plays key roles in enhancing the proliferation, invasion, and metastasis of BC cells.

### LINC01016 promotes BC cell survival and cell cycle progression

Next, we examined the impact of LINC01016 on apoptosis and cell cycle progression. We found that the apoptotic rates of BC cells decreased when LINC01016 was overexpressed and increased when it was knocked down (Fig. 3I, J and Supplementary Fig. 3E). In line with these findings, the upregulation of LINC01016 resulted in an increase in the number of G0/G1 cells entering the S phase of the cell cycle, whereas LINC01016 knockdown was associated with G0/G1 phase arrest (Supplementary Fig. 1F). Together, these results indicated that LINC01016 promoted tumor growth by increasing the proliferation and survival of tumor cells.

### LINC01016 physically binds with DHX9 to promote BC progression

**Fig. 4:**
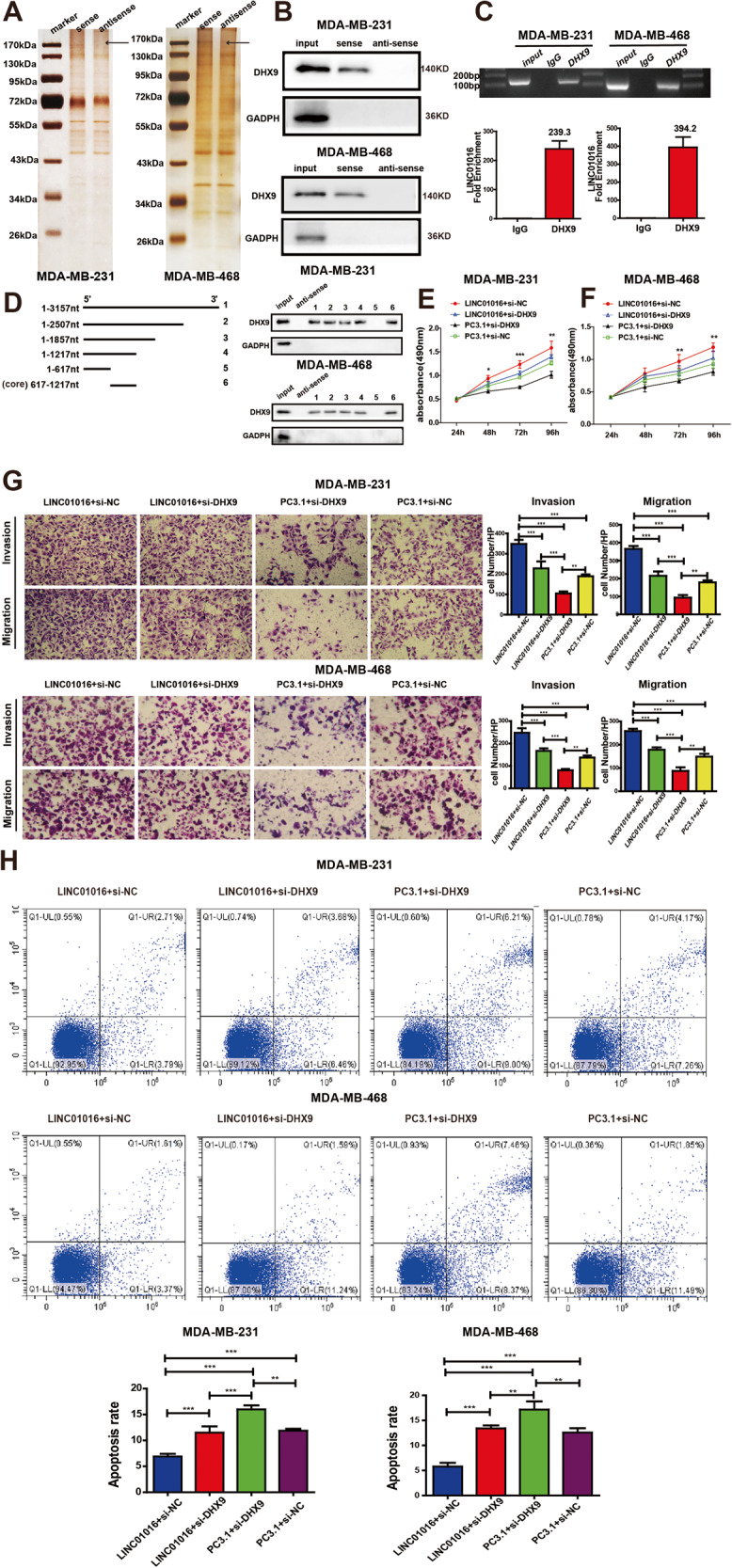
DHX9 is a binding partner of LINC01016. LINC01016 and DHX9 work together to promote BC cell progression. **A** A number of proteins with molecular weight between 130 kDa and 170 kDa are found to bind to LINC01016 via pull-down assay and silver staining. The band specific to LINC01016 is subjected to mass spectrometry. **B** DHX9 is ultimately identified as a LINC01016-interacting protein by western blotting. **C** The binding of LINC01016 and DHX9 is confirmed via RIP assay, with qRT-PCR products being verified via agarose electrophoresis. **D** Truncated biotin-linked LINC01016 are used to pull down cellular protein and reveal that 617-1217 nt core sequence of LINC01016 binds to DHX9. **E**, **F** DHX9 knockdown is found to be able to reduce LINC01016-mediated enhancement of BC cell proliferation. **G** DHX9 knockdown reverses the impact of LINC01016 overexpression on BC cell migration and invasion. **H** LINC01016-induced apoptotic resistance is suppressed by DHX9 knockdown. Data are represent as the mean ± SEM from triplicate experiments. *P < 0.05, **P < 0.01,***P < 0.001.

Given that LncRNAs often affect cellular function through competing endogenous RNA (ceRNA) or binding proteins and previous studies have reported that LINC01016 could affect cellular function through ceRNA mechanisms, could it contribute through nucleobinding proteins? An RNA pull-down assay was conducted to identify proteins that bind to LINC01016. A number of proteins with molecular weights between 130 kDa and 170 kDa were found to bind to LINC01016 and not the LINC01016 antisense transcript (Fig. 4A). According to the MS results, two molecules were screened as candidate proteins, DHX9 and EPRS (Supplementary Table 2). DHX9 was ultimately identified as a LINC01016-interacting protein by western blotting (Fig. 4B). This result was independently confirmed via an RIP assay (Fig. 4C). When we further conducted RNA pull-down analyses with different LINC01016 segment probes, we found that only the probe containing the 617–1,217 nucleotide (nt) segment was able to pull down DHX9, suggesting that it was the core region for binding between LINC01016 and DHX9 (Fig. 4D).

Prior to assessing the relationship between DHX9 and LINC01016 during BC progression, the oncogenic role of DHX9 was explored. Overexpression of DHX9 significantly enhanced BC cell proliferation in an MTS assay, and its knockdown had the opposite effect (Supplementary Fig. 1G). Consistent with these findings, BC cell apoptosis was markedly reduced when DHX9 was upregulated and increased when it was knocked down (Supplementary Fig. 1H). DHX9 overexpression further enhanced the migratory and invasive activities of BC cells, and DHX9 silencing produced the opposite phenotype (Supplementary Fig. 1I, J). In EdU and cell cycle assays, however, modulation of DHX9 expression levels did not alter BC cell activity (Supplementary Fig. 2A–D). To determine whether DHX9 served as a mediator of LINC01016-induced BC cell proliferation and metastasis, we next knocked down DHX9 in cells overexpressing LINC01016. This approach revealed that DHX9 knockdown was sufficient to reverse LINC01016-dependent promotion of cell proliferation (Fig. 4E, F), migratory and invasive activities (Fig. 4G), and apoptosis resistance (Fig. 4H). These results suggested that LINC01016 promoted BC progression at least in part by binding to DHX9.

### LINC01016 competitively binds with DHX9 and suppresses RFFL-mediated DHX9 ubiquitination and degradation

**Fig. 5:**
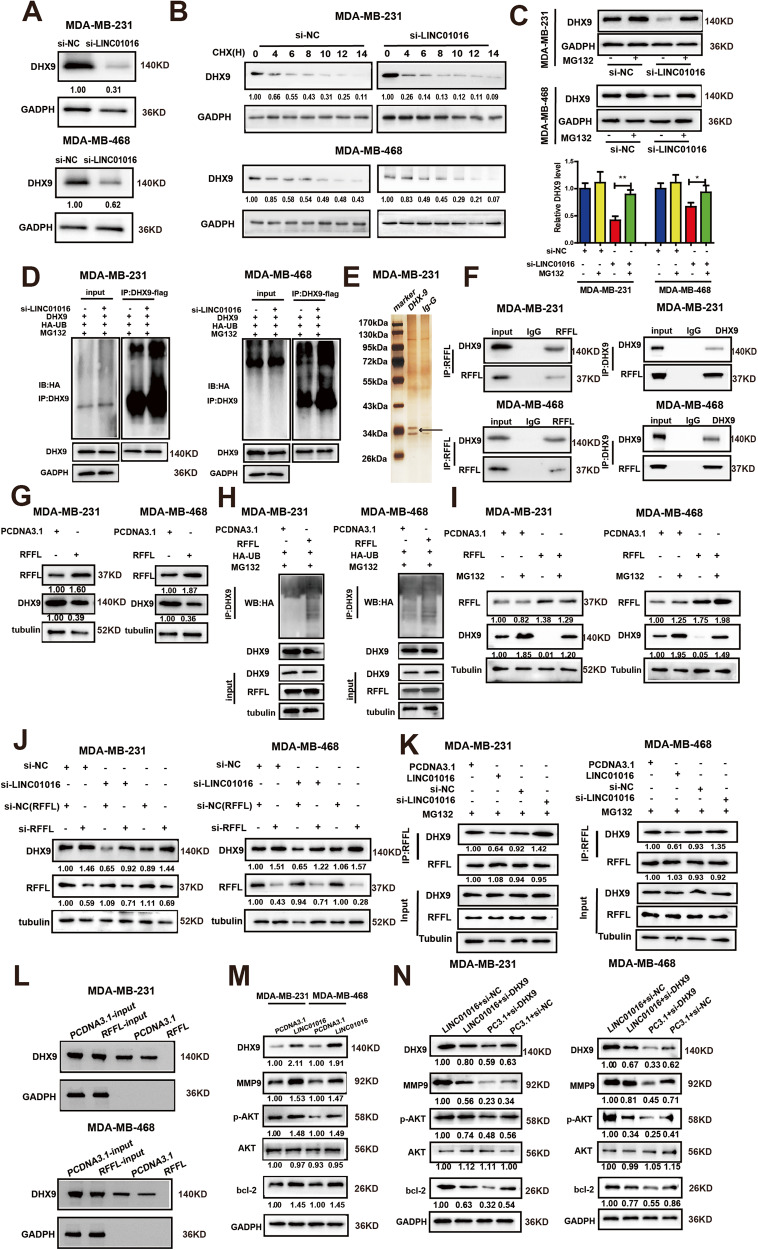
LINC01016 promotes triple-negative breast tumor proliferation and metastasis via suppression of RFFL-mediated DHX9 ubiquitination degradation to activate the PI3K/AKT pathway. **A** Knockdown of LINC01016 reduced DHX9 protein levels. **B** si-LINC01016 promotes more rapid DHX9 protein degradation, as demonstrated by western blotting following CHX treatment (20 μg/mL for 0–14 h). **C** Western blotting results show that intracellular DHX9 level is decreased in si-LINC01016-transfected cells that have not been treated with MG132 (10 μM) for 24 h. **D** si-NC- and si-LINC01016-transduced cells are transfected with HA-Ub and DHX9 plasmids and cultivated for 48 h, after which they are treated for 4 h with MG132 (10 μM). A DHX9-specific antibody is then used for immunoprecipitation of these cell lysates, revealing increased DHX9 ubiquitination in cells transduced with si-LINC01016. **E** RFFL is identified as an E3 ubiquitin ligase of DHX9 via Co-IP and MS assays, with the different band being marked with an arrow. **F** The results of Co-IP and subsequent western blotting using anti-DHX9, anti-RFFL, or control IgG reveal the interactions between DHX9 and RFFL. **G** RFFL is found by western blotting to negatively regulate DHX9 expression. **H** Cells overexpressing RFFL or control constructs were transfected with the HA-UB plasmid and cultivated for 48 h and then treated for 4 h with MG132 (10 μM). Increased DHX9 ubiquitination was observed in cells overexpressing RFFL relative to control cells. **I** Western blotting shows that RFFL overexpression is associated with reduced DHX9 protein levels, with MG132 treatment disrupting this association. **J** DHX9 levels were found to be reduced in cells in which LINC01016 had been knocked down, whereas RFFL knockdown reversed this effect. **K** Immunoprecipation assay showing that LINC01016 overexpression disrupts RFFL binding to DHX9, while LINC01016 knockdown had the opposite effect. **L** RFFL overexpression disrupts interactions between DHX9 and LINC01016, as confirmed via an RNA pull down assay wherein lysates of cells overexpressing RFFL or control constructs were combined with in vitro-transcribed biotinylated LINC01016 sense transcripts, followed by the assessment of DHX9 via western blotting. **M** PI3K/AKT pathway signaling-related proteins (p-AKT, bcl-2 and MMP-9) were upregulated in cells overexpressing LINC01016. **N** LINC01016 upregulation activated the PI3K/AKT pathway signaling activity, whereas DHX9 knockdown reversed this effect. Data are shown as mean ± SEM of three independent experiments, *P < 0.05, **P < 0.01, ***P < 0.001, ns. not significant.

There is mounting evidence showing that lncRNAs can target RNA-binding proteins (RBPs) at the transcriptional or post-transcriptional levels [18–20]. Then, we observed the effect on the mRNA level and protein of DHX9 after knocking down LINC01016. We treated BC cells with actinomycin D (ActD) and assessed DHX9 mRNA stability. Knockdown LINC01016 had no effect on the DHX9 mRNA expression levels (Supplementary Fig. 2E, F). However, knockdown of LINC01016 reduced the expression of DHX9 protein levels (Fig. 5A). Treatment of cells with cycloheximide (CHX) to inhibit protein synthesis demonstrated that LINC01016 silencing reduced DHX9 protein stability (Fig. 5B). This effect, however, was reversed when cells were treated with the proteasome inhibitor MG132 (Fig. 5C). Moreover, we also revealed that there was an increased level of DHX9 ubiquitination in LINC01016 silencing in BC cells compared to the vector controls (Fig. 5D). Together, these findings strongly suggested that LINC01016-silenced BC cells was sufficient to reduce DHX9 protein stability by promoting its ubiquitination and proteasomal degradation.

We next conducted a Co-IP assay to identify the E3 ubiquitin ligase responsible for DHX9 ubiquitination (Fig. 5E). The proteins provided by the MS analysis were screened using the following criteria: 1) the anti-DHX9 antibody group showed at least a two-fold score difference compared to the anti-IgG antibody group; 2) the molecular weight was between 30 kDa and 40 kDa; and 3) the protein was an E3 ubiquitin ligase. RING finger and FYVE-like domain-containing protein 1 (RFFL) was eventually identified (Supplementary Table 3). We further observed an endogenous interaction between DHX9 and RFFL in BC cells via a Co-IP assay (Fig. 5F). RFFL upregulation led to a significant reduction in DHX9 protein expression (Fig. 5G), suggesting that RFFL acted as a negative regulator of DHX9 expression. In line with this model, we found that overexpressing RFFL increased DHX9 ubiquitination (Fig. 5H), and MG132 treatment was sufficient to reverse DHX9 degradation in RFFL-overexpressing cells (Fig. 5I). LINC01016 knockdown resulted in a remarkable reduction in DHX9 expression, whereas knockdown of RFFL in these cells reversed this effect (Fig. 5J). These findings strongly suggest that DHX9 underwent RFFL-mediated ubiquitination and subsequent degradation.

Furthermore, Co-IP experiments showed that the binding ability of RFFL to DHX9 decreased when LINC01016 was overexpressed, while this ability increased when LINC01016 was knocked down (Fig. 5K). To assess whether RFFL overexpression impacted the interaction between LINC01016 and DHX9, we obtained cell lysates treated with MG132 from RFFL-overexpressing or control cells and used these in a biotinylated LINC01016 pull-down assay. The results revealed that the ability of LINC01016 to bind to DHX9 significantly decreased when RFFL was overexpressed (Fig. 5L). Together, these results suggested that LINC01016 competitively bound to DHX9, thereby reducing RFFL-mediated ubiquitination and subsequent degradation, leading to the enhancement of BC cell malignancy.

### LINC01016 induces DHX9-dependent PI3K /AKT signaling in BC cells

As LINC01016 was able to increase intracellular DHX9 levels, we explored the signaling mechanisms that contributed to BC proliferation and metastasis. We analyzed mRNA levels after DHX9 overexpression in MDA-MB-231 cells, and the PI3K/AKT signaling pathway was found to be significantly upregulated (Supplementary Table 4). We speculated that it may be involved in a regulatory mechanism of DHX9. AKT is a key molecule in the PI3K/AKT signaling pathway, and western blotting analysis revealed that LINC01016 overexpression increased the expression of p-Akt, MMP-9, and Bcl-2 (Fig. 5M), while these increases were reversed when cells were treated with si-DHX9 (Fig. 5N). These findings suggested that LINC01016 promoted tumor progression through a DHX9-dependent mechanism that promoted PI3K/AKT signaling in BC cells.

### LINC01016 may be a potential therapeutic target for BC

**Fig. 6:**
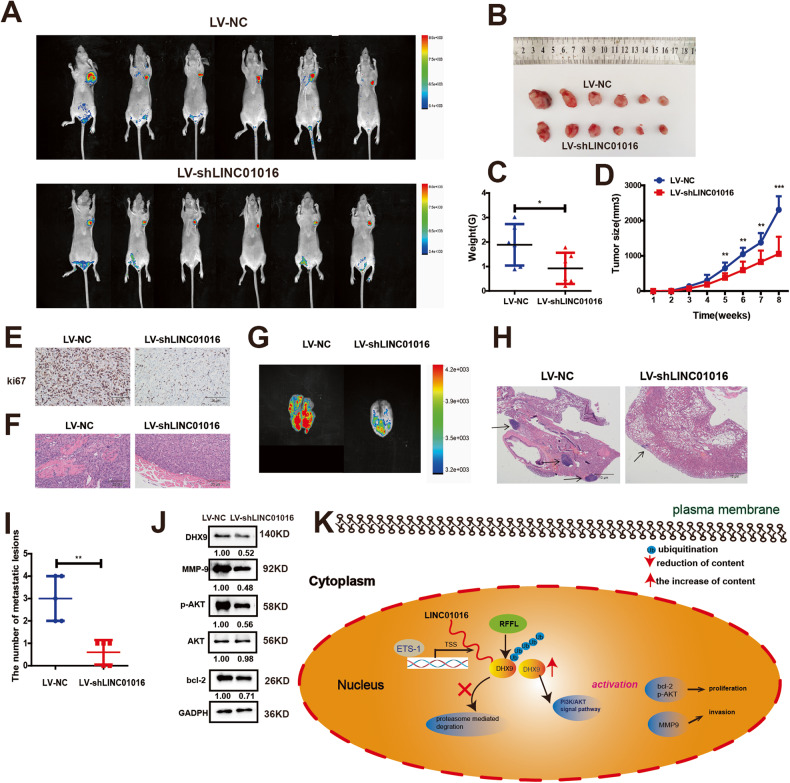
LINC01016 may be a potential therapeutic target for BC. **A**, **B** Eight weeks later, the mice were sacrificed and tumors were collected and imaged. The tumor size of the LV-shLINC01016 group was smaller than that of the LV-NC group. **C** Tumor weights. **D** Tumor growth was monitored over time following implantation, which revealed slower tumor growth rates in mice implanted with LV-shLINC01016-transduced tumor cells relative to LV-NC controls. **E** Immunohistochemistry was used to detect Ki-67, verifying that tumor cells in the LV-shLINC01016 group exhibited a lower positivity rate than the LV-NC group (magnification: 200×). **F** Tumors from mice in the LV- shLINC01016 group were well-encapsulated in fibrotic capsules, whereas those from mice in the LV-NC group exhibited local muscular invasion (magnification:200×). **G** LV-NC group had more lung metastases than the LV-shLINC01016 group, after injecting the transfected cell to the tail vein of the nude mice. Representative images were presented. **H** Few and smaller metastatic foci were observed in the LV-shLINC01016 group compared to the LV-NC group (H&E magnification: 200×). Representative H&E images of lungs isolated from mice. **I** The number of lung metastatic lesions was calculated. Micrometastases were counted in 20 high-power fields per specimen. **J** Levels of p-AKT, bcl-2, and MMP-9 were measured in murine xenograft tumors. **K** A mechanistic model of the role of LINC01016 in BC. ETS-1 promotes LINC01016 upregulation, leading to the inhibition of RFFL-mediated DHX9 ubiquitination and subsequent proteasomal degradation, resulting in enhanced PI3K/AKT signaling. Data are presented as the means ± SEM. *P < 0.05, **P < 0.01,***P < 0.001.

To explore whether knocking down LINC01016 suppressed BC growth and invasion in vivo, we used a xenograft model in which mice were implanted with LINC01016-silenced BC cells, as confirmed by qRT-PCR (Supplementary Fig. 1D). The results showed that the tumor size of the LV-shLINC01016 group significantly decreased compared to the LV-NC group (Fig. 6A, B) (tumor weight, 1.89 ± 0.35 g vs. 0.92 ± 0.26 g, *P* = 0.0495, Fig. 6C; tumor volume, 2,308.83 ± 156.49 mm3 vs. 1,056.50 ± 198.12 mm3, *P* = 0.0006, Fig. 6D). Immunohistochemical staining for Ki-67 revealed that BC cells in the LV-shLINC01016 group had lower proliferative activity compared with those in the LV-NC group (Fig. 6E). These results supported the hypothesis that LINC01016 promoted BC proliferation in vivo. We also observed that subcutaneous xenograft tumors in the LV-shLINC01016 group were non-invasive or well-encapsulated, whereas tumors in the LV-NC group showed local invasion, with cancer cells invading the surrounding muscle tissue (Fig. 6F). Using a hematogenous metastasis model, in vivo imaging revealed that the LV-shLINC01016 group presented fewer pulmonary metastatic lesions than the LV-NC group (Fig. 6G). The same conclusion was obtained via H&E staining (Fig. 6H, I).

Finally, we explored the effect of knocking down LINC01016 on the expression of DHX9 and various molecules in the PI3K/AKT signaling pathway in xenograft tumors. The results showed that DHX9 protein expression significantly decreased, while the expression of MMP9, p-AKT, and Bcl-2 in the PI3K/AKT signaling pathway decreased remarkably in the LV-shLINC01016 group (Fig. 6J). In summary, when LINC01016 was knocked down, tumor proliferation, invasion, and distant metastasis were significantly inhibited, suggesting that LINC01016 could be used as a potential therapeutic target for TNBC.

## Discussion

Recent studies have shown that lncRNAs can modulate tumorigenesis via diverse mechanisms, including epigenetic modifications and pre- or post-transcriptional regulation of gene expression [21–23]. The specific roles of individual lncRNAs in BC, however, remain unclear. In this study, we leveraged our previous microarray-based analysis of lncRNAs involved in GC progression and identified LINC01016 as a potential oncogenic regulator of BC progression. We discovered that LINC01016 is upregulated in BC tissues with LNM, including TNBC. We further clarified that upregulated LINC01016 levels were correlated with higher Ki67 indices and positive LNM. In the TNBC subtype, patients with high expression of LINC01016 have had shorter RFS and OS.

Previous studies have implicated LINC01016 in breast, thyroid, and endometrial cancers [12, 13, 24]. In breast and thyroid cancer, LINC01016 has been shown to be an indicator of poor prognosis, although its underlying mechanism remains elusive. In endometrial carcinoma, LINC01016 has been shown to function as a ceRNA, and upregulated LINC01016 promoted proliferation, migration, and invasion, which is concordant with our findings [24]. However, the molecular mechanism by which LINC01016 is upregulated in these cancers has not been elucidated. This is the first study to explore the upstream mechanism of the abnormal expression of LINC01016. We found that ETS-1, as an upstream regulator, activated the transcription of LINC01016 and significantly promoted cell proliferation, migration, and invasion. Subcellular localization analyses can offer insights into the functional role of individual lncRNAs within cells. Cytoplasmic lncRNAs are more likely to serve as regulators of mRNA translation and stability, whereas nuclear lncRNAs may play a regulatory role by binding to nucleoproteins [25–27]. In this study, we found that LINC01016 was primarily located in the BC cell nucleus, and its 717–1217 nt region was physically bound to the DHX9 nuclear protein.

DHX9, an NTP-dependent DNA and RNA helicase, is dysregulated in a variety of cancers [28, 29]. For example, Cao *et al*. showed that DHX9 was upregulated in lung cancer and that it contributed to the growth of tumor cells [30]. However, the role of DHX9 in BC remains unclear. In this study, we found that DHX9 overexpression was associated with increased cell proliferation, migration, and invasion, as well as reduced cell apoptosis. In contrast, knockdown of this helicase induced the opposite effect. Additionally, we demonstrated that DHX9 knockdown reduced cell proliferation, migration, and invasion in BC cells with upregulated LINC01016 BC cells. Silencing of DHX9 reversed the apoptotic resistance of LINC01016-overexpressing BC cells, suggesting that LINC01016 enhanced BC cell pathogenicity in a DHX9-dependent manner.

From a functional perspective, we found that LINC01016 upregulated intracellular DHX9 protein levels without affecting DHX9 mRNA expression. We hypothesized that LINC01016 is a post-translational regulator of DHX9. There are many different post-translational modifications that can influence protein function and stability, and ubiquitination is a well-studied method of DHX9 protein regulation. A number of different DHX9 residues can undergo ubiquitination, including lysines 1048, 146, 191, 417, 857, and 1037 (https://www.genecards.org/). In this study, we found that LINC01016 overexpression was associated with reduced DHX9 ubiquitination in BC cells, whereas the opposite result was observed when LINC01016 was knocked down. By Co-IP and MS analyses, we further identified that RFFL, an E3 ubiquitin ligase, was responsible for DHX9 ubiquitination. RFFL has previously been shown to induce the ubiquitination and subsequent proteasomal degradation of a range of target proteins [31, 32]. Here, we found that RFFL overexpression was sufficient to reduce DHX9 protein levels through a mechanism of ubiquitination and subsequent proteasome-dependent degradation. We surmised that LINC01016 promoted BC cell malignancy via a DHX9- and RFFL-mediated ubiquitination-dependent mechanism. LINC01016 is physically bound to DHX9, markedly impaired co-precipitation between DHX9 and RFFL, and sequestered DHX9 from RFFL, thereby preventing the ubiquitination of DHX9 and subsequent degradation.

Through mRNA sequencing, we found that DHX9 may exert its malignant biological behavior by activating the PI3K/AKT signaling pathway. Western blotting analysis showed that overexpression of LINC01016 increased the expression of DHX9, MMP-9, p-AKT, and Bcl-2. The specific mechanism by which LINC01016 promoted the phosphorylation of AKT will be explored in the future.

In summary, we showed that ETS-1-induced upregulation of LINC01016 enhanced the proliferation, migration, apoptotic resistance, and cell cycle progression of BC cells. Following induction, LINC01016 inhibited RFFL-mediated DHX9 ubiquitination via competitive binding, thus protecting this helicase from proteasomal degradation and ultimately enhancing PI3K/AKT signaling (Fig. 6K). LINC01016 and LINC01016-mediated DHX9/PI3K/AKT signaling may thus be viable targets for the treatment of TNBC patients.

## Supplementary information

Table S1 primers

Supplementary Figure

Supplementary Table 2 DHX9

Supplementary Table 3 RFFL

Supplementary Table 4 pathway Result

western blots

A reproducibility checklist

## Data Availability

The data that support the findings of our study are presented in the paper. The rest datasets used or analyzed during the current study are available from the corresponding author on reasonable request.
