# Supplementary material for: ETS-1-activated LINC01016 over-expression promotes tumor progression via suppression of RFFL-mediated DHX9 ubiquitination degradation in breast cancers
Source: Cell Death Dis. 2023 Aug 8;14(8):507. doi: 10.1038/s41419-023-06016-3 (PMC10406855; doi:10.1038/s41419-023-06016-3)
Supplement: Supplementary file 1 — Table S1 primers [file 41419_2023_6016_MOESM1_ESM.pdf]

Supplementary table 1

Name	Sense primer (5′- to 3′)	Antisense primer (5′- to 3′)
**Primer for coloning**		
LINC01016 sense	ATAAGAATGCGGCCGCGCAGCAAATCCGAACAGGCAAAGGC	CTAGCTAGCTTTTTTTTTTTTTTTGAACTTGCAAATATA
LINC01016 antisense	CTAGCTAGCGCAGCAAATCCGAACAGGCAAAGGC	ATAAGAATGCGGCCGCTTTTTTTTTTTTTTTGAACTTGCAAATATA
-1000/0	GGGGTACCTCTGCCCATTATCGGAGGTGCATCT	CCGCTCGAGGCCTCGTGTGTTTCAAGCTGCCCAC
-750/0	GGGGTACCAGCCTACTAGGGACAGGGGTAGGAC	CCGCTCGAGGCCTCGTGTGTTTCAAGCTGCCCAC
-500/0	GGGGTACCCTTTGGCTAATCAATTTATTTTAAC	CCGCTCGAGGCCTCGTGTGTTTCAAGCTGCCCAC
-250/0	GGGGTACCTCAGGTTGGACTAAATGGTCACTCA	CCGCTCGAGGCCTCGTGTGTTTCAAGCTGCCCAC
-125/0	GGGGTACCAACAAACAAGGGCTGCAAGACTAAA	CCGCTCGAGGCCTCGTGTGTTTCAAGCTGCCCAC
**Primers for CHIP**		
ETS-1	CAGCCTGGAACAAGGGACAG	AACTCTAACATCTGTTCCCAGTAAA
**Primers for qRT-PCR**		
LINC01016	TACAAGGCCCCGTGGATTTC	CCTGTACCTCACAGCACCAG
ETS-1	CCTCCCCGGATATGGAATGTG	CATCACCCAGTCCCGAACAT

Antisense oligonucleotide (ASO) against LINC01016

si-LINC01016-1 : CAGCAGACCGCATTGACGAC;
si-LINC01016-2: GAAGACAGACCGGTAGAGCC;
si-LINC01016-3: GAGTATAGTCCAACCACCAC;
si-LINC01016-4 : CATCAGAAAGCAGGGAGCAC;
