# Supplementary material for: ETS-1-activated LINC01016 over-expression promotes tumor progression via suppression of RFFL-mediated DHX9 ubiquitination degradation in breast cancers
Source: Cell Death Dis. 2023 Aug 8;14(8):507. doi: 10.1038/s41419-023-06016-3 (PMC10406855; doi:10.1038/s41419-023-06016-3)
Supplement: Supplementary file 2 — Supplementary Figure [file 41419_2023_6016_MOESM2_ESM.pdf]

Supplementary information

ETS-1-activated LINC01016 over-expression promotes tumor progression via suppression of RFFL-mediated DHX9 ubiquitination degradation in breast cancers

Sup.Fig.1

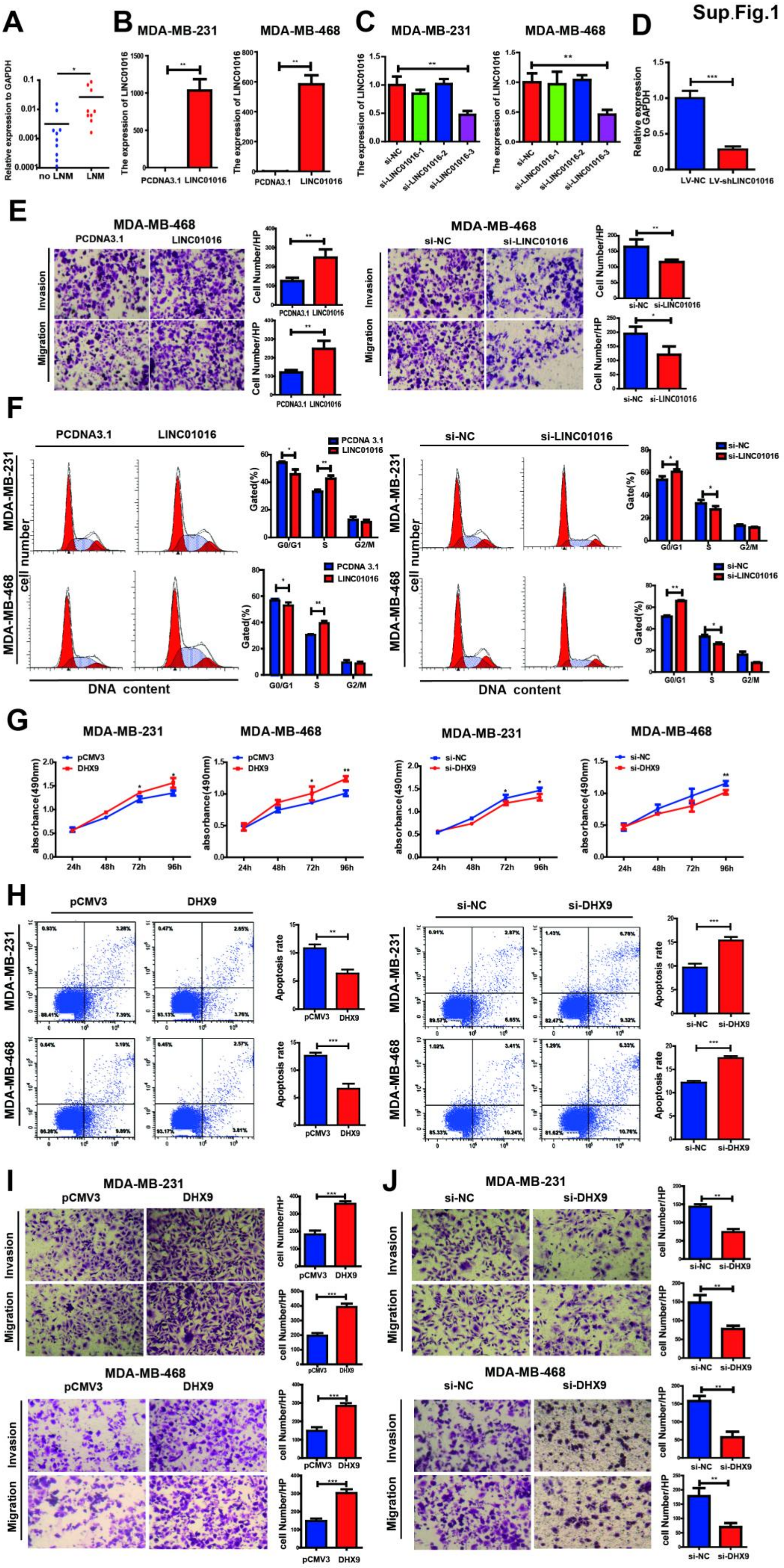
A
Relative expression to GAPDH
no LNM
LNM
B
MDA-MB-231
MDA-MB-468
The expression of LINC01016
PCDNA3.1
LINC01016
C
MDA-MB-231
MDA-MB-468
The expression of LINC01016
si-NC
si-LINC01016-1
si-LINC01016-2
si-LINC01016-3
D
Relative expression to GAPDH
LV-NC
LV-shLINC01016
E
MDA-MB-468
PCDNA3.1
LINC01016
Invasion
Migration
Cell Number/HP
si-NC
si-LINC01016
F
PCDNA3.1
LINC01016
MDA-MB-231
MDA-MB-468
cell number
DNA content
PCDNA 3.1
Gated(%)
G0/G1
S
G2/M
si-NC
si-LINC01016
G
MDA-MB-231
MDA-MB-468
pCMV3
DHX9
si-NC
si-DHX9
absorbance(490nm)
24h
48h
72h
96h
H
pCMV3
DHX9
si-NC
si-DHX9
MDA-MB-231
MDA-MB-468
Apoptosis rate
I
MDA-MB-231
pCMV3
DHX9
Invasion
Migration
cell Number/HP
MDA-MB-468
J
MDA-MB-231
si-NC
si-DHX9
MDA-MB-468

Supplementary Fig. 1. A LINC01016 expression was 8.3-fold higher in TNBC patients with LNM than those without LNM. **B** The efficiency of LINC01016 up-regulation in MDA-MB-231 and MDA-MB-468 cells was verified via qRT-PCR. **C** Three different Antisense oligonucleotides (ASOs) specific for LINC01016 (si-LINC01016-1, si-LINC01016-2, and si-LINC01016-3) were obtained from RiboBio. Of the three tested ASO constructs, si-LINC01016-3 successfully reduced LINC01016 expression by > 60%. **D** The qRT-PCR assay showed that LINC01016 was stably knocked down in the LV-shLINC01016-transfected cells. **E** LINC01016 overexpression enhanced the migration and invasion of MDA-MB-468 cells in transwell assays, while silencing suppressed both of these activities. **F** Elevated LINC01016 expression increases the number of G0/G1 cells entering the S phase of the cell cycle, while knockdown of this lncRNA is associated with G0/G1 phase arrest. **G** Overexpression of DHX9 promoted cell proliferation, which was inhibited when DHX9 was knockdown in MTS assays. **H** Flow cytometry revealed that DHX9 overexpression reduced the rate of cell apoptosis, which was increased when DHX9 was konckdown. **I, J** DHX9 overexpression enhanced the migration and invasion of BC cells in transwell assays, while silencing suppressed both of these activities. Data are presented as means ± SD. *P < 0.05, **P < 0.01,***P < 0.001.

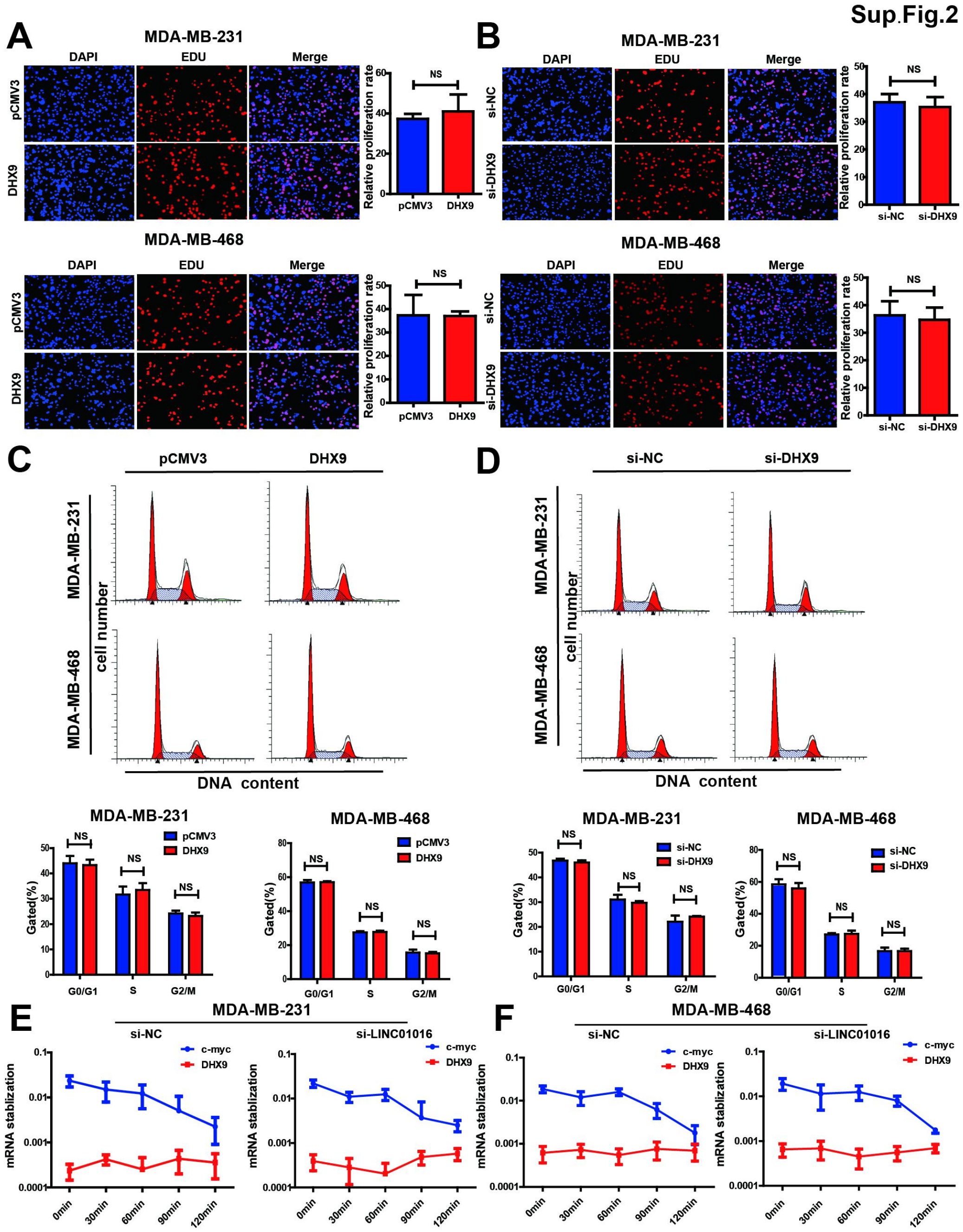

Supplementary Fig. 2 A The results of the EdU incorporation assay showed that DHX9 overexpression had no significant effect on cell proliferation. **B** DHX9 knockdown did not alter BC cell proliferation as measure in an EdU incorporation assay. **C** Flow cytometry displayed that DHX9 upregulation had no significant effect on cell cycle progression in BC cells. **D** DHX9 knockdown did not alter BC cell cycle distributions as measure via flow cytometry. **E, F** DHX9 mRNA stability was

assessed via qRT-PCR in cells knockdown of LINC01016 and treated with Actinomycin D (20μM) for 0, 30, 60, 90 and 12 0min. Knockdown of LINC01016 had no effect on DHX9 mRNA expression levels. Data are shown as mean ± SD, *P < 0.05, **P < 0.01, ***P < 0.001. *ns.* not significant.
