# Supplementary figures and images for: ETS-1-activated LINC01016 over-expression promotes tumor progression via suppression of RFFL-mediated DHX9 ubiquitination degradation in breast cancers
Source: Cell Death Dis. 2023 Aug 8;14(8):507. doi: 10.1038/s41419-023-06016-3 (PMC10406855; doi:10.1038/s41419-023-06016-3)

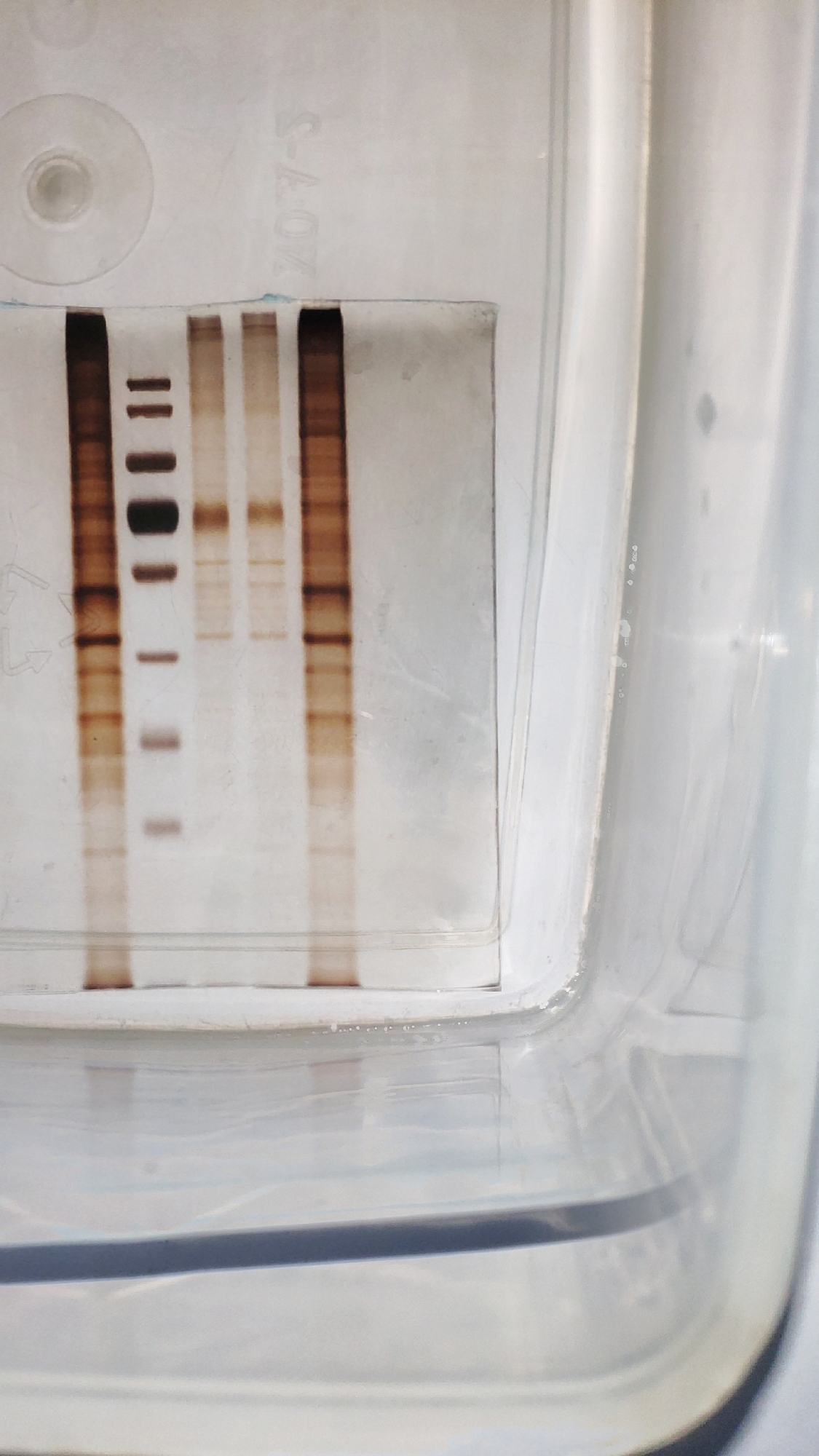

Supplement: Supplementary file 6 — western blots [file 41419_2023_6016_MOESM6_ESM.zip › breast cancer WB/Figure 4A/MDA-MB-231.tif]

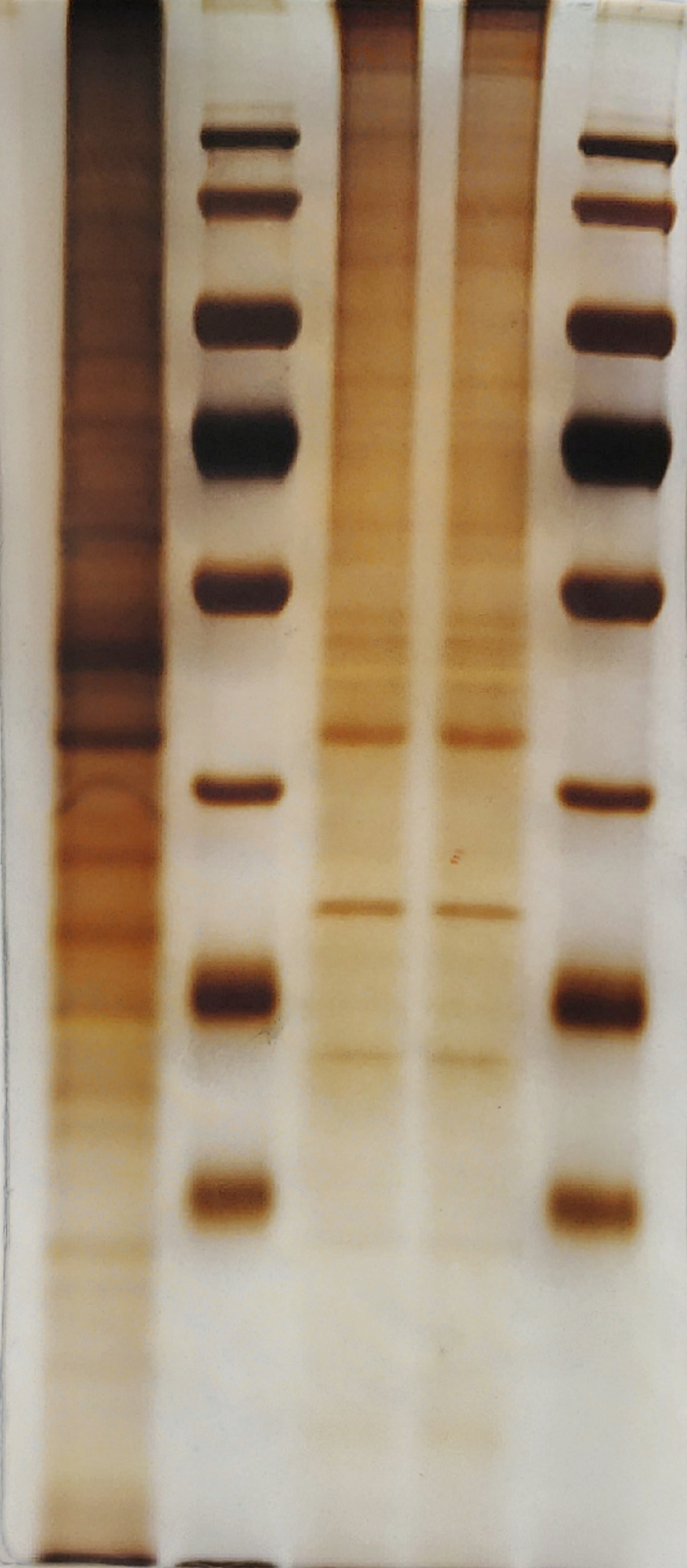

Supplement: Supplementary file 6 — western blots [file 41419_2023_6016_MOESM6_ESM.zip › breast cancer WB/Figure 4A/MDA-MB-468.tif]

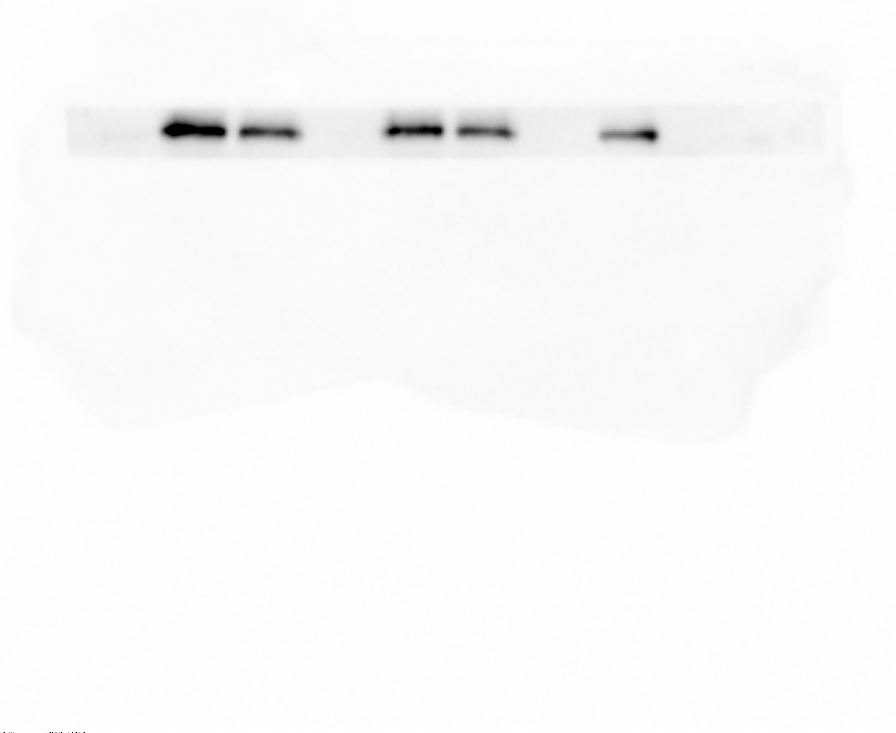

Supplement: Supplementary file 6 — western blots [file 41419_2023_6016_MOESM6_ESM.zip › breast cancer WB/Figure 4B/DHX9 MDA-MB-231 (1-3);MDA-MB-468 (4-6).tif]

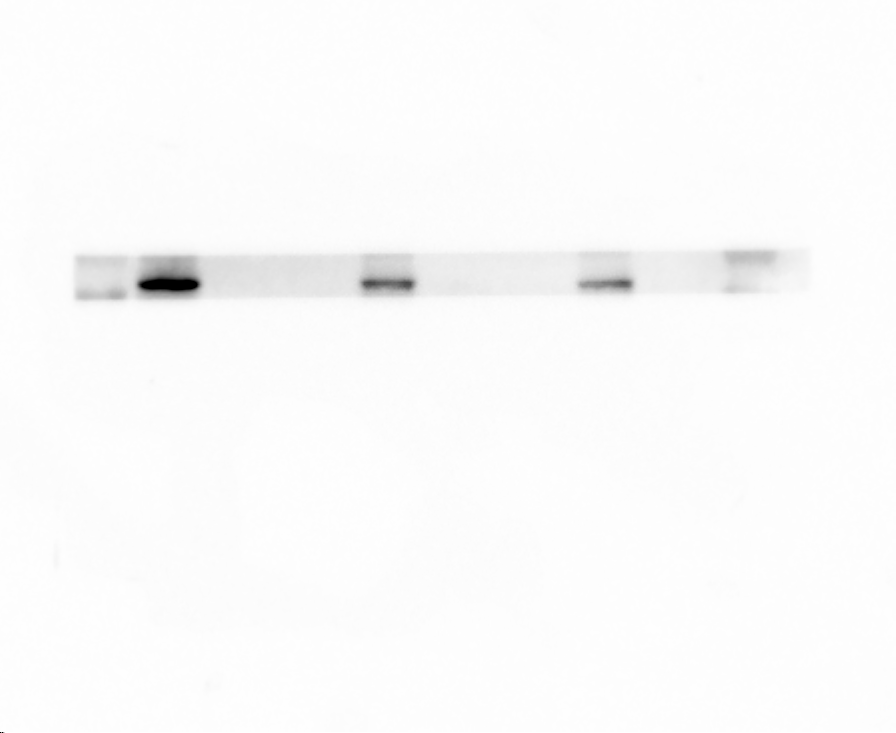

Supplement: Supplementary file 6 — western blots [file 41419_2023_6016_MOESM6_ESM.zip › breast cancer WB/Figure 4B/GADPH MDA-MB-231(1-3);MDA-MB-468(4-6).tif]

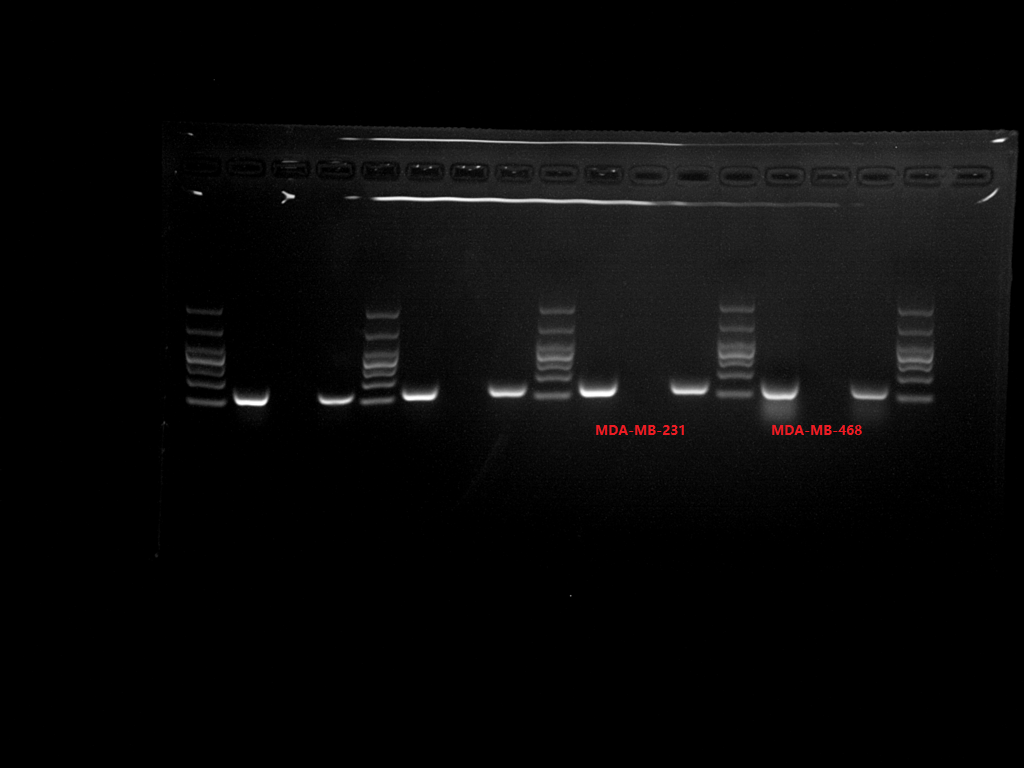

Supplement: Supplementary file 6 — western blots [file 41419_2023_6016_MOESM6_ESM.zip › breast cancer WB/Figure 4C/Figure 4C.png]

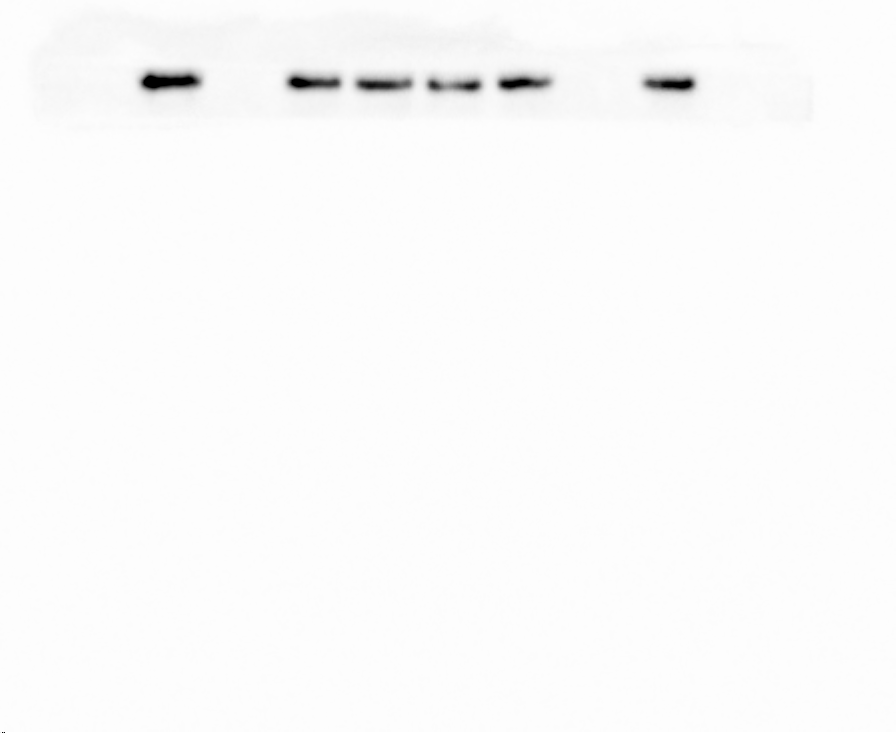

Supplement: Supplementary file 6 — western blots [file 41419_2023_6016_MOESM6_ESM.zip › breast cancer WB/Figure 4D/231 DHX9.tif]

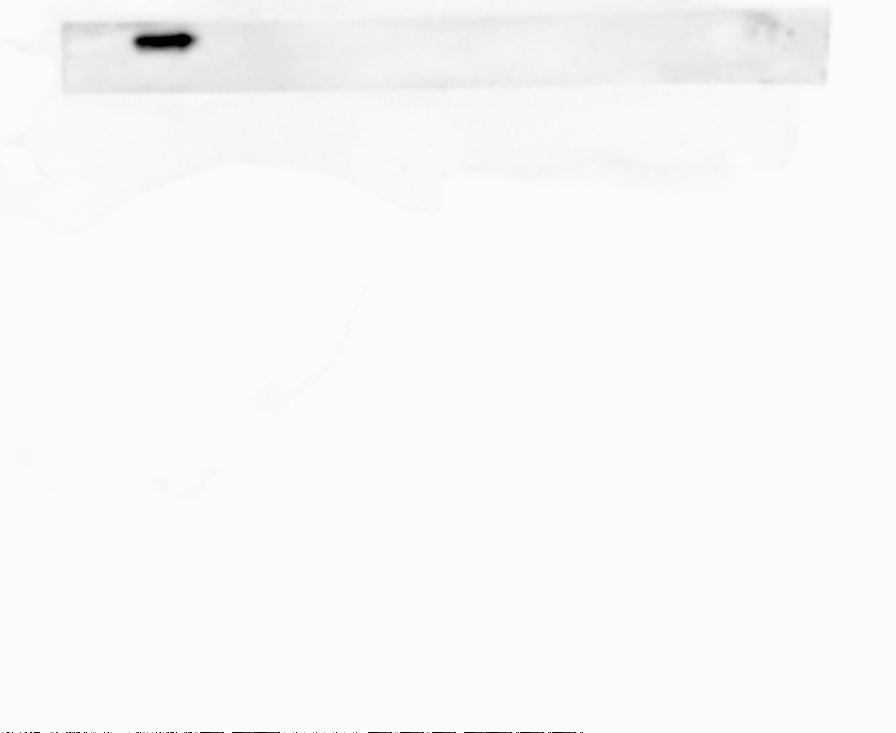

Supplement: Supplementary file 6 — western blots [file 41419_2023_6016_MOESM6_ESM.zip › breast cancer WB/Figure 4D/231 GADPH.tif]

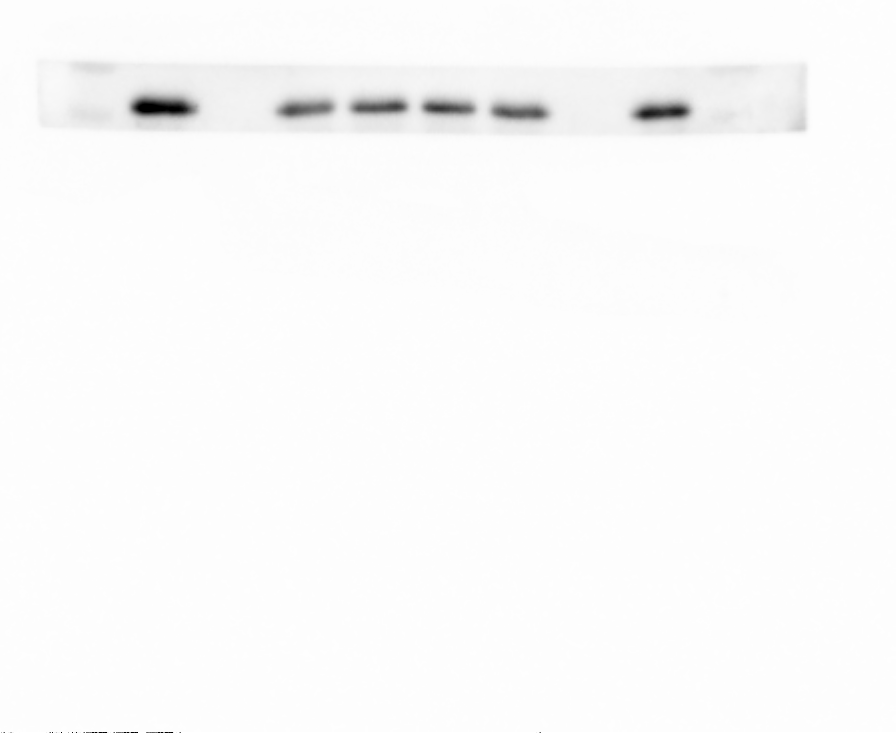

Supplement: Supplementary file 6 — western blots [file 41419_2023_6016_MOESM6_ESM.zip › breast cancer WB/Figure 4D/468 DHX9.tif]

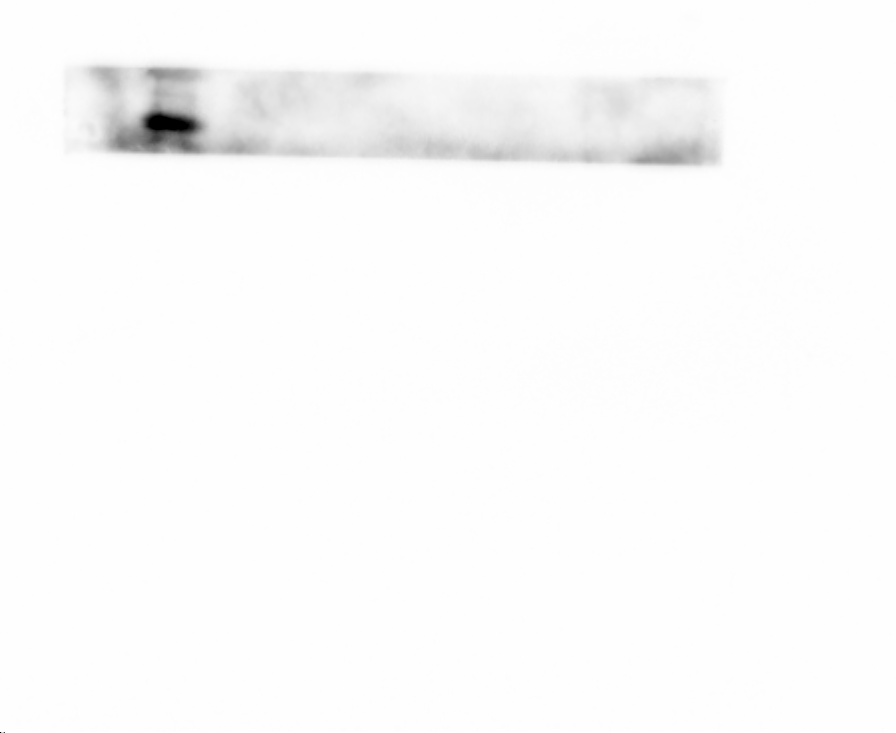

Supplement: Supplementary file 6 — western blots [file 41419_2023_6016_MOESM6_ESM.zip › breast cancer WB/Figure 4D/468 GADPH.tif]

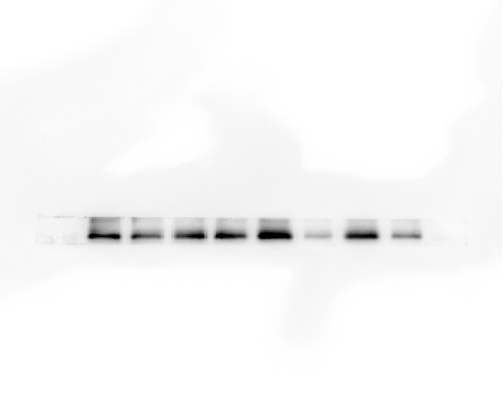

Supplement: Supplementary file 6 — western blots [file 41419_2023_6016_MOESM6_ESM.zip › breast cancer WB/Figure 5A/DHX9 si-01016 MDA-MB-231,468 (5-8).png]

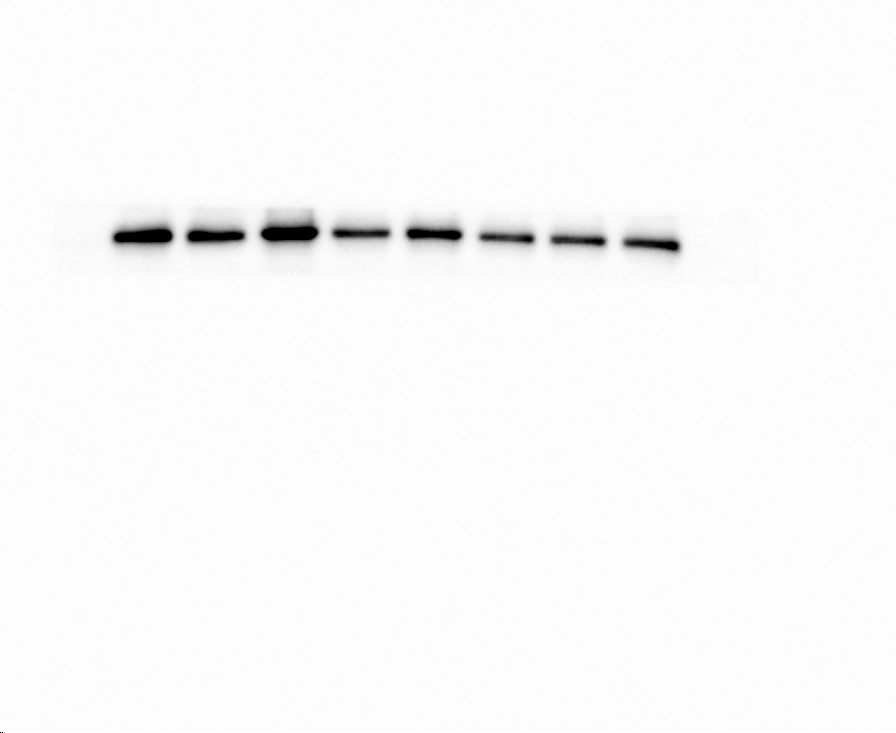

Supplement: Supplementary file 6 — western blots [file 41419_2023_6016_MOESM6_ESM.zip › breast cancer WB/Figure 5A/GADPH si-01016 MDA-MB-231,468 (5-8).tif]

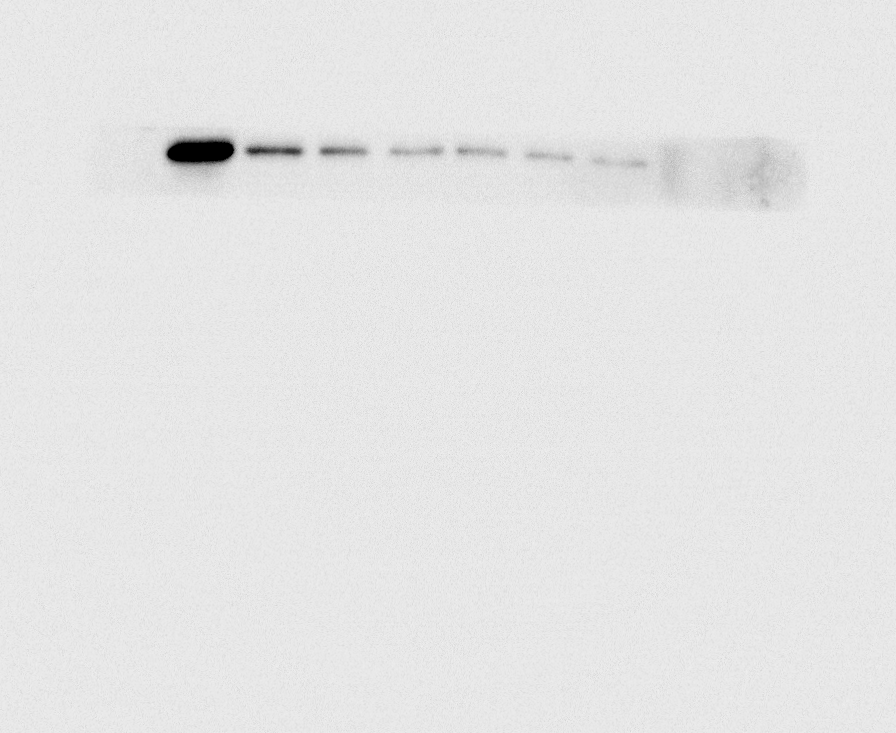

Supplement: Supplementary file 6 — western blots [file 41419_2023_6016_MOESM6_ESM.zip › breast cancer WB/Figure 5B/231cell si-LICN 01016 DHX9(1-7).tif]

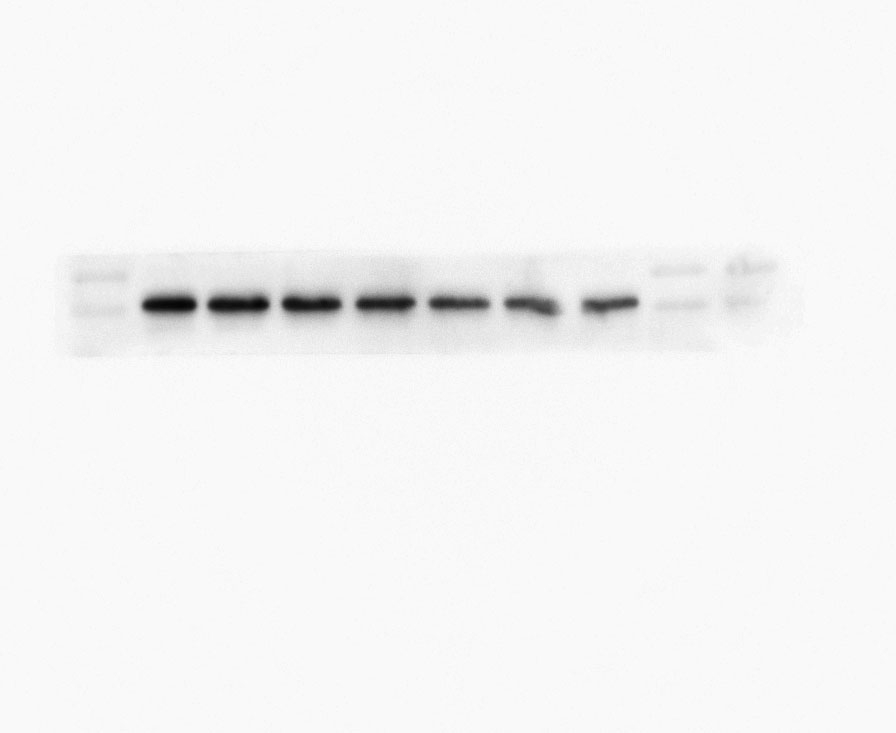

Supplement: Supplementary file 6 — western blots [file 41419_2023_6016_MOESM6_ESM.zip › breast cancer WB/Figure 5B/231cell si-LICN 01016 GADPH(1-7).tiff]

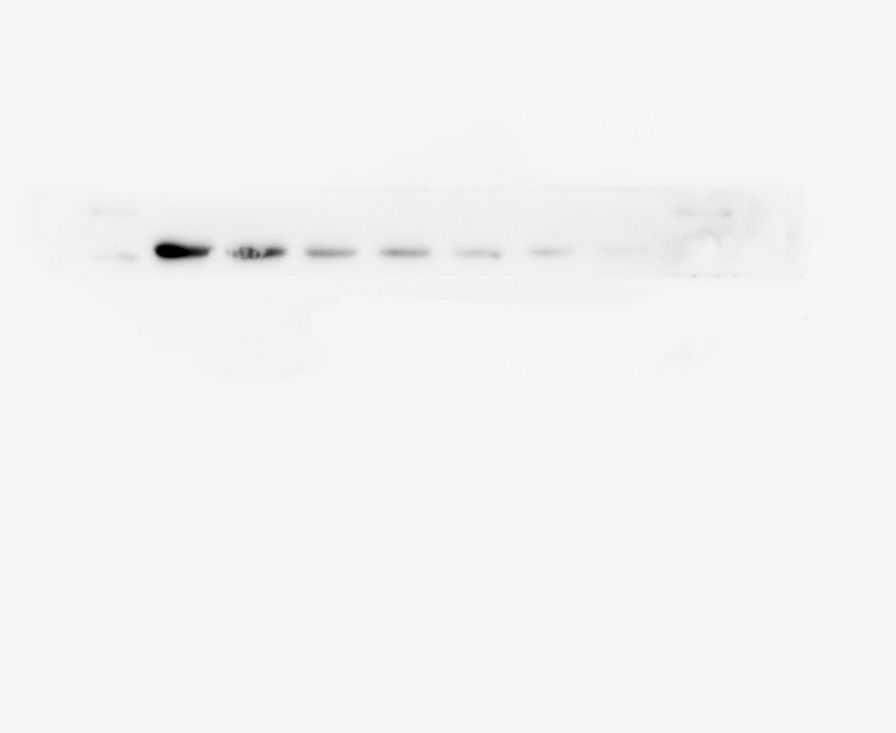

Supplement: Supplementary file 6 — western blots [file 41419_2023_6016_MOESM6_ESM.zip › breast cancer WB/Figure 5B/231cell si-NC DHX9(1-7).tif]

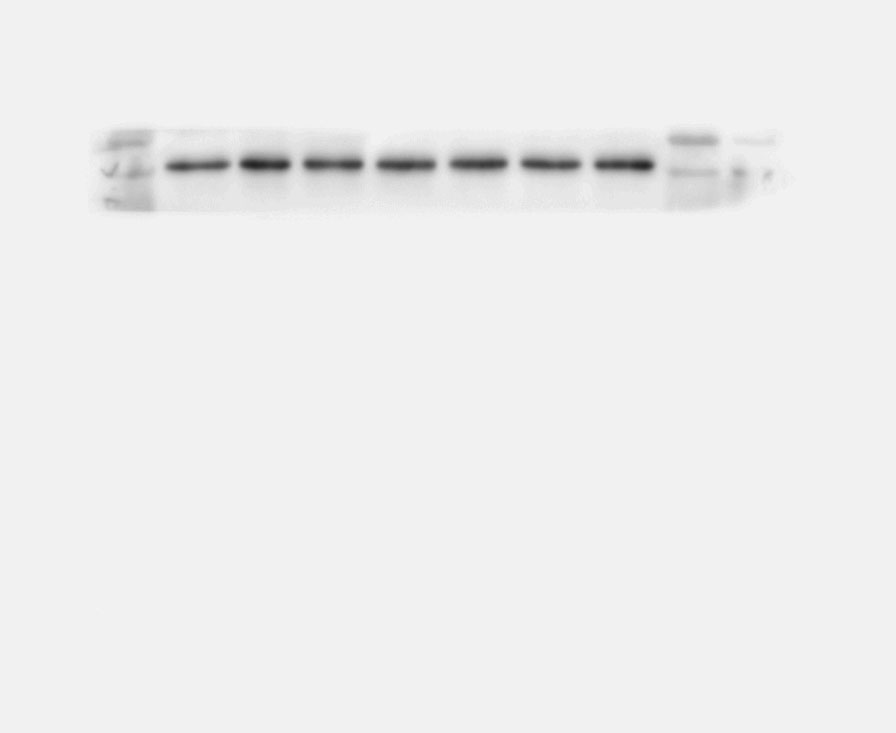

Supplement: Supplementary file 6 — western blots [file 41419_2023_6016_MOESM6_ESM.zip › breast cancer WB/Figure 5B/231cell si-NC GADHP(1-7).tiff]

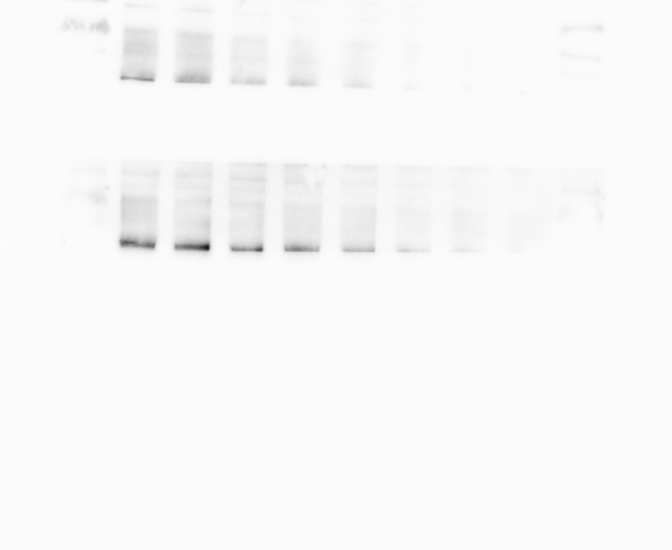

Supplement: Supplementary file 6 — western blots [file 41419_2023_6016_MOESM6_ESM.zip › breast cancer WB/Figure 5B/468 cell si-LICN 01016 DHX9(1-7).tif]

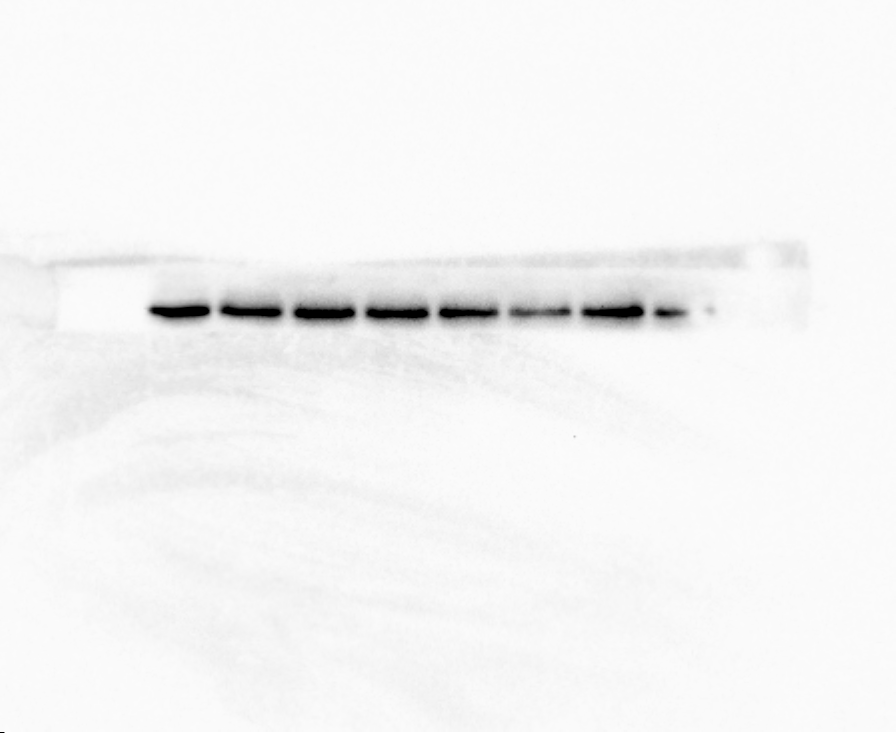

Supplement: Supplementary file 6 — western blots [file 41419_2023_6016_MOESM6_ESM.zip › breast cancer WB/Figure 5B/468cell si-LINC01016 GADHP(1-7).tif]

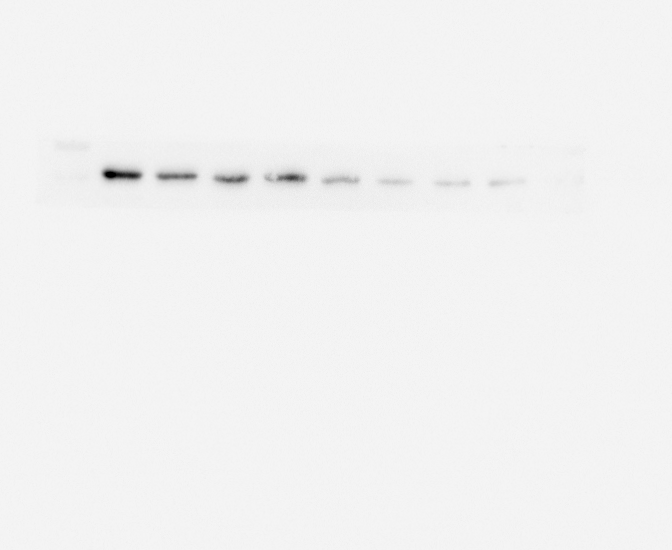

Supplement: Supplementary file 6 — western blots [file 41419_2023_6016_MOESM6_ESM.zip › breast cancer WB/Figure 5B/468cell si-NC DHX9(1-7).tif]

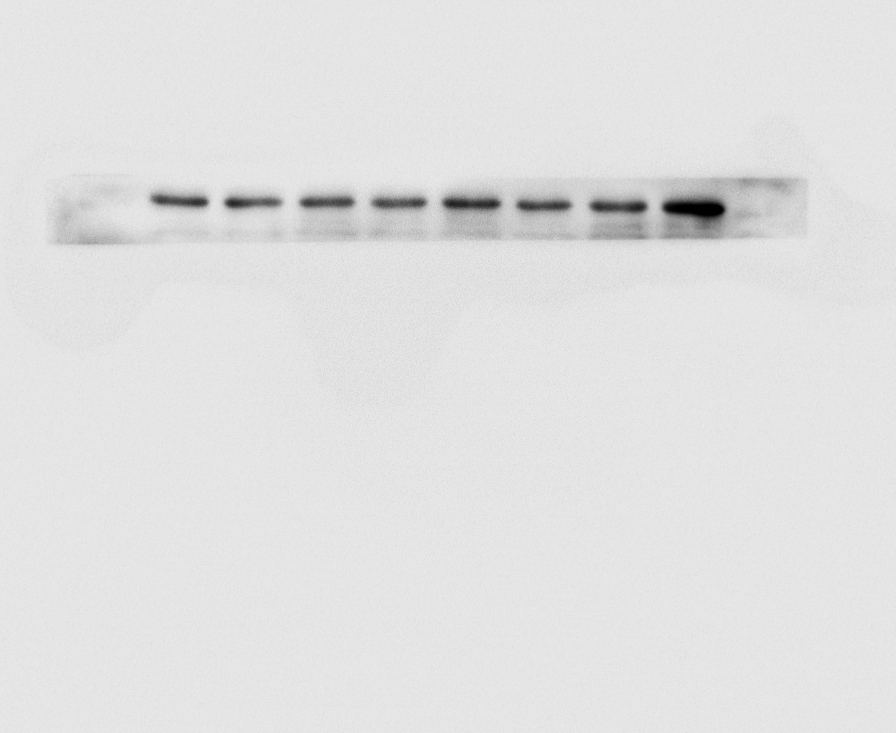

Supplement: Supplementary file 6 — western blots [file 41419_2023_6016_MOESM6_ESM.zip › breast cancer WB/Figure 5B/468cell si-NC GADHP(1-7).tiff]

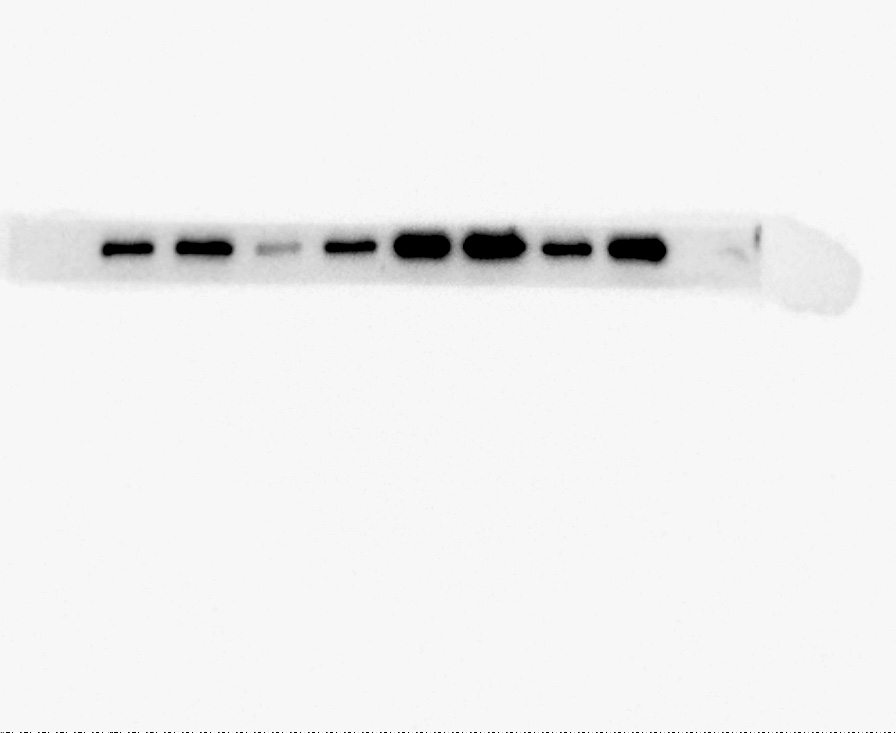

Supplement: Supplementary file 6 — western blots [file 41419_2023_6016_MOESM6_ESM.zip › breast cancer WB/Figure 5C/DHX9 231cell (1-4).jpg]

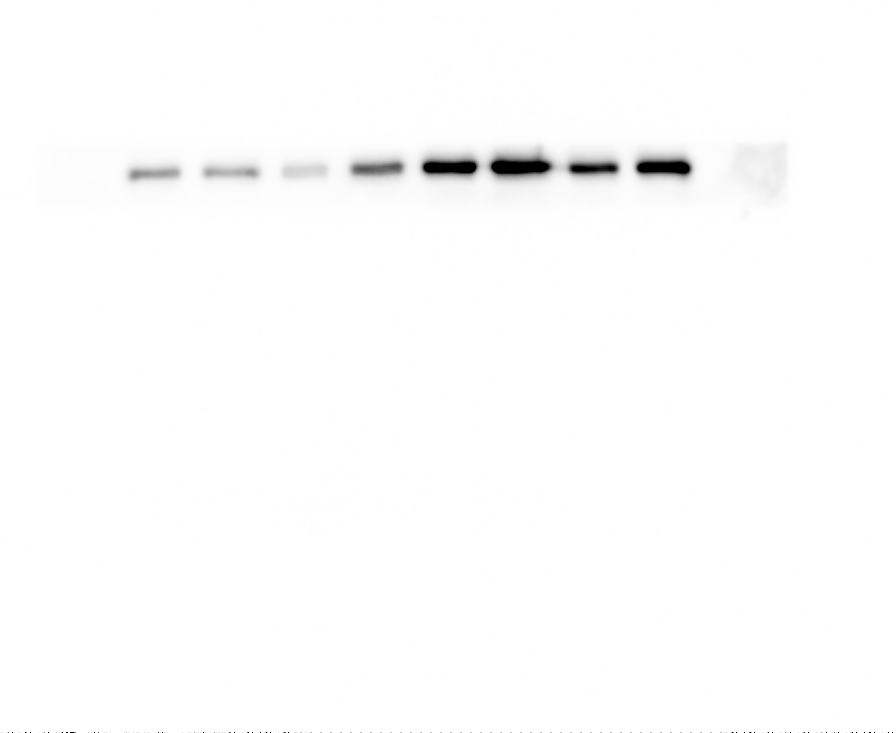

Supplement: Supplementary file 6 — western blots [file 41419_2023_6016_MOESM6_ESM.zip › breast cancer WB/Figure 5C/DHX9 468cell (5-8).tif]

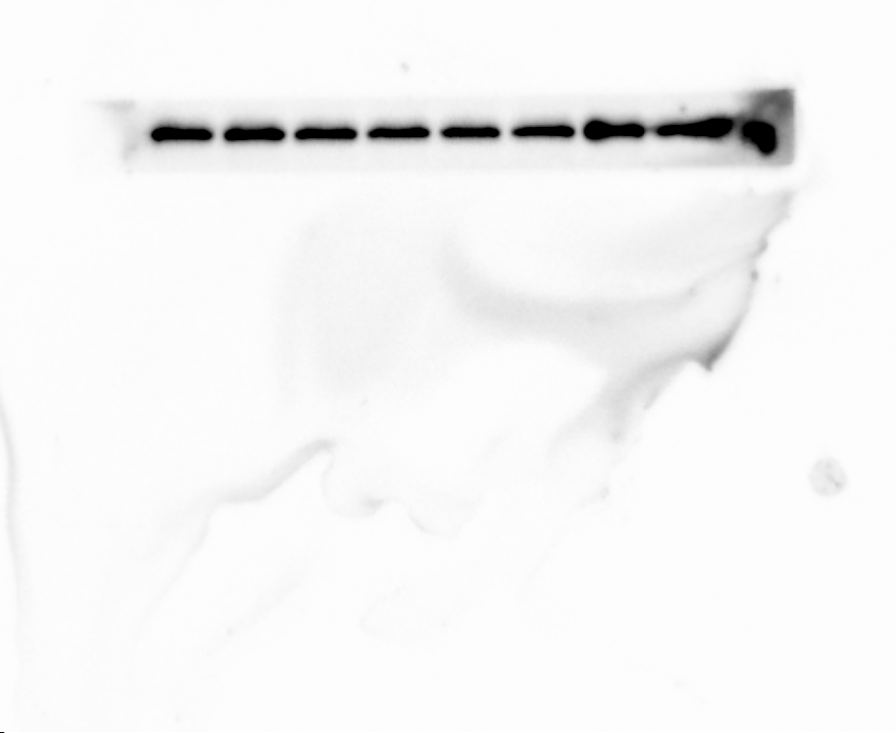

Supplement: Supplementary file 6 — western blots [file 41419_2023_6016_MOESM6_ESM.zip › breast cancer WB/Figure 5C/GADPH 231cell (1-4).tif]

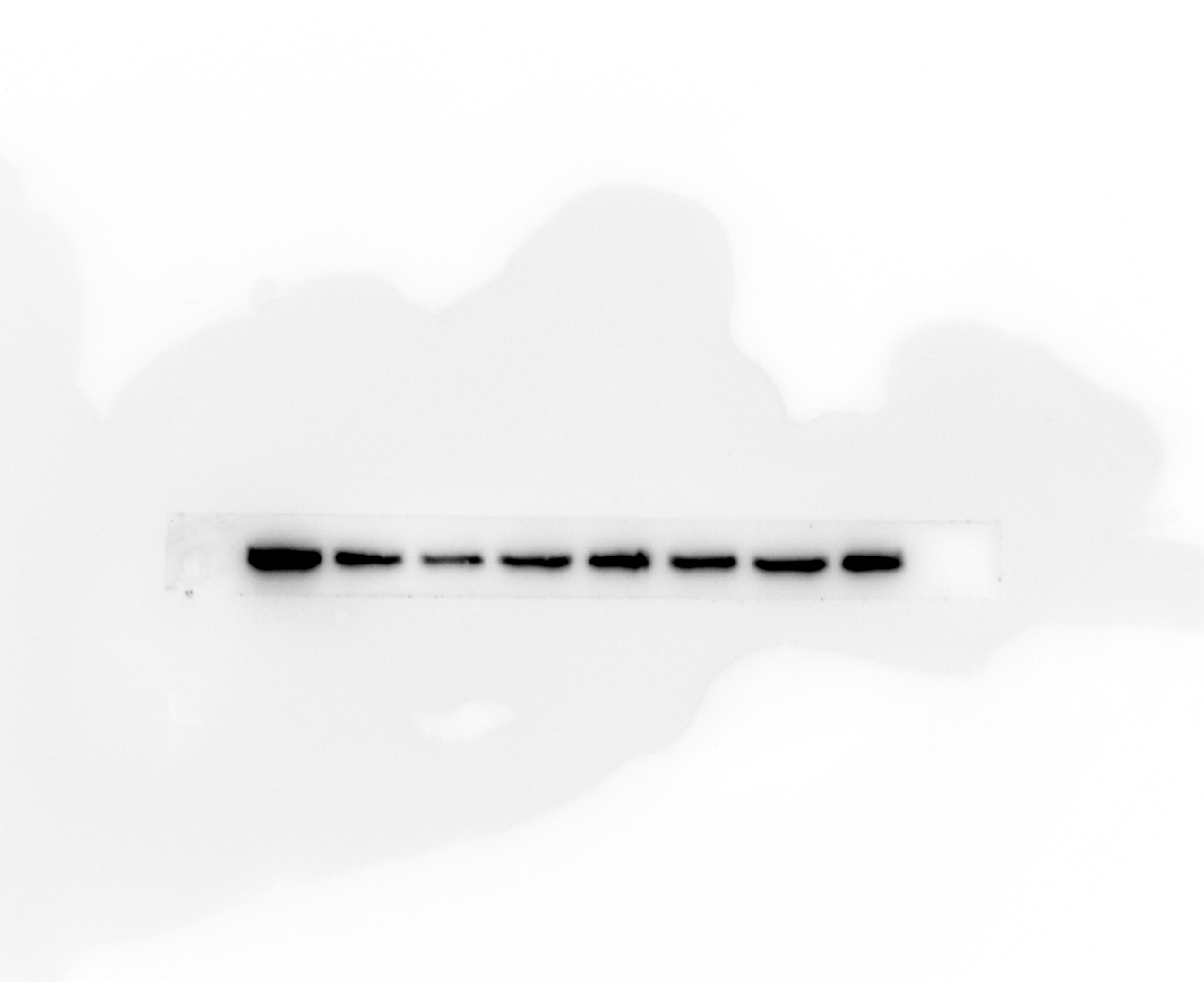

Supplement: Supplementary file 6 — western blots [file 41419_2023_6016_MOESM6_ESM.zip › breast cancer WB/Figure 5C/GADPH 468 cell (5-8).Tif]

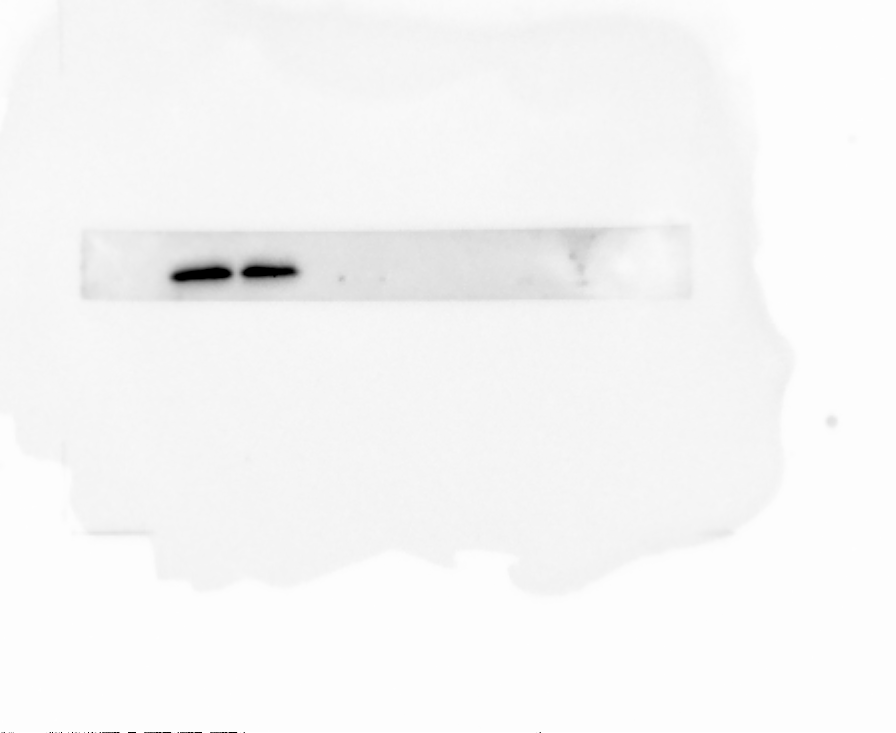

Supplement: Supplementary file 6 — western blots [file 41419_2023_6016_MOESM6_ESM.zip › breast cancer WB/Figure 5D/231 GADPH.tif]

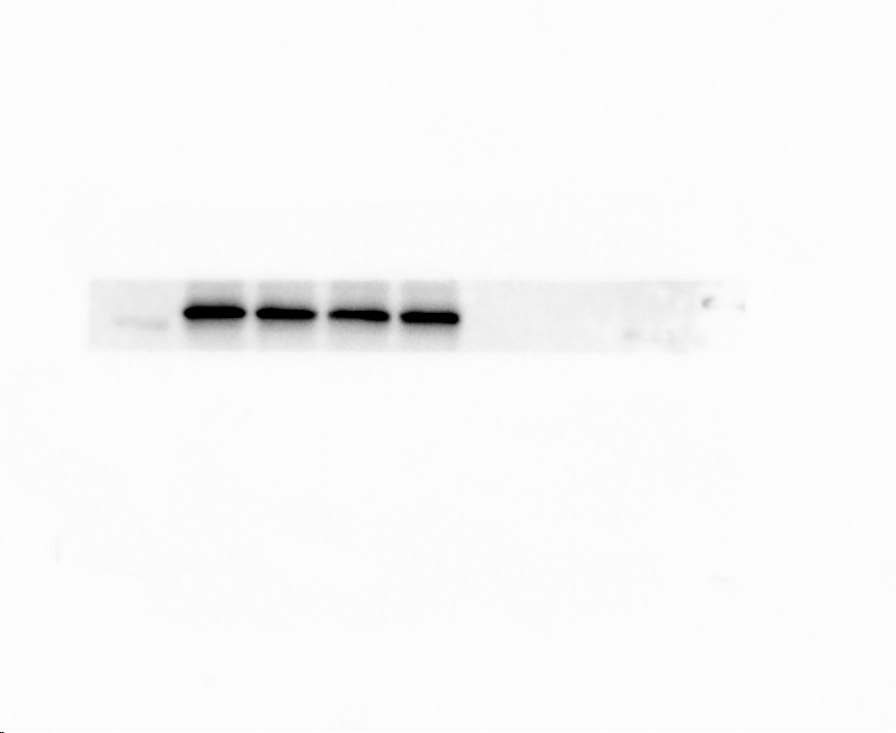

Supplement: Supplementary file 6 — western blots [file 41419_2023_6016_MOESM6_ESM.zip › breast cancer WB/Figure 5D/231 DHX9.tif]

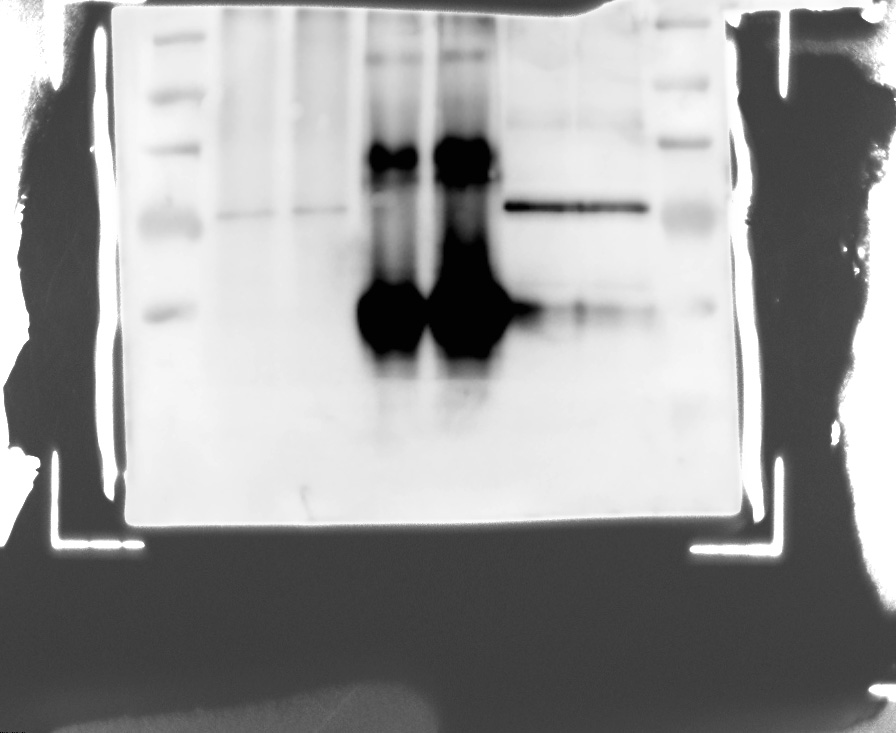

Supplement: Supplementary file 6 — western blots [file 41419_2023_6016_MOESM6_ESM.zip › breast cancer WB/Figure 5D/231cell DHX9.jpg]

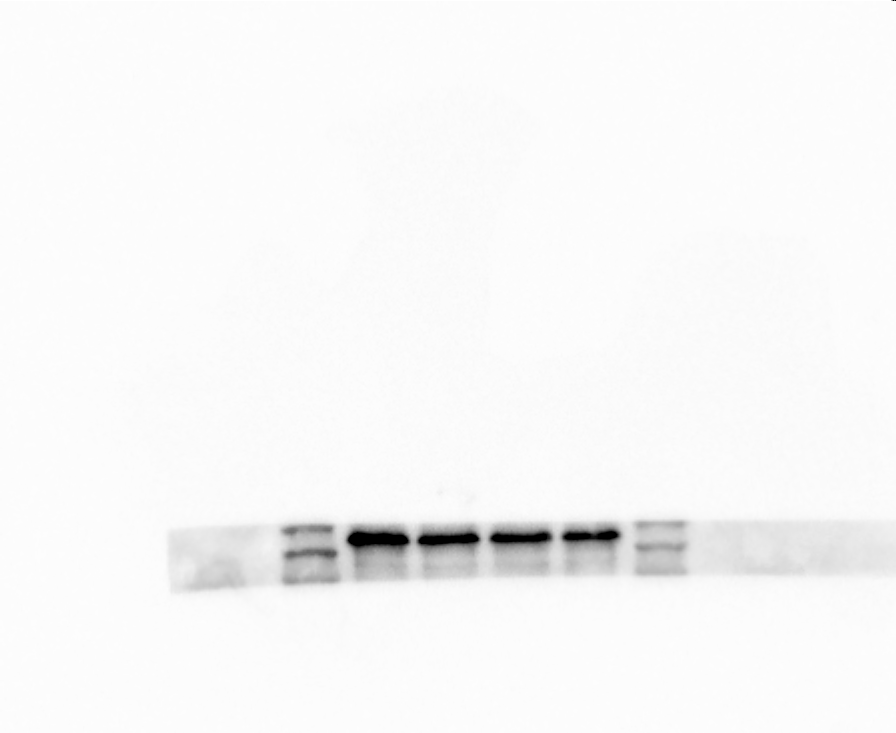

Supplement: Supplementary file 6 — western blots [file 41419_2023_6016_MOESM6_ESM.zip › breast cancer WB/Figure 5D/468 DHX9.tif]

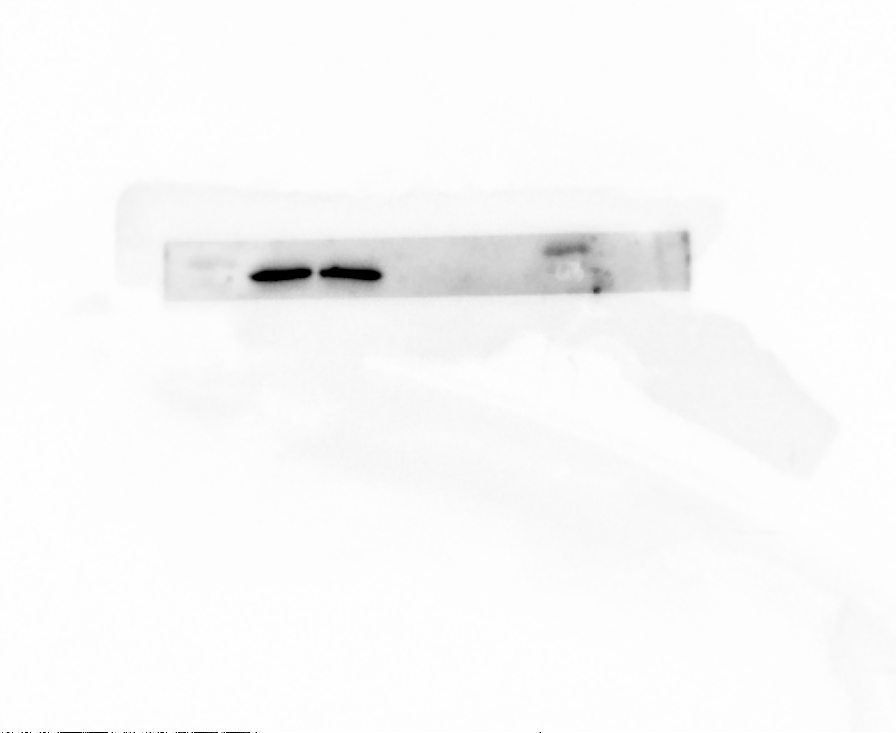

Supplement: Supplementary file 6 — western blots [file 41419_2023_6016_MOESM6_ESM.zip › breast cancer WB/Figure 5D/468 GADPH.tif]

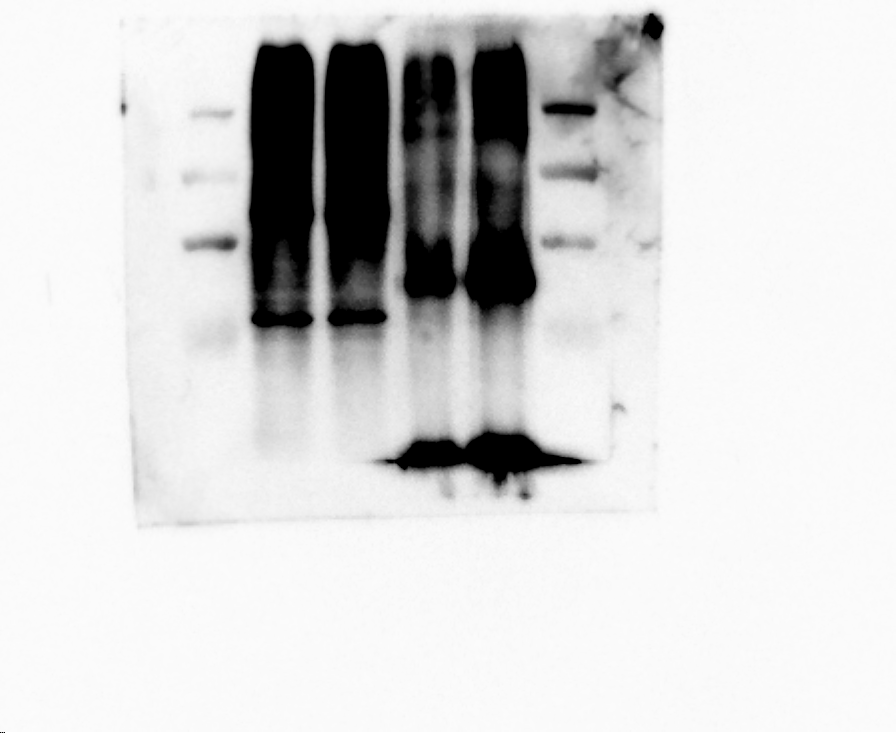

Supplement: Supplementary file 6 — western blots [file 41419_2023_6016_MOESM6_ESM.zip › breast cancer WB/Figure 5D/468cell DHX9.tif]

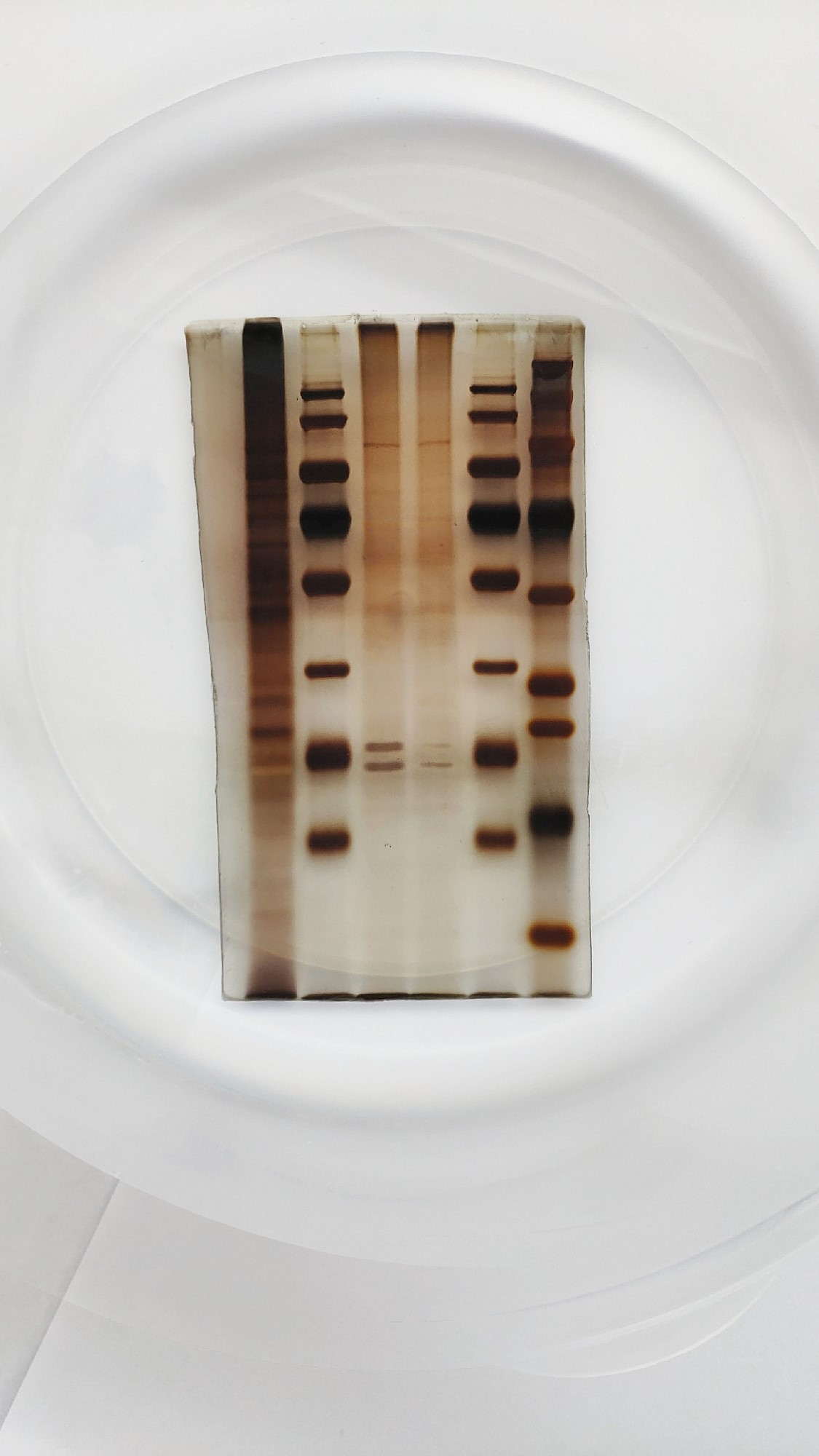

Supplement: Supplementary file 6 — western blots [file 41419_2023_6016_MOESM6_ESM.zip › breast cancer WB/Figure 5E/Figure 5E.tif]

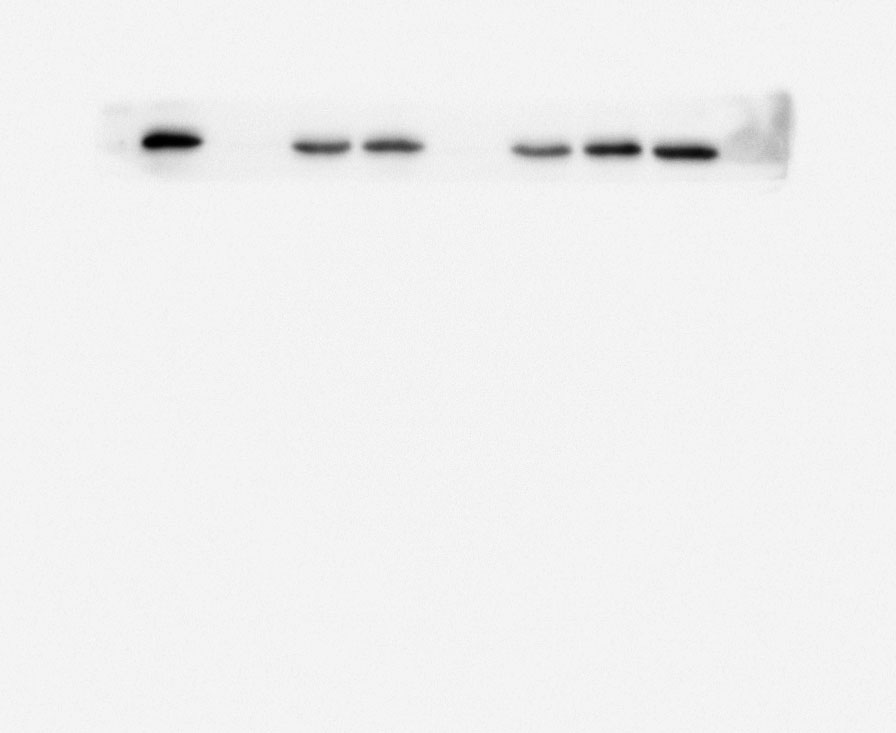

Supplement: Supplementary file 6 — western blots [file 41419_2023_6016_MOESM6_ESM.zip › breast cancer WB/Figure 5F/IP RFFL/IP RFFL, DHX9 231cell (1-3),468cell(4-6).jpg]

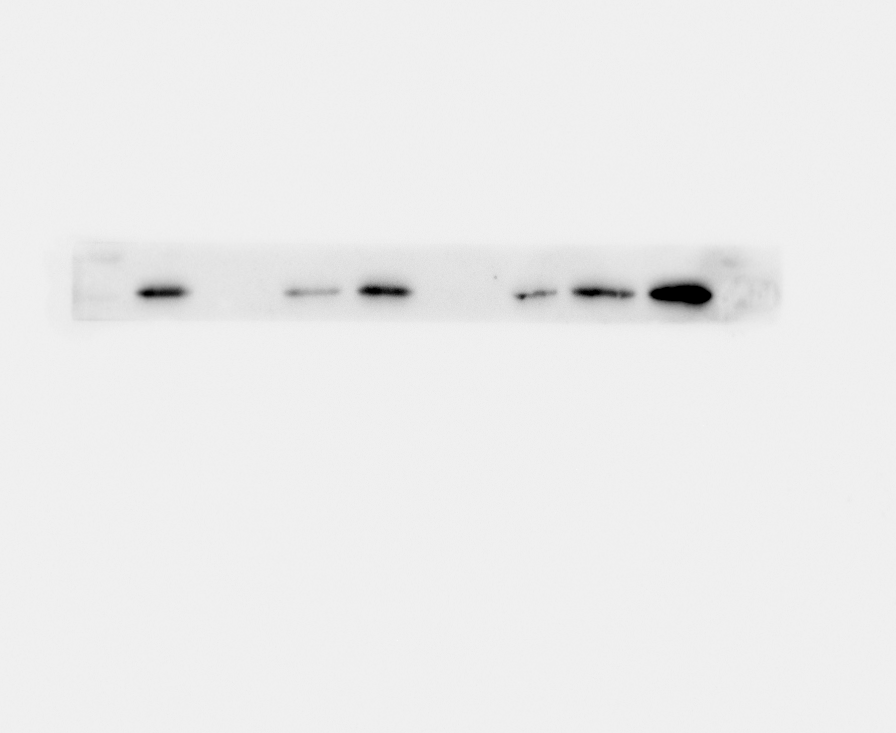

Supplement: Supplementary file 6 — western blots [file 41419_2023_6016_MOESM6_ESM.zip › breast cancer WB/Figure 5F/IP RFFL/IP RFFL, RFFL 231cell (1-3),468cell(4-6).tif]

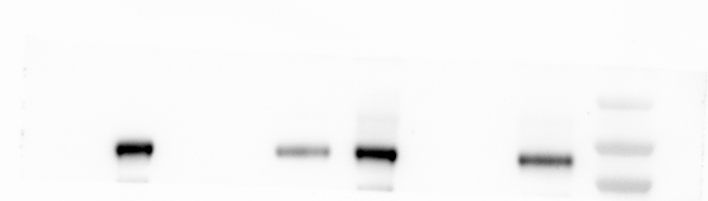

Supplement: Supplementary file 6 — western blots [file 41419_2023_6016_MOESM6_ESM.zip › breast cancer WB/Figure 5F/IP DHX9/IP DHX9, DHX9 231cell (1-3),468cell(4-6).jpg]

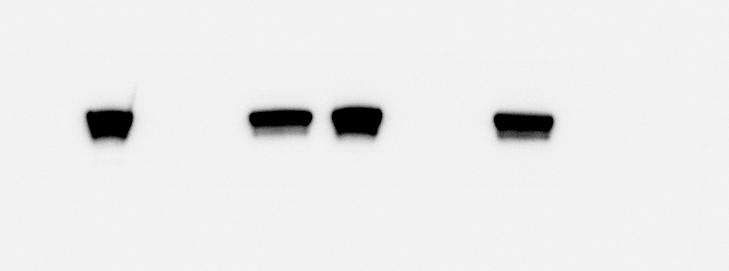

Supplement: Supplementary file 6 — western blots [file 41419_2023_6016_MOESM6_ESM.zip › breast cancer WB/Figure 5F/IP DHX9/IP DHX9, RFFL 231cell (1-3),468cell(4-6).jpg]

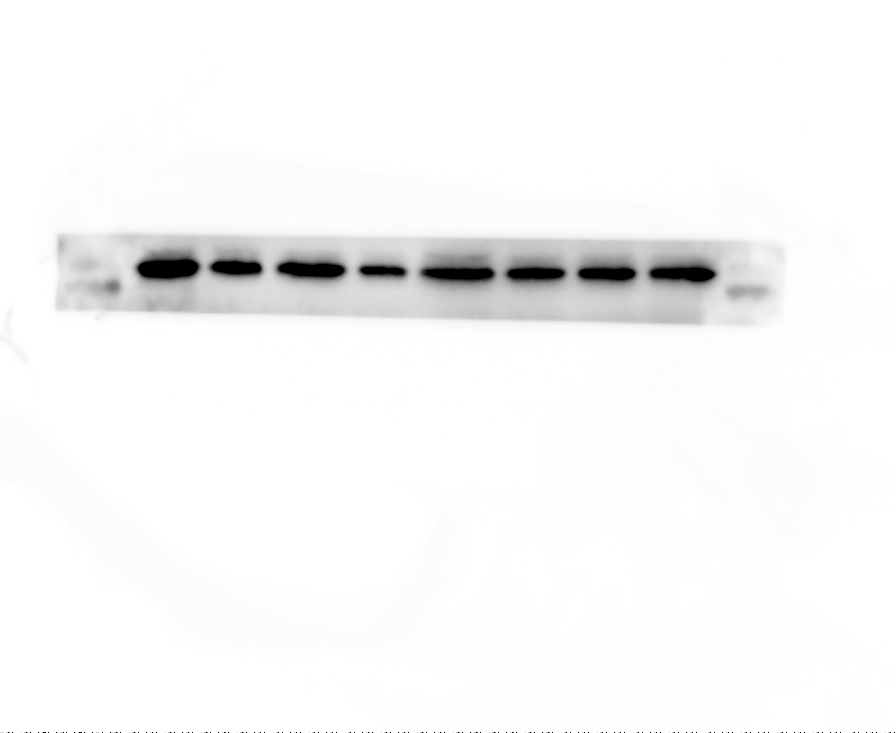

Supplement: Supplementary file 6 — western blots [file 41419_2023_6016_MOESM6_ESM.zip › breast cancer WB/Figure 5G/231,468 DHX9 1-4.tif]

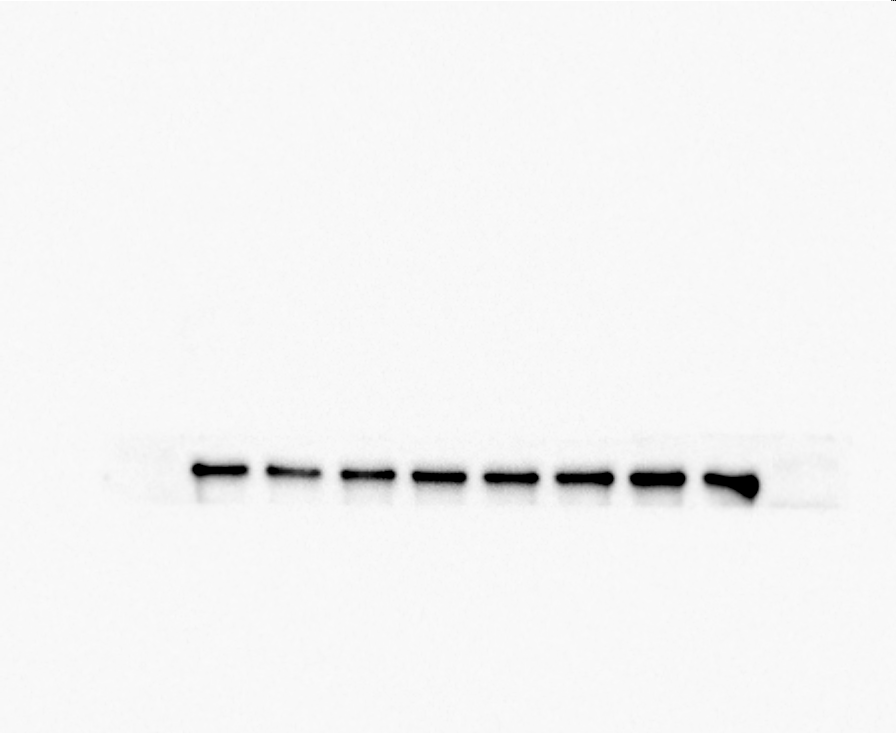

Supplement: Supplementary file 6 — western blots [file 41419_2023_6016_MOESM6_ESM.zip › breast cancer WB/Figure 5G/231,468 tubulin 1-4.tif]

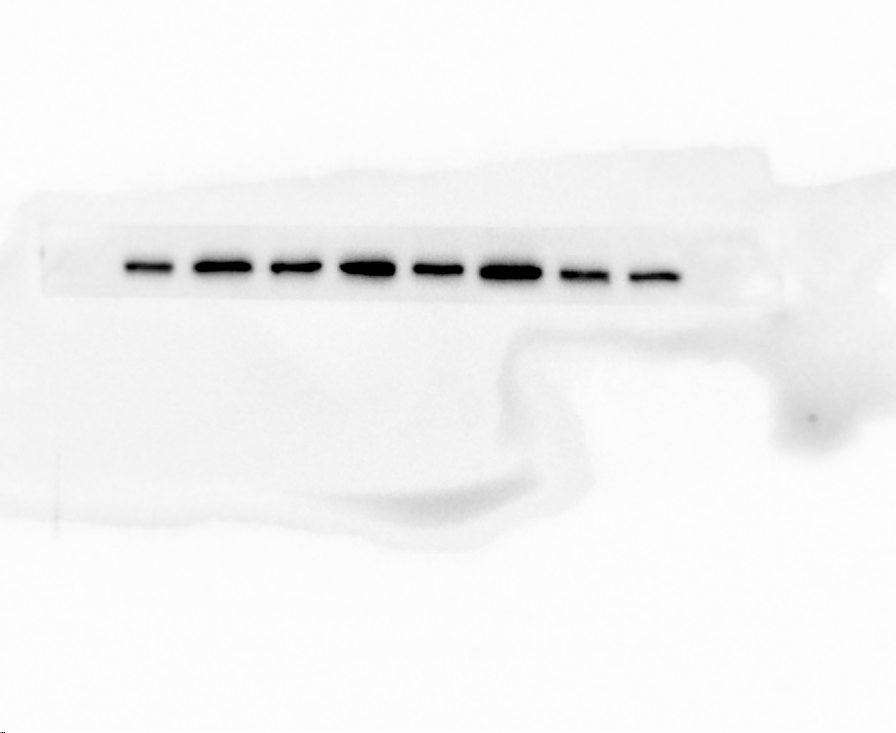

Supplement: Supplementary file 6 — western blots [file 41419_2023_6016_MOESM6_ESM.zip › breast cancer WB/Figure 5G/231,468 RFFL 1-4.tif]

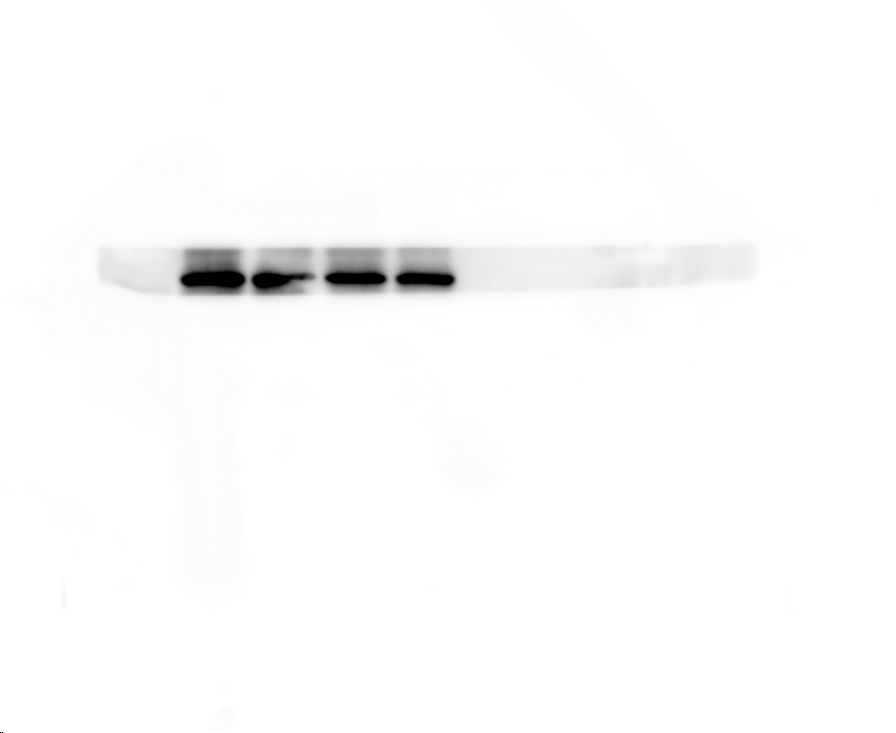

Supplement: Supplementary file 6 — western blots [file 41419_2023_6016_MOESM6_ESM.zip › breast cancer WB/Figure 5H/DHX9.tif]

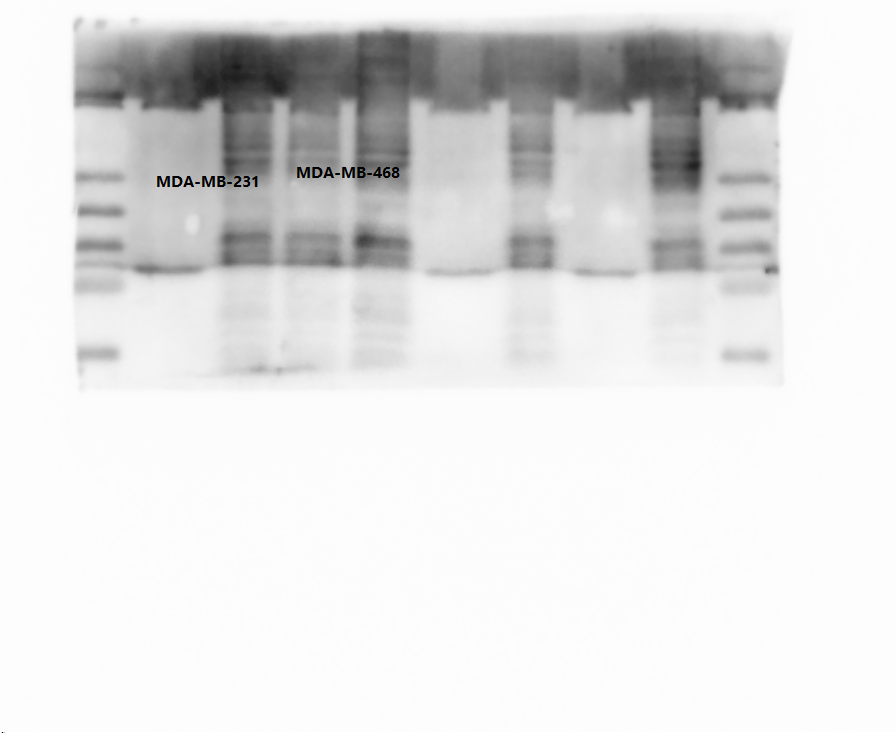

Supplement: Supplementary file 6 — western blots [file 41419_2023_6016_MOESM6_ESM.zip › breast cancer WB/Figure 5H/IP DHX9 HA 231,468cell.tif]

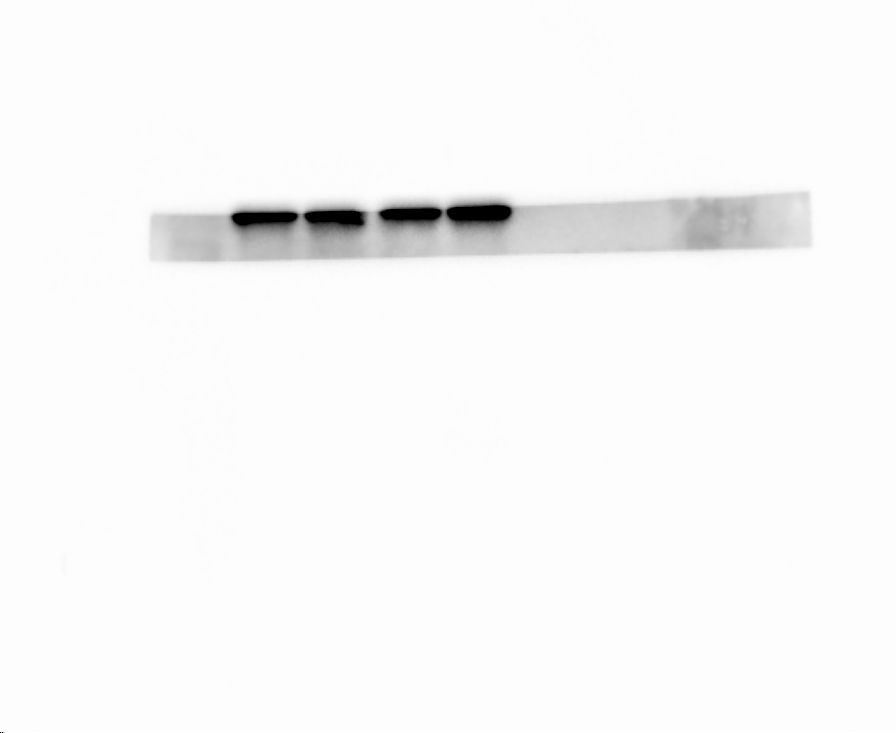

Supplement: Supplementary file 6 — western blots [file 41419_2023_6016_MOESM6_ESM.zip › breast cancer WB/Figure 5H/input RFFL.tif]

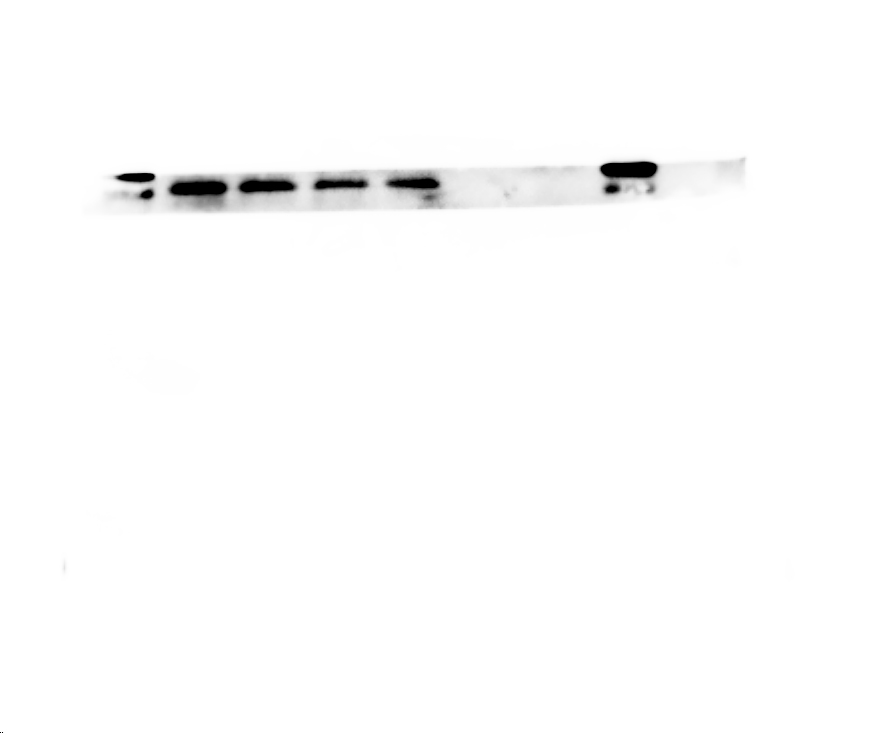

Supplement: Supplementary file 6 — western blots [file 41419_2023_6016_MOESM6_ESM.zip › breast cancer WB/Figure 5H/input tubulin.jpg]

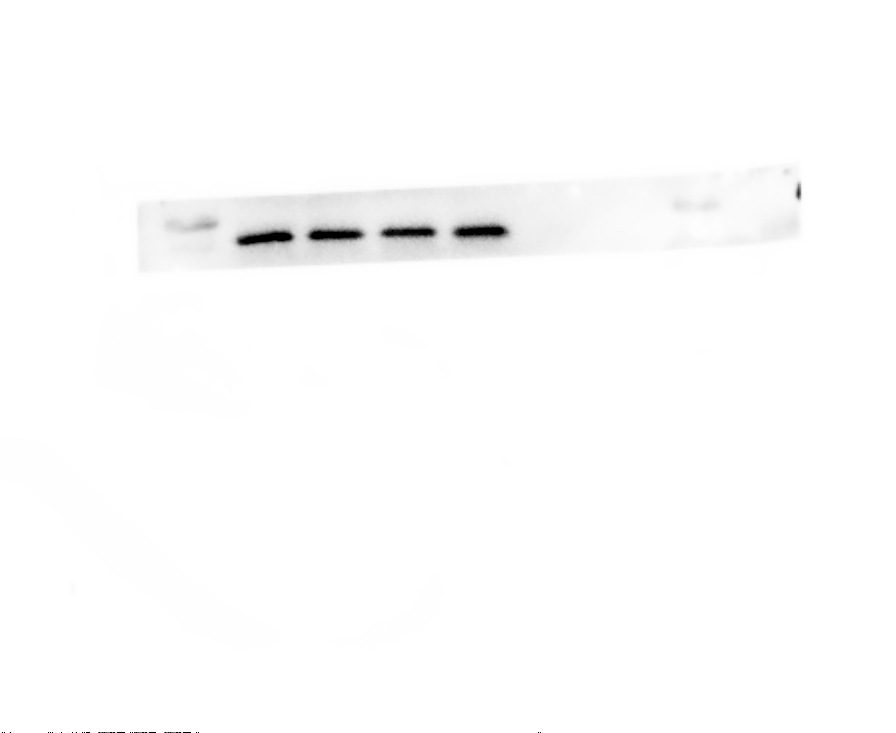

Supplement: Supplementary file 6 — western blots [file 41419_2023_6016_MOESM6_ESM.zip › breast cancer WB/Figure 5H/input DHX9.jpg]

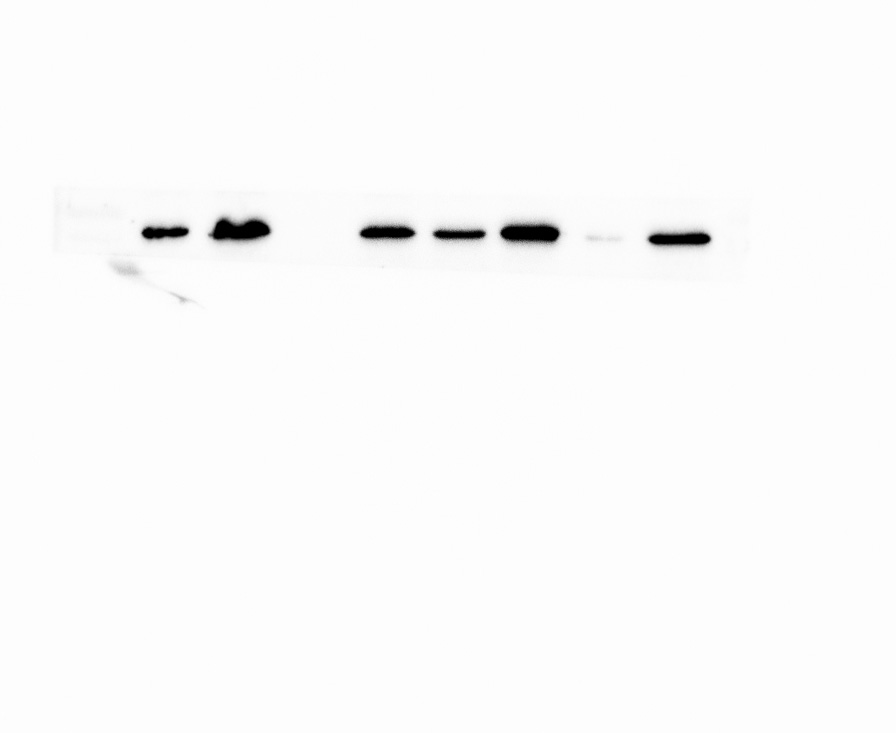

Supplement: Supplementary file 6 — western blots [file 41419_2023_6016_MOESM6_ESM.zip › breast cancer WB/Figure 5I/DHX9 231cell(1-4),468cell(5-8).jpg]

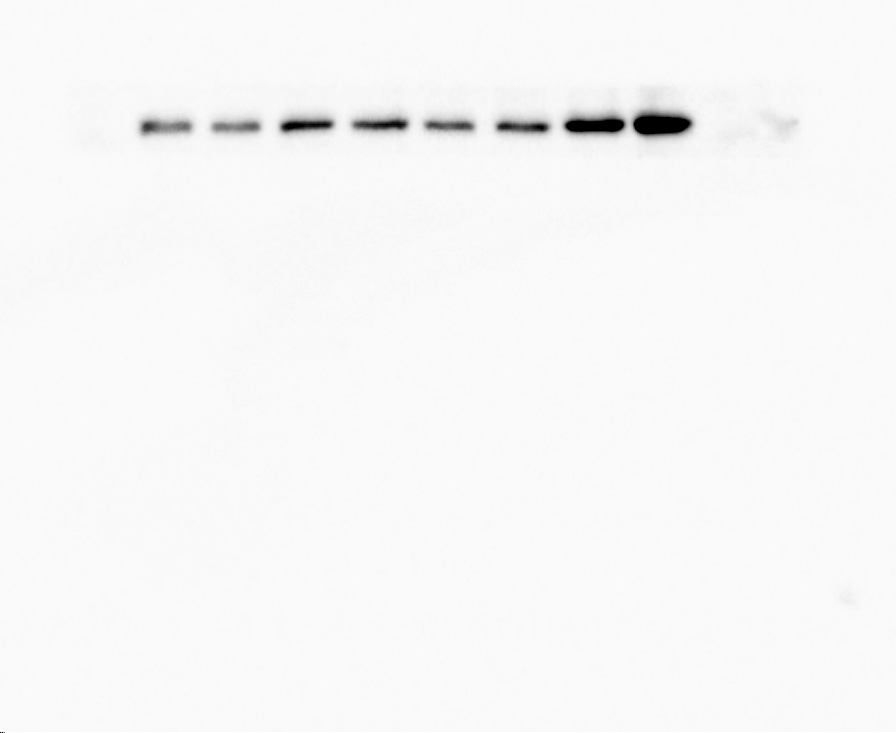

Supplement: Supplementary file 6 — western blots [file 41419_2023_6016_MOESM6_ESM.zip › breast cancer WB/Figure 5I/RFFL 231cell(1-4),468cell(5-8).jpg]

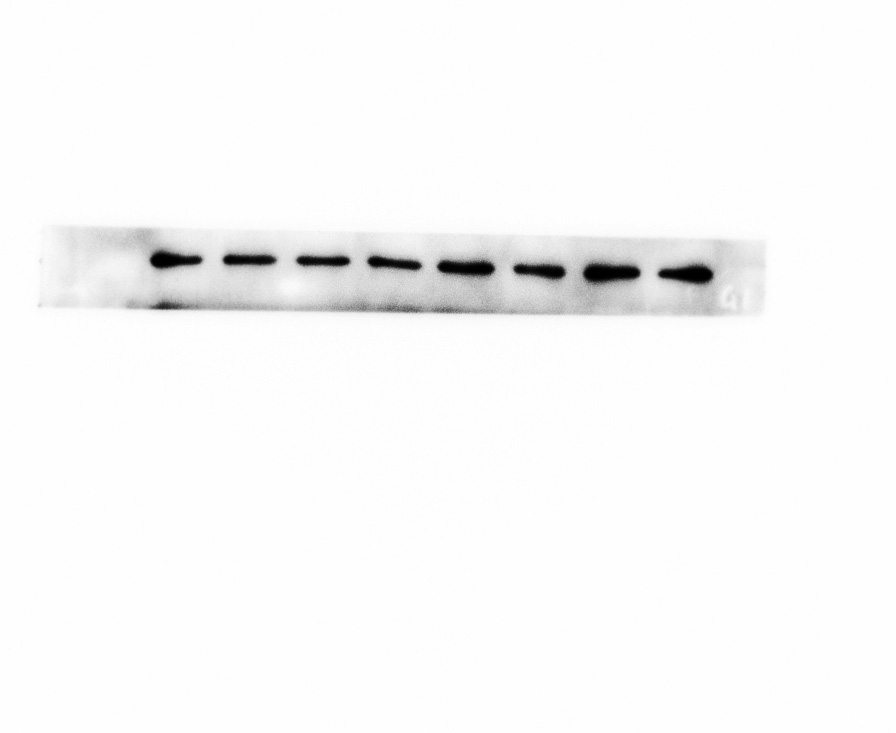

Supplement: Supplementary file 6 — western blots [file 41419_2023_6016_MOESM6_ESM.zip › breast cancer WB/Figure 5I/Tubulin 231cell(1-4),468cell(5-8).jpg]

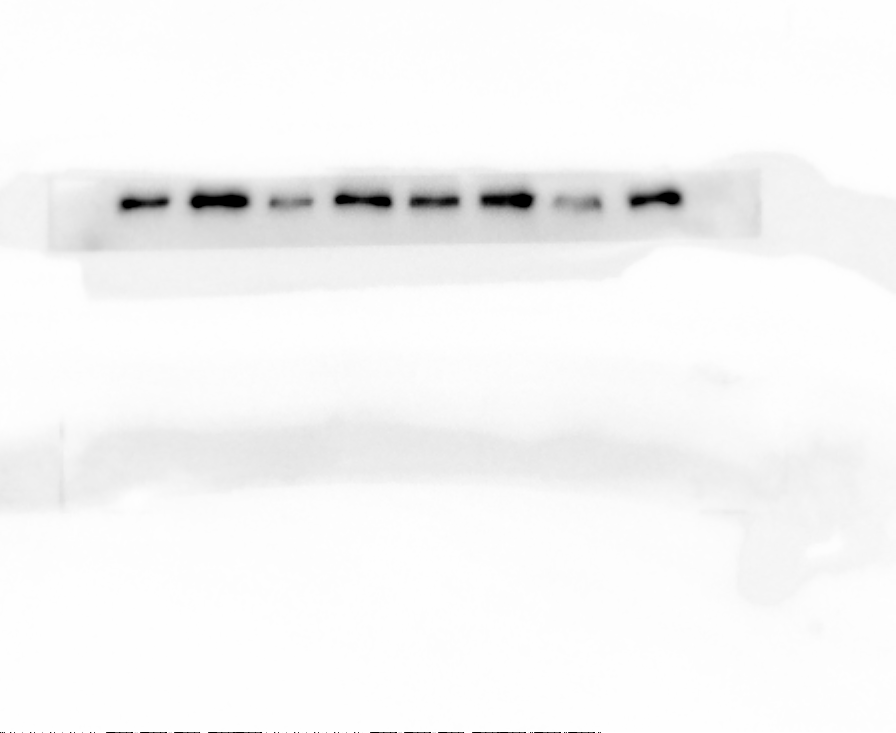

Supplement: Supplementary file 6 — western blots [file 41419_2023_6016_MOESM6_ESM.zip › breast cancer WB/Figure 5J/DHX9 231cell (1-6).tif]

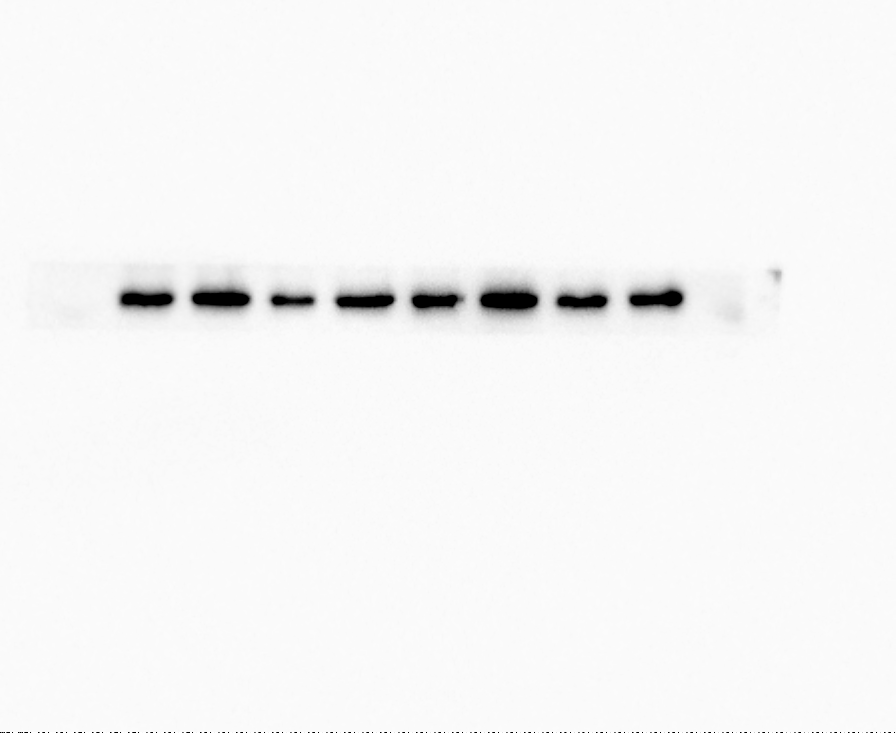

Supplement: Supplementary file 6 — western blots [file 41419_2023_6016_MOESM6_ESM.zip › breast cancer WB/Figure 5J/DHX9 468cell (1-6).tif]

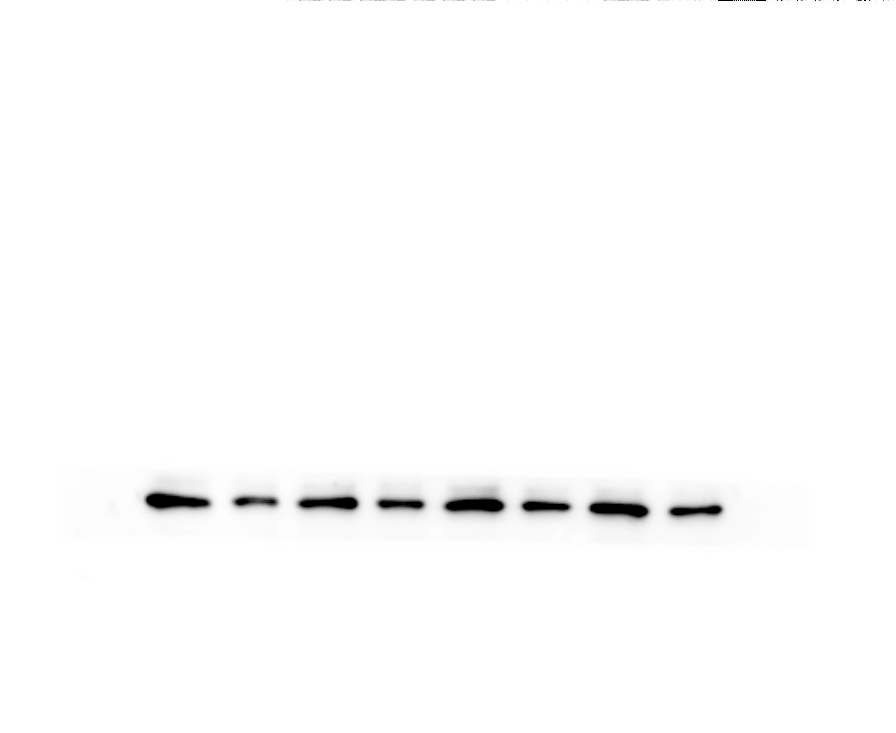

Supplement: Supplementary file 6 — western blots [file 41419_2023_6016_MOESM6_ESM.zip › breast cancer WB/Figure 5J/RFFL 231cell (1-6).tiff]

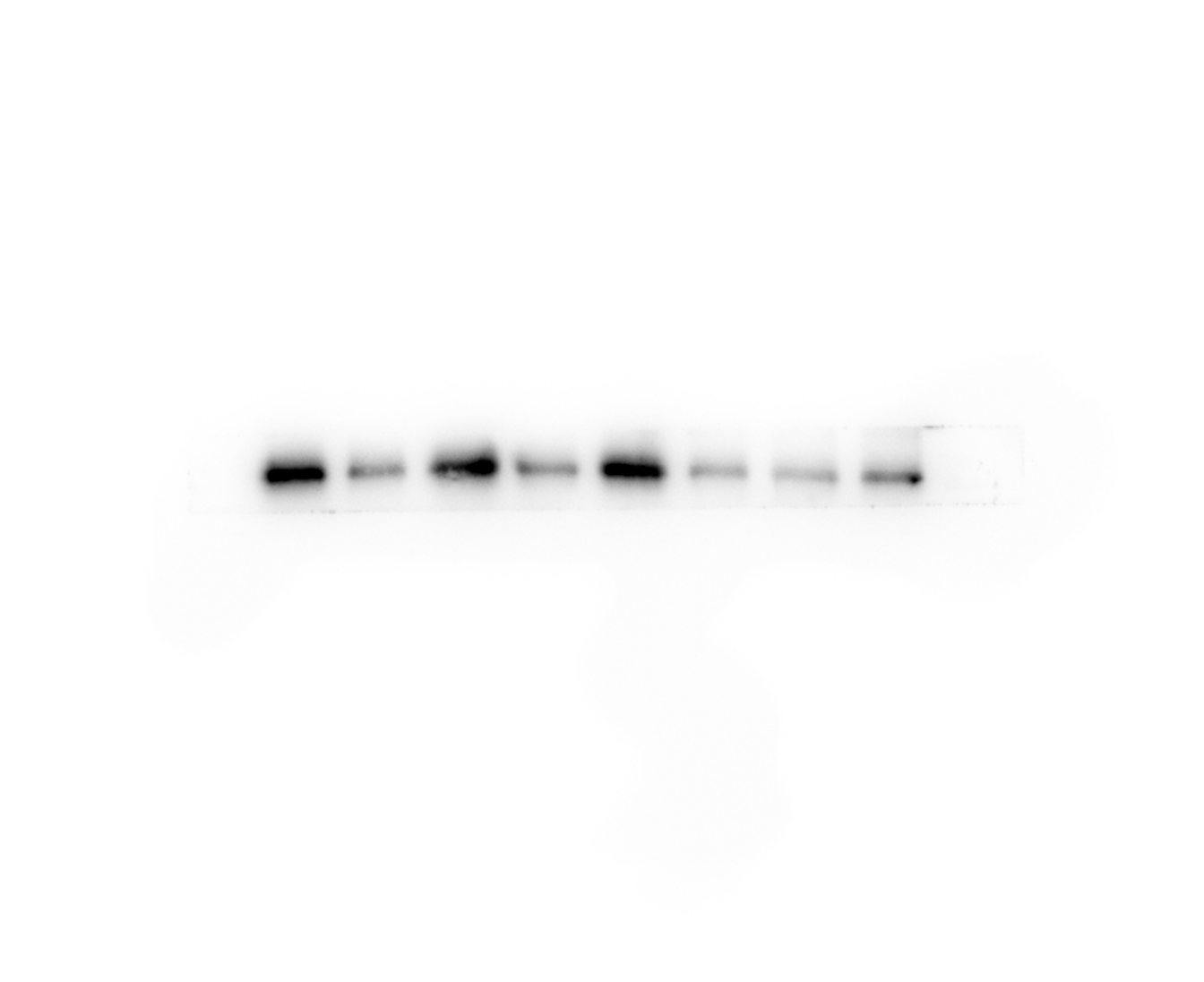

Supplement: Supplementary file 6 — western blots [file 41419_2023_6016_MOESM6_ESM.zip › breast cancer WB/Figure 5J/RFFL 468cell (1-6).Tif]

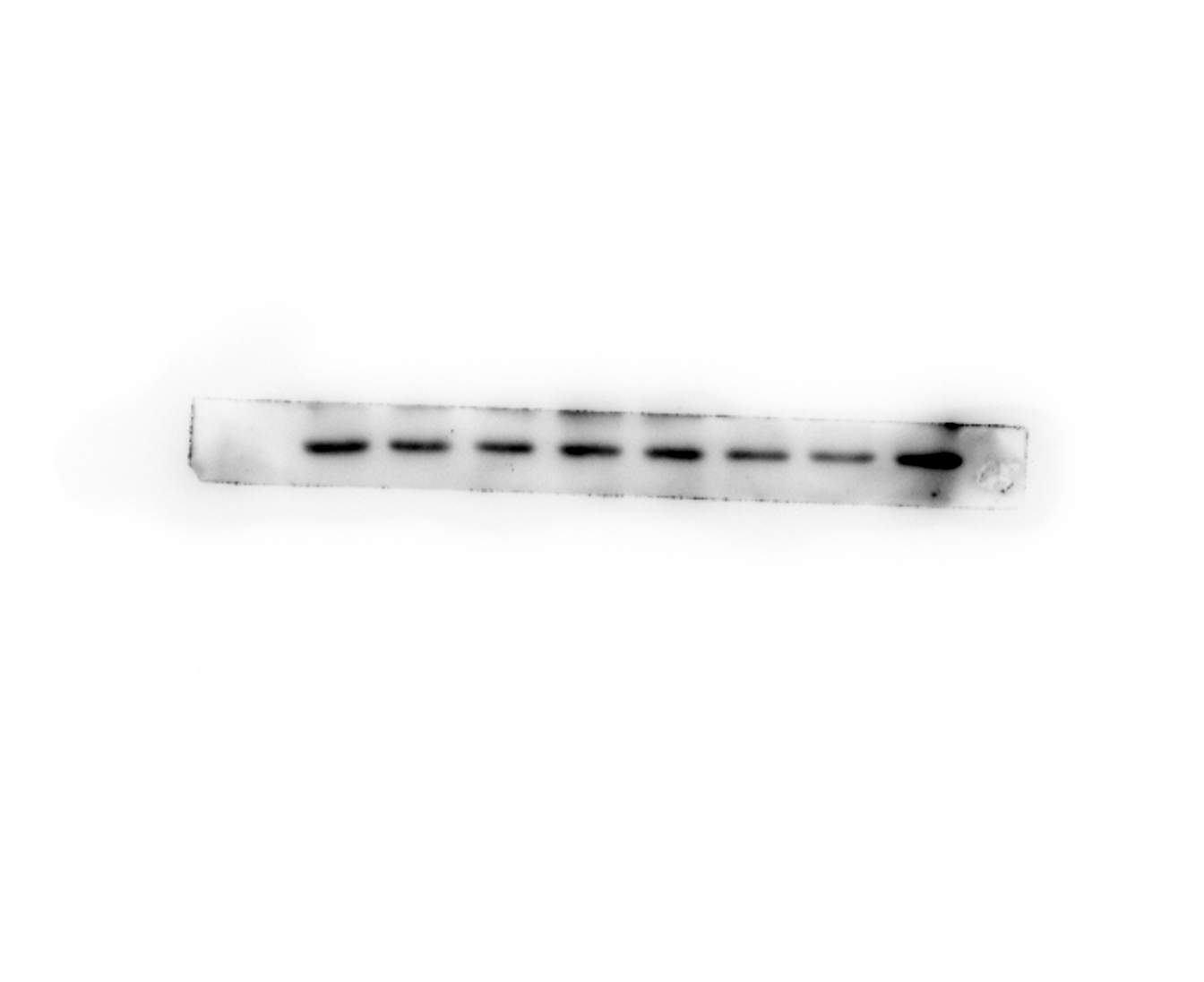

Supplement: Supplementary file 6 — western blots [file 41419_2023_6016_MOESM6_ESM.zip › breast cancer WB/Figure 5J/Tubulin 231cell (1-6).Tif]

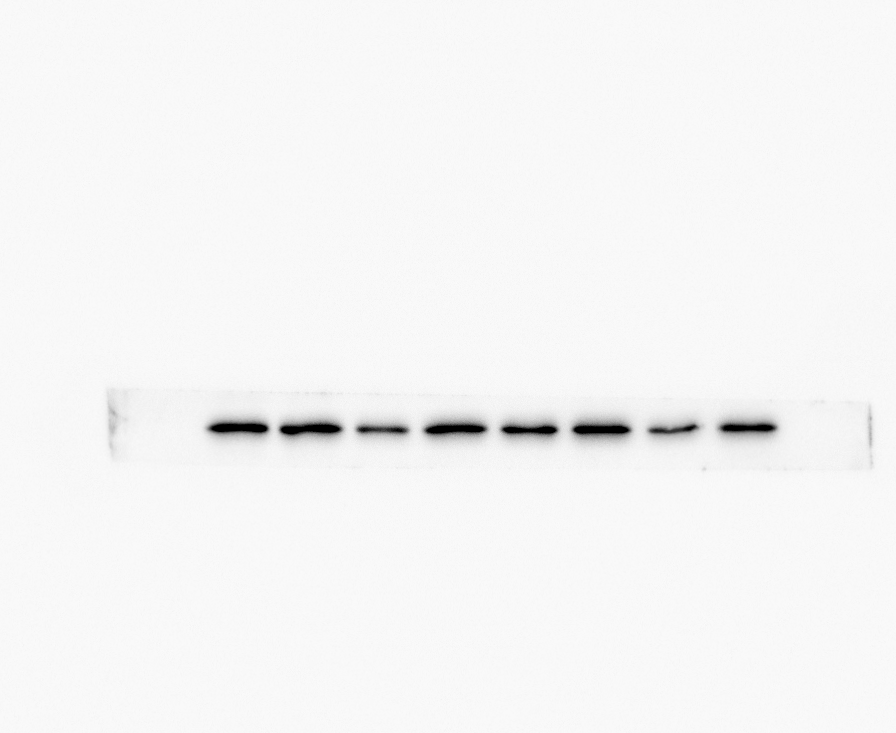

Supplement: Supplementary file 6 — western blots [file 41419_2023_6016_MOESM6_ESM.zip › breast cancer WB/Figure 5J/Tubulin 468cell (1-6).tif]

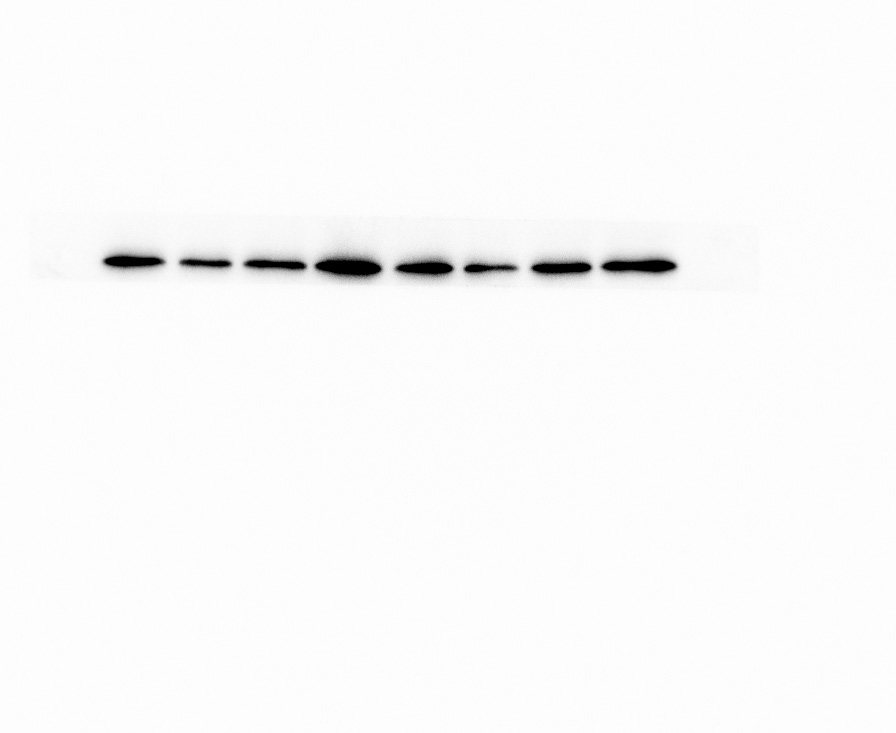

Supplement: Supplementary file 6 — western blots [file 41419_2023_6016_MOESM6_ESM.zip › breast cancer WB/Figure 5K/DHX9 231cell (1-4),468 cell(5-8).jpg]

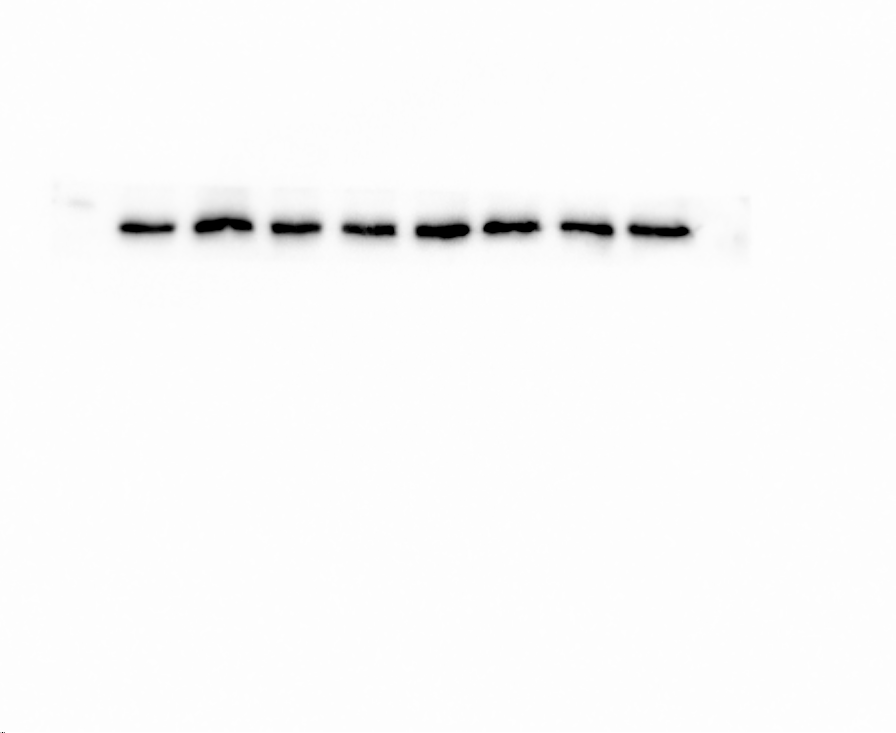

Supplement: Supplementary file 6 — western blots [file 41419_2023_6016_MOESM6_ESM.zip › breast cancer WB/Figure 5K/RFFL 231cell (1-4),468 cell(5-8).tif]

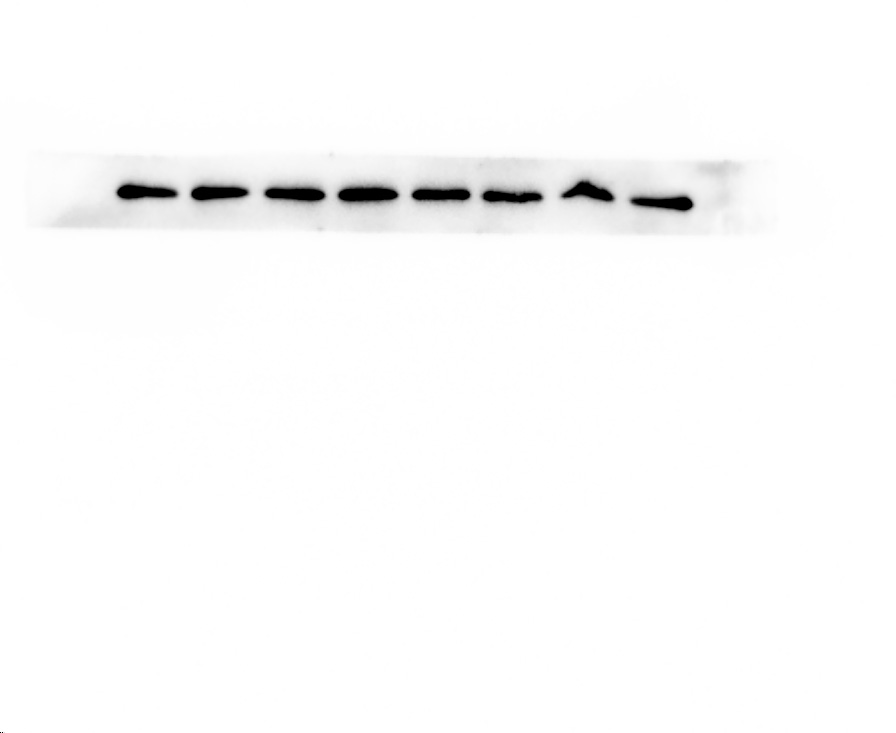

Supplement: Supplementary file 6 — western blots [file 41419_2023_6016_MOESM6_ESM.zip › breast cancer WB/Figure 5K/input DHX9 231cell (1-4),468 cell(5-8).jpg]

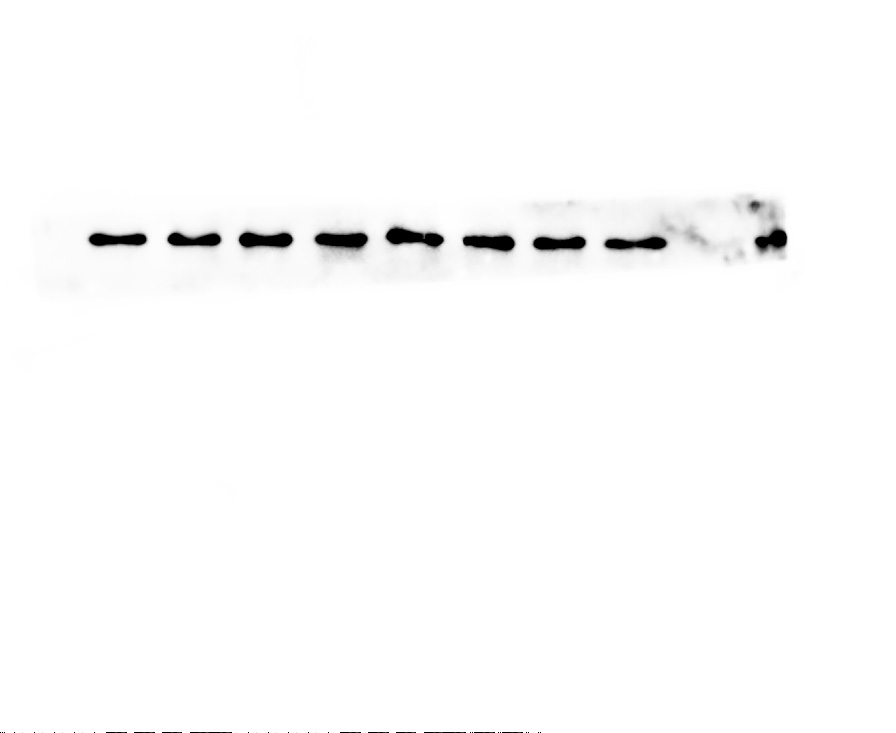

Supplement: Supplementary file 6 — western blots [file 41419_2023_6016_MOESM6_ESM.zip › breast cancer WB/Figure 5K/input RFFL 231cell (1-4),468 cell(5-8).jpg]

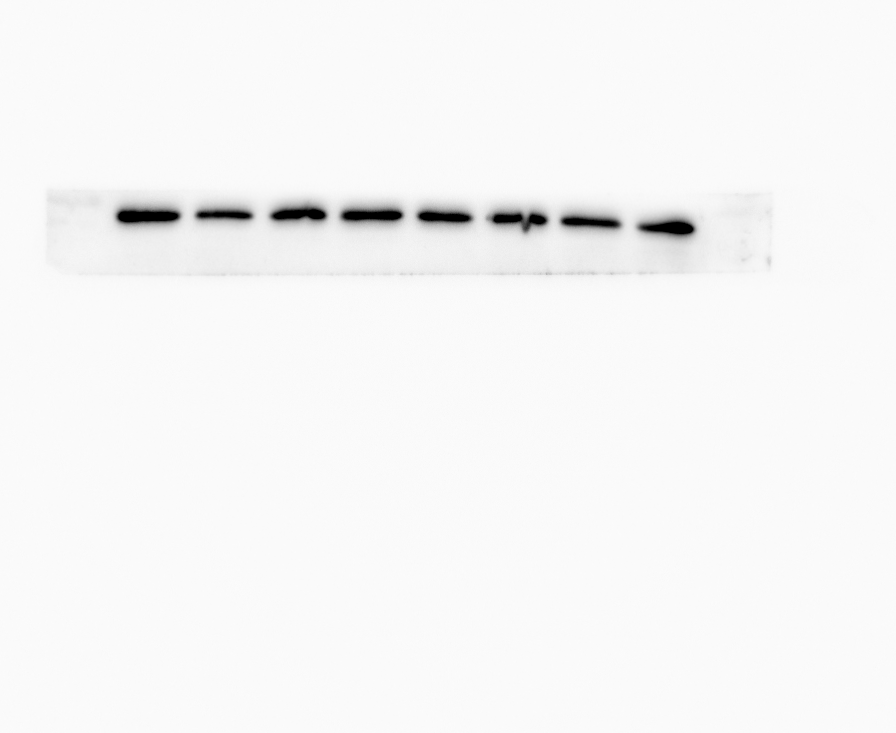

Supplement: Supplementary file 6 — western blots [file 41419_2023_6016_MOESM6_ESM.zip › breast cancer WB/Figure 5K/input Tubulin,231cell (1-4),468 cell(5-8).tif]

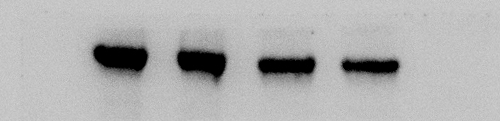

Supplement: Supplementary file 6 — western blots [file 41419_2023_6016_MOESM6_ESM.zip › breast cancer WB/Figure 5L/DHX9-231.jpg]

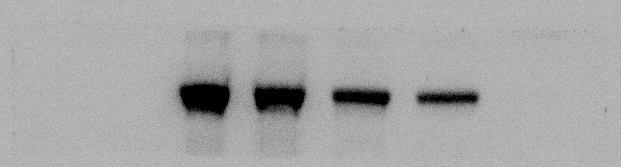

Supplement: Supplementary file 6 — western blots [file 41419_2023_6016_MOESM6_ESM.zip › breast cancer WB/Figure 5L/DHX9-468.jpg]

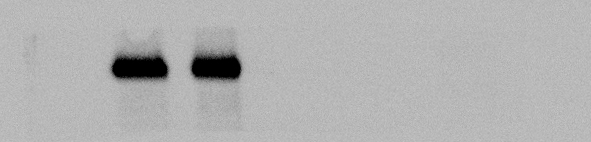

Supplement: Supplementary file 6 — western blots [file 41419_2023_6016_MOESM6_ESM.zip › breast cancer WB/Figure 5L/GAPDH-231.jpg]

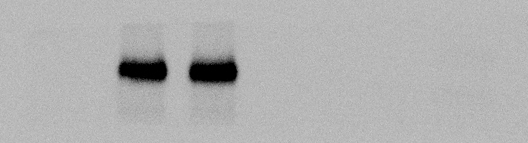

Supplement: Supplementary file 6 — western blots [file 41419_2023_6016_MOESM6_ESM.zip › breast cancer WB/Figure 5L/GAPDH-468.jpg]

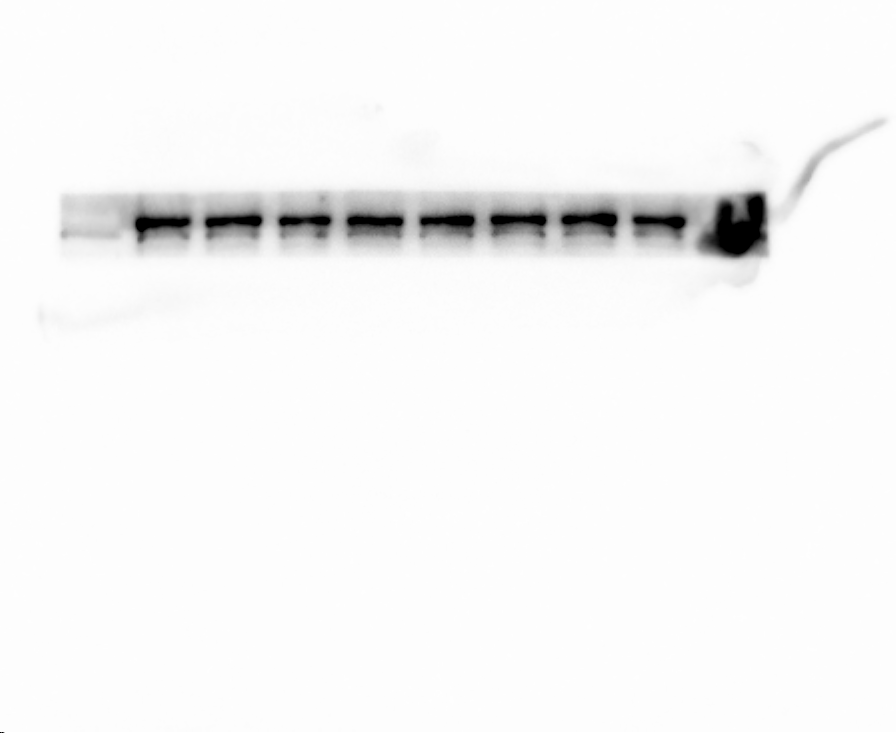

Supplement: Supplementary file 6 — western blots [file 41419_2023_6016_MOESM6_ESM.zip › breast cancer WB/Figure 5M/AKT 231cell (1-2);468cell(3-4).tif]

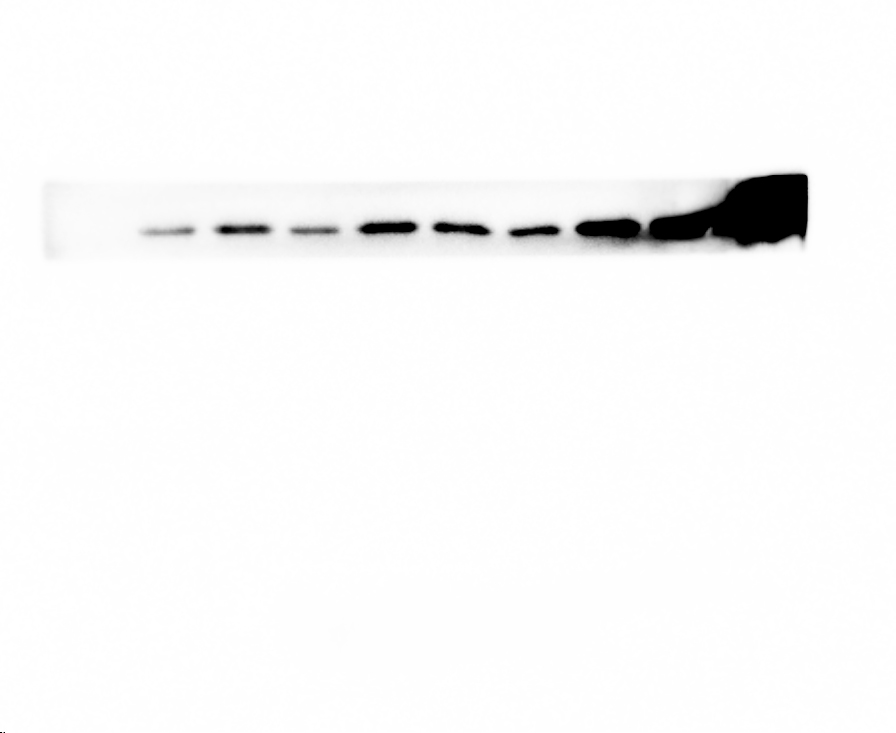

Supplement: Supplementary file 6 — western blots [file 41419_2023_6016_MOESM6_ESM.zip › breast cancer WB/Figure 5M/DHX9 231cell (1-2);468cell(3-4).jpg]

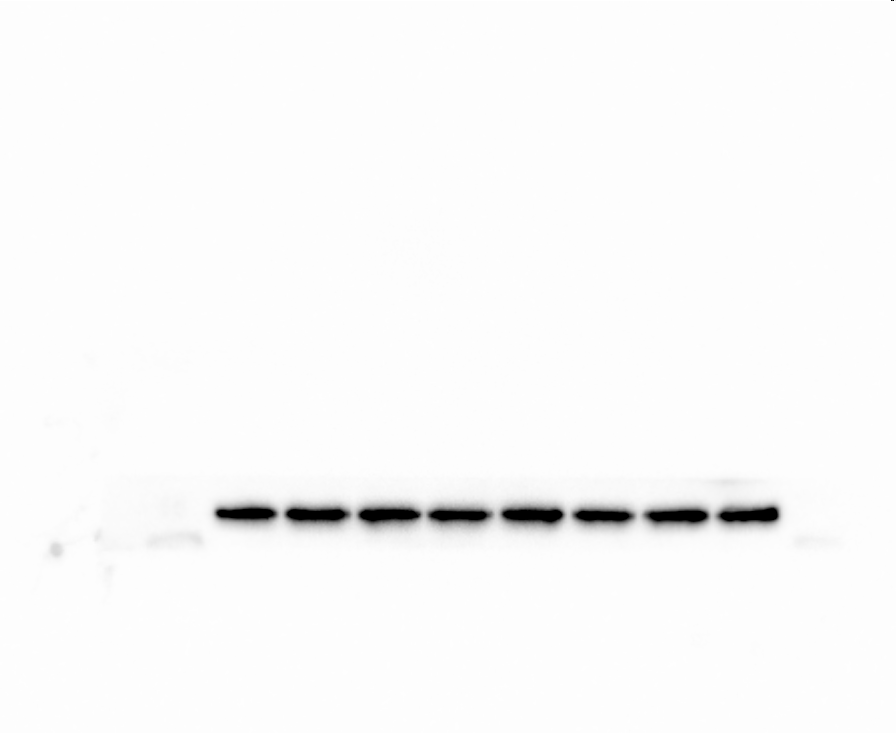

Supplement: Supplementary file 6 — western blots [file 41419_2023_6016_MOESM6_ESM.zip › breast cancer WB/Figure 5M/GADPH231cell (1-2);468cell(3-4).tif]

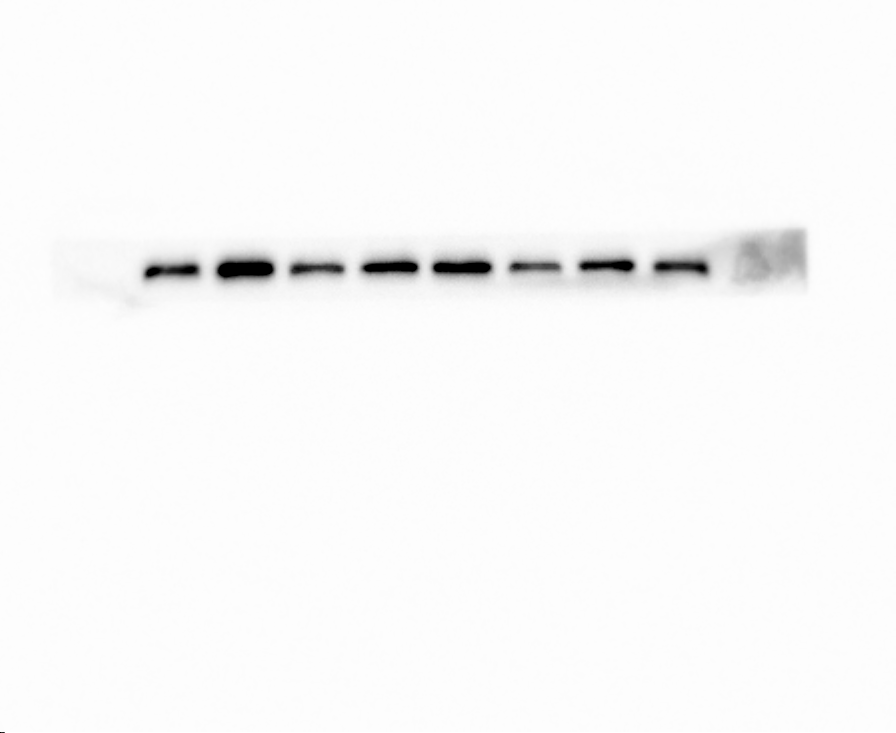

Supplement: Supplementary file 6 — western blots [file 41419_2023_6016_MOESM6_ESM.zip › breast cancer WB/Figure 5M/MMP9 231cell (1-2);468cell(3-4).tif]

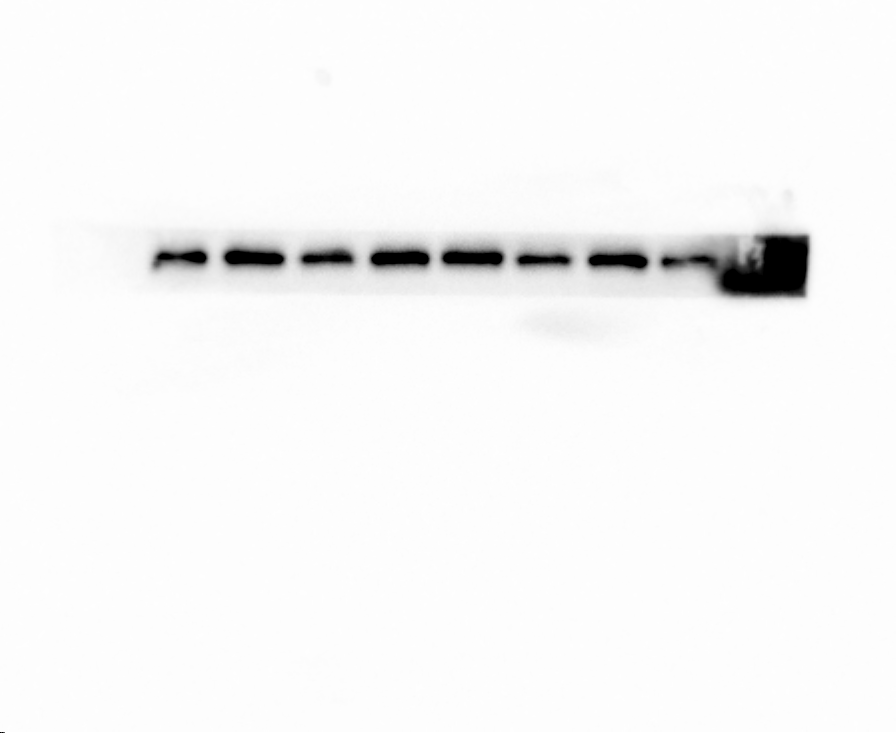

Supplement: Supplementary file 6 — western blots [file 41419_2023_6016_MOESM6_ESM.zip › breast cancer WB/Figure 5M/bcl-2 231cell (1-2);468cell(3-4).tif]

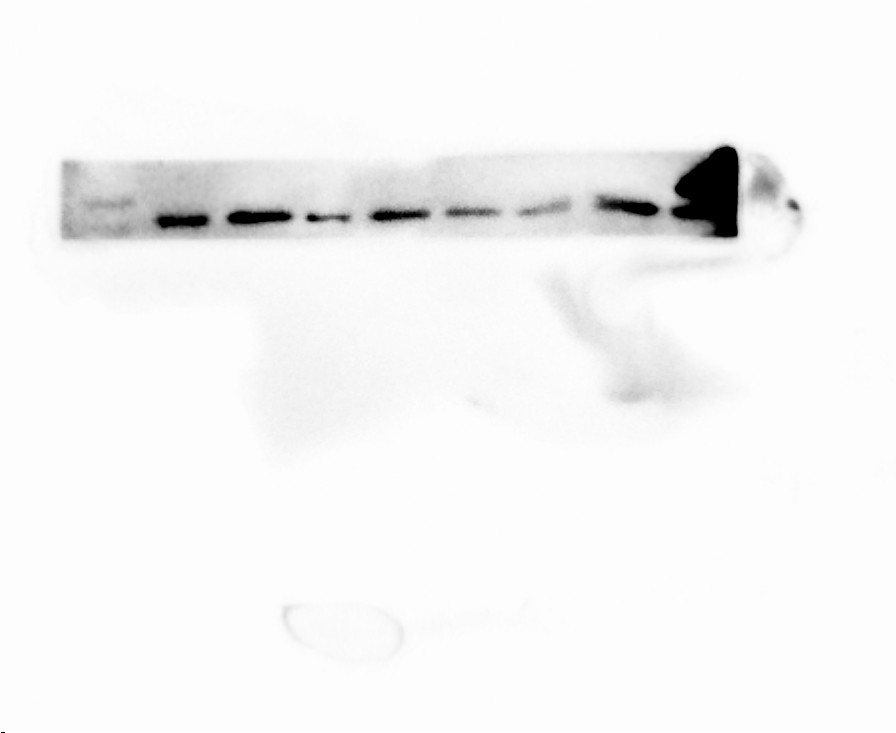

Supplement: Supplementary file 6 — western blots [file 41419_2023_6016_MOESM6_ESM.zip › breast cancer WB/Figure 5M/p-AKT 231cell (1-2);468cell(3-4).jpg]

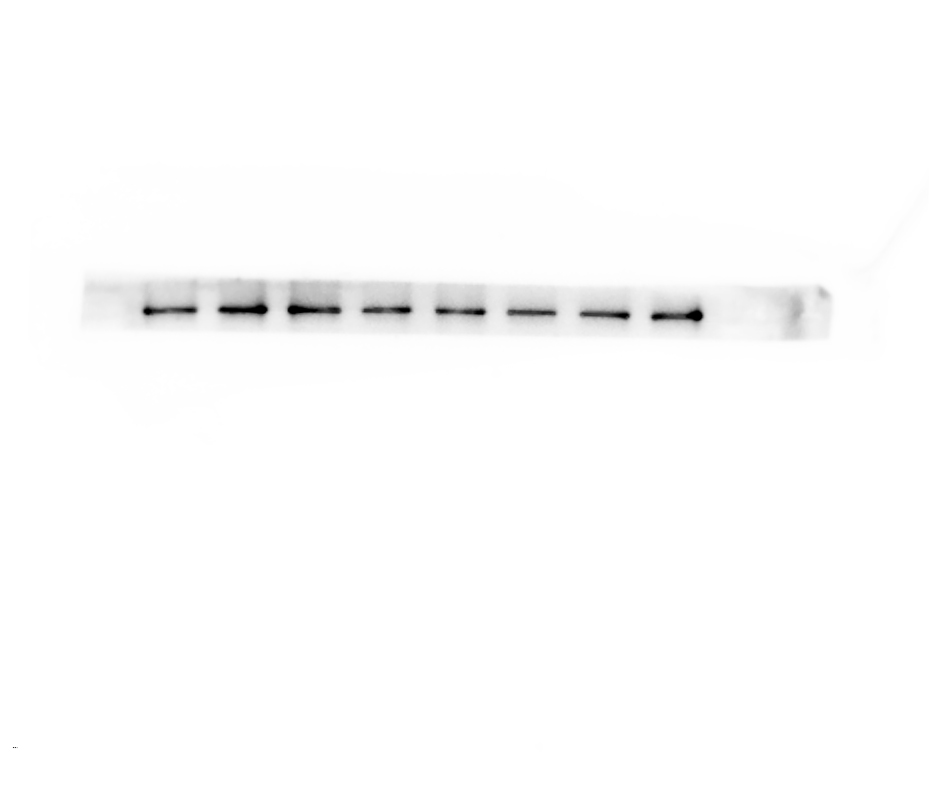

Supplement: Supplementary file 6 — western blots [file 41419_2023_6016_MOESM6_ESM.zip › breast cancer WB/Figure 5N/231cell/AKT 231cell (1-4).jpg]

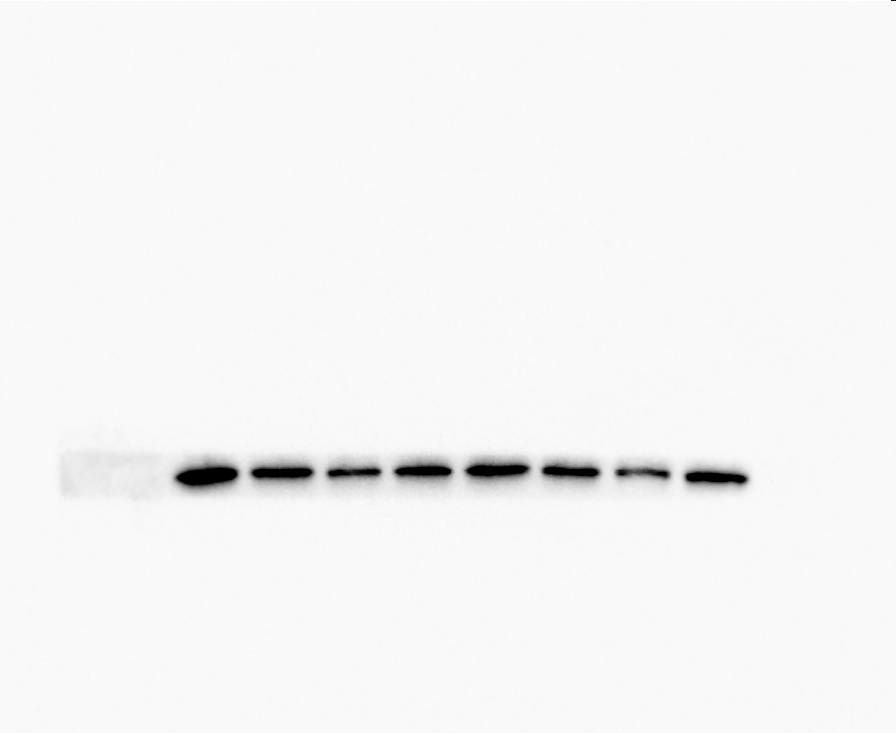

Supplement: Supplementary file 6 — western blots [file 41419_2023_6016_MOESM6_ESM.zip › breast cancer WB/Figure 5N/231cell/DHX9 231cell (1-4).tif]

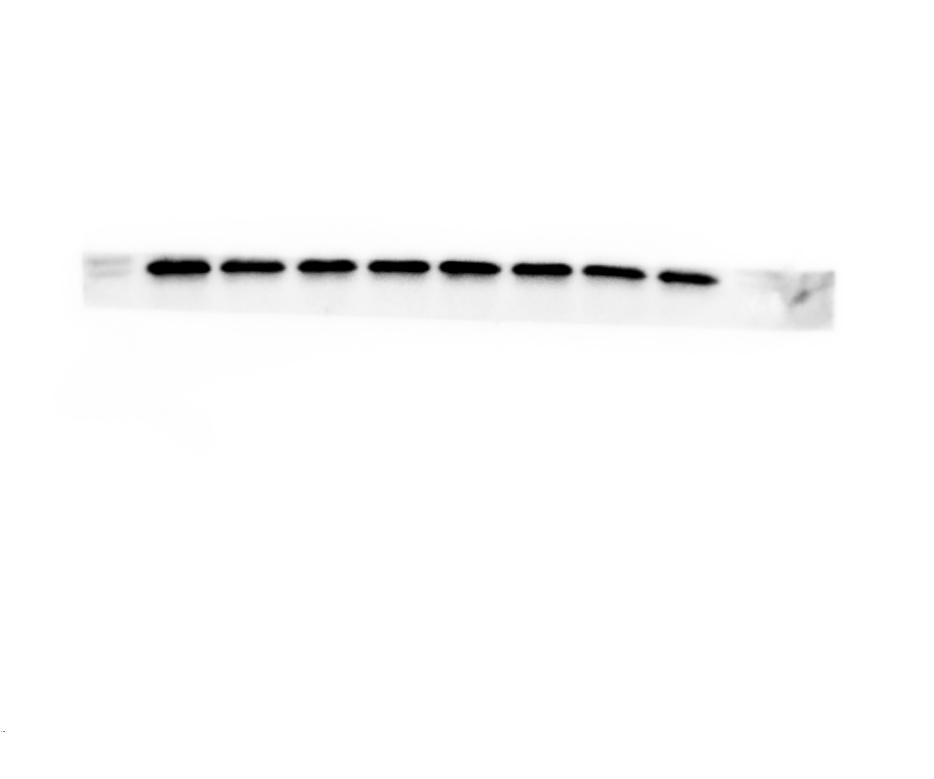

Supplement: Supplementary file 6 — western blots [file 41419_2023_6016_MOESM6_ESM.zip › breast cancer WB/Figure 5N/231cell/GADPH 231cell (1-4).jpg]

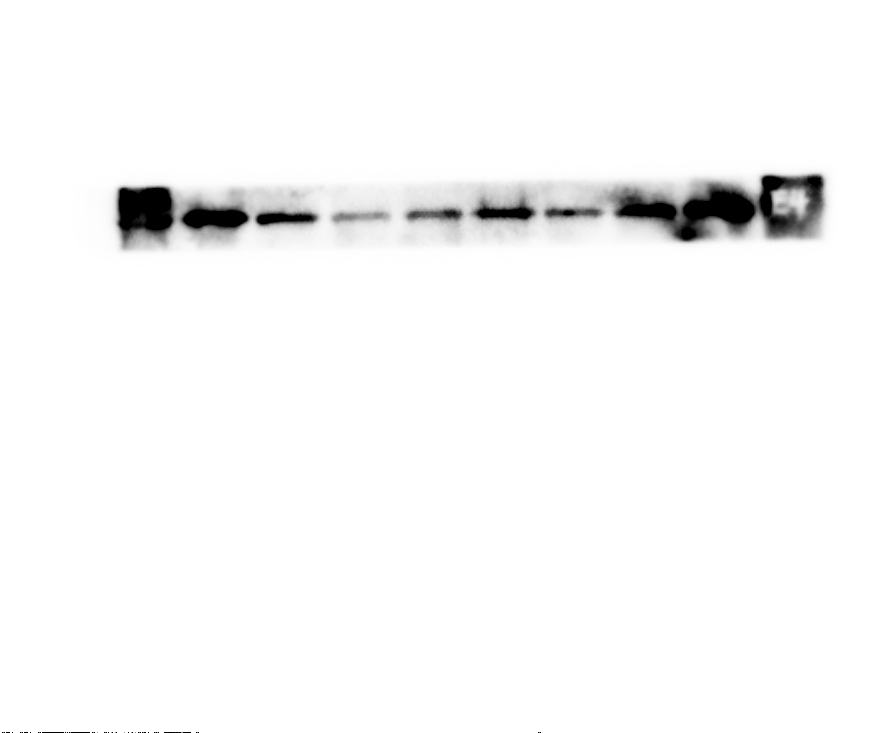

Supplement: Supplementary file 6 — western blots [file 41419_2023_6016_MOESM6_ESM.zip › breast cancer WB/Figure 5N/231cell/MMP9 231cell (1-4).jpg]

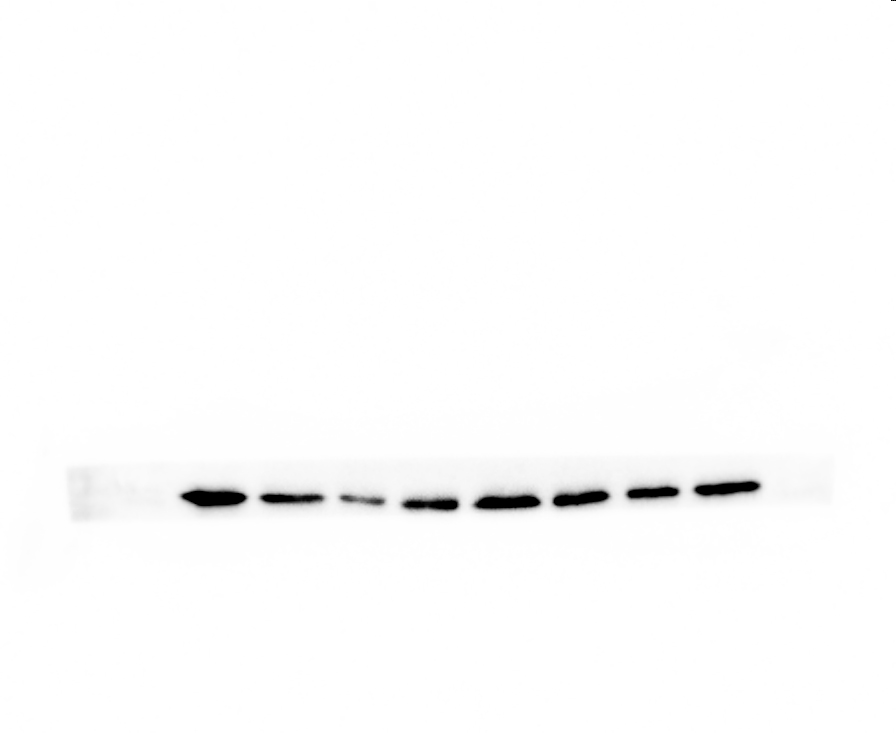

Supplement: Supplementary file 6 — western blots [file 41419_2023_6016_MOESM6_ESM.zip › breast cancer WB/Figure 5N/231cell/bcl-2 231cell (1-4).jpg]

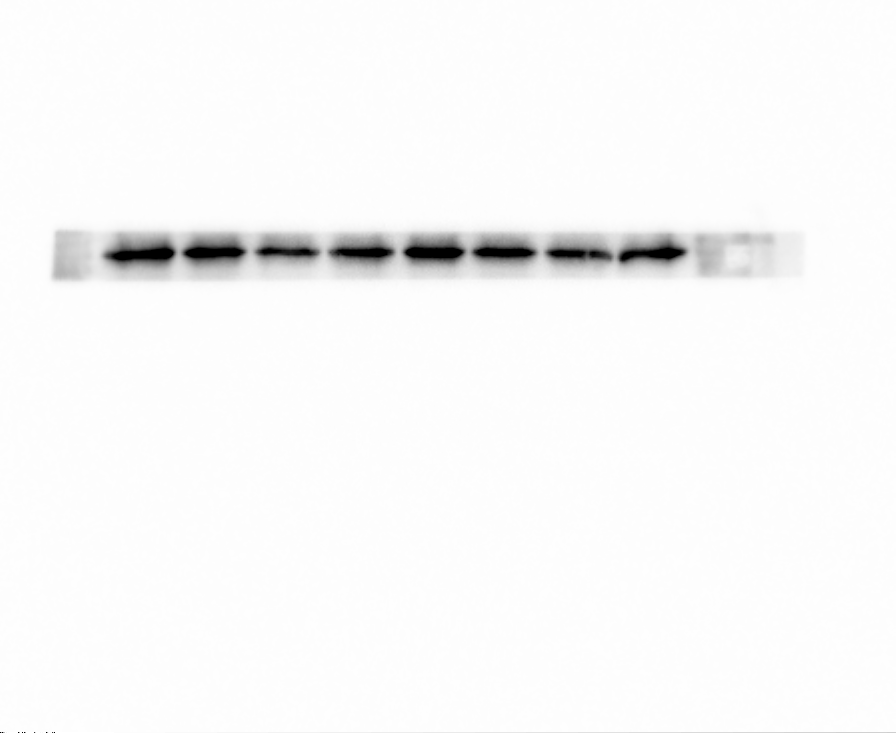

Supplement: Supplementary file 6 — western blots [file 41419_2023_6016_MOESM6_ESM.zip › breast cancer WB/Figure 5N/231cell/p-AKT 231cell (1-4).tif]

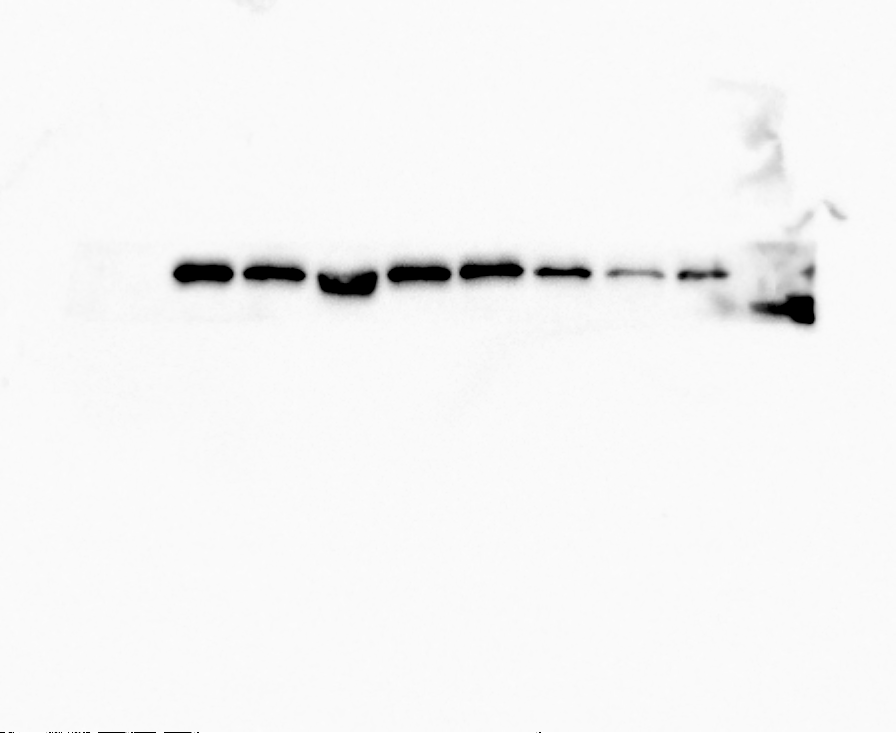

Supplement: Supplementary file 6 — western blots [file 41419_2023_6016_MOESM6_ESM.zip › breast cancer WB/Figure 5N/468 cell/DHX9 468cell (5-8).tif]

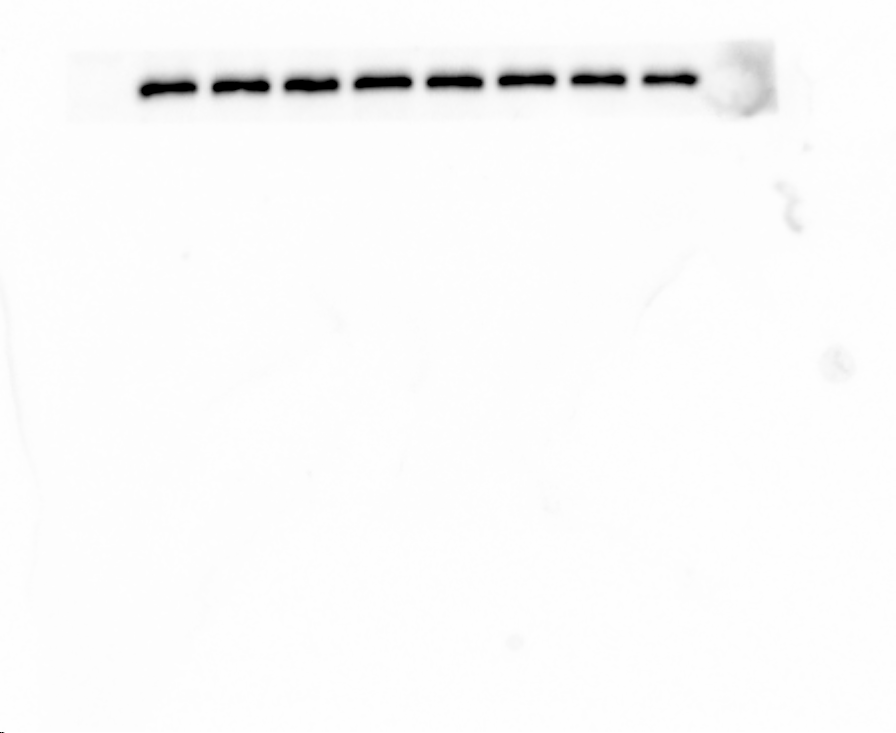

Supplement: Supplementary file 6 — western blots [file 41419_2023_6016_MOESM6_ESM.zip › breast cancer WB/Figure 5N/468 cell/GADPH 468cell (5-8).tif]

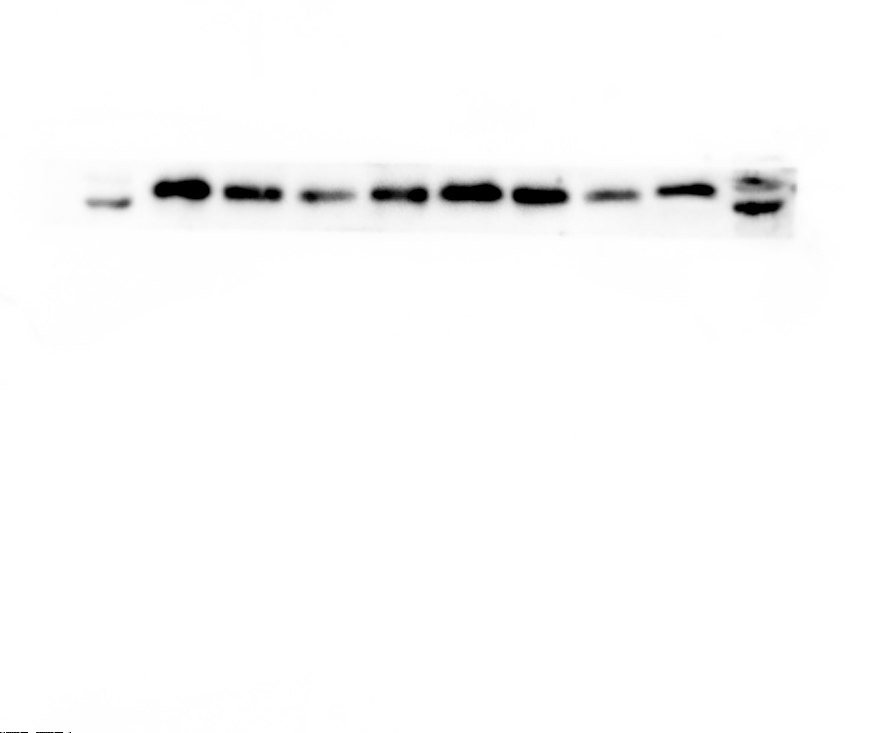

Supplement: Supplementary file 6 — western blots [file 41419_2023_6016_MOESM6_ESM.zip › breast cancer WB/Figure 5N/468 cell/MMP9 468cell (5-8).jpg]

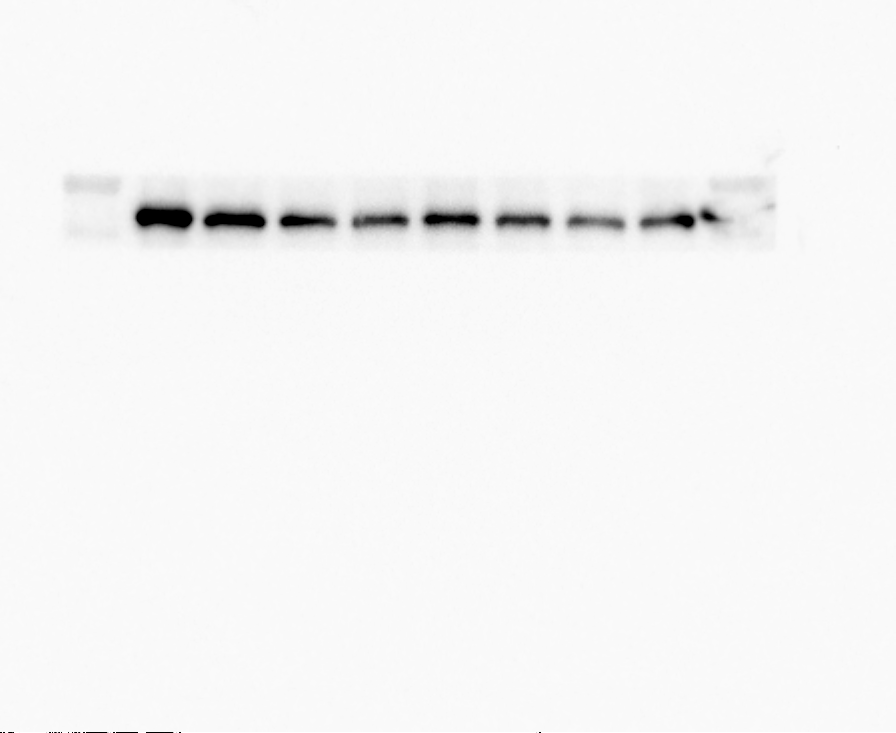

Supplement: Supplementary file 6 — western blots [file 41419_2023_6016_MOESM6_ESM.zip › breast cancer WB/Figure 5N/468 cell/bcl-2 468cell (5-8).tif]

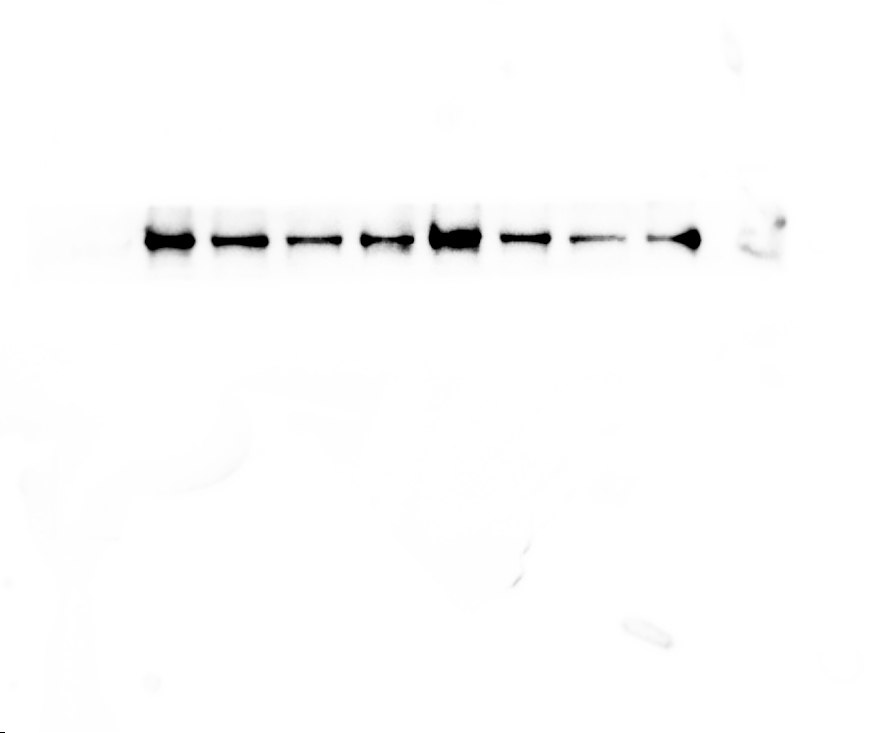

Supplement: Supplementary file 6 — western blots [file 41419_2023_6016_MOESM6_ESM.zip › breast cancer WB/Figure 5N/468 cell/p-AKT 468cell (5-8).jpg]

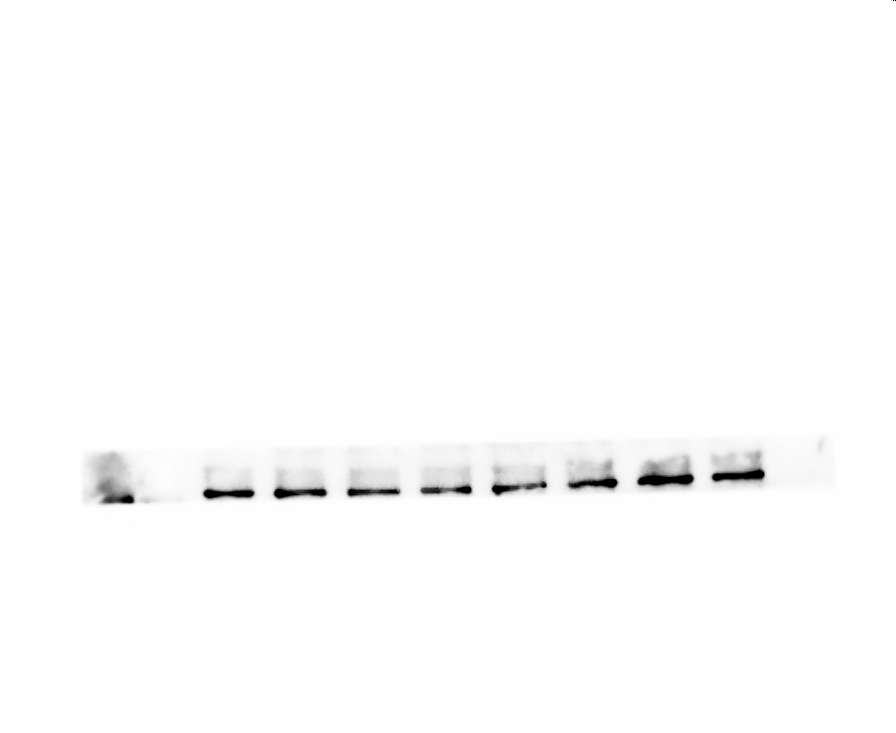

Supplement: Supplementary file 6 — western blots [file 41419_2023_6016_MOESM6_ESM.zip › breast cancer WB/Figure 6J/AKT 1-2.jpg]

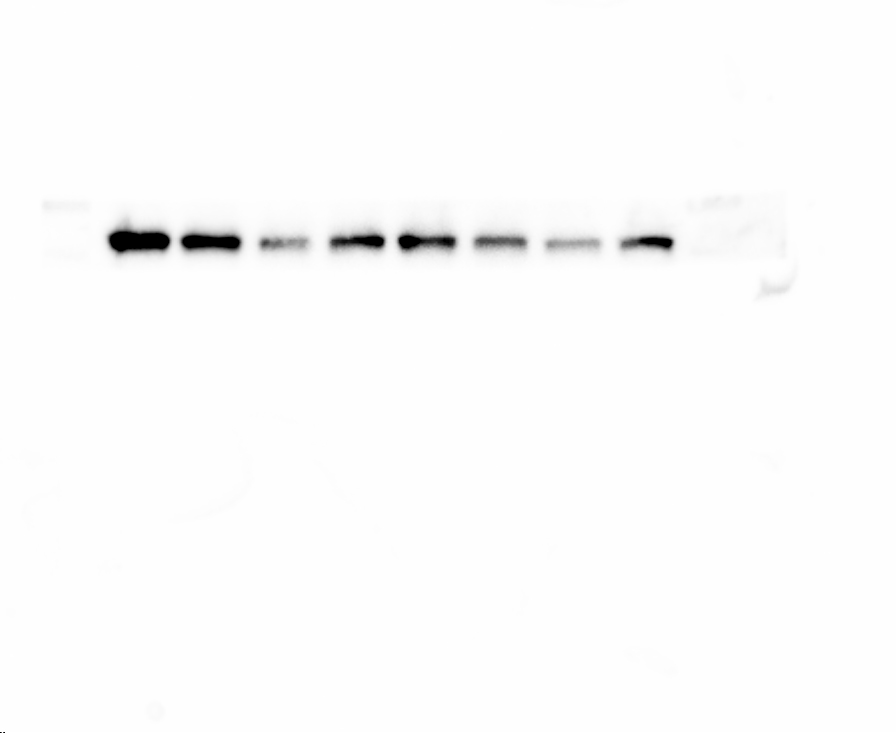

Supplement: Supplementary file 6 — western blots [file 41419_2023_6016_MOESM6_ESM.zip › breast cancer WB/Figure 6J/BCL-2 1-2.jpg]

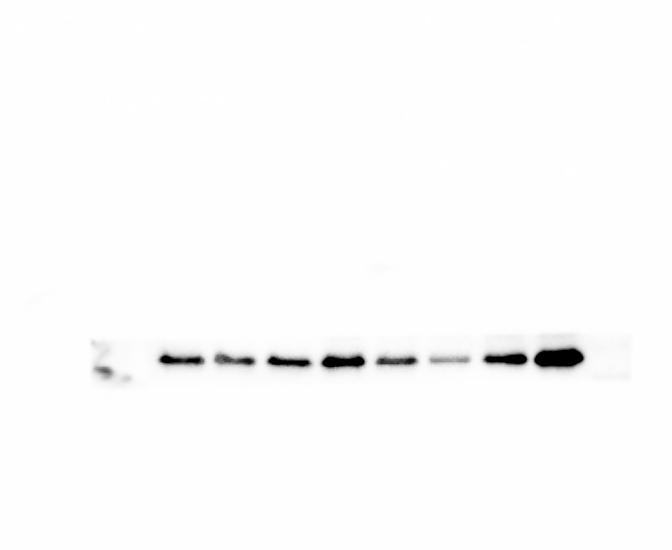

Supplement: Supplementary file 6 — western blots [file 41419_2023_6016_MOESM6_ESM.zip › breast cancer WB/Figure 6J/DHX9 1-2.jpg]

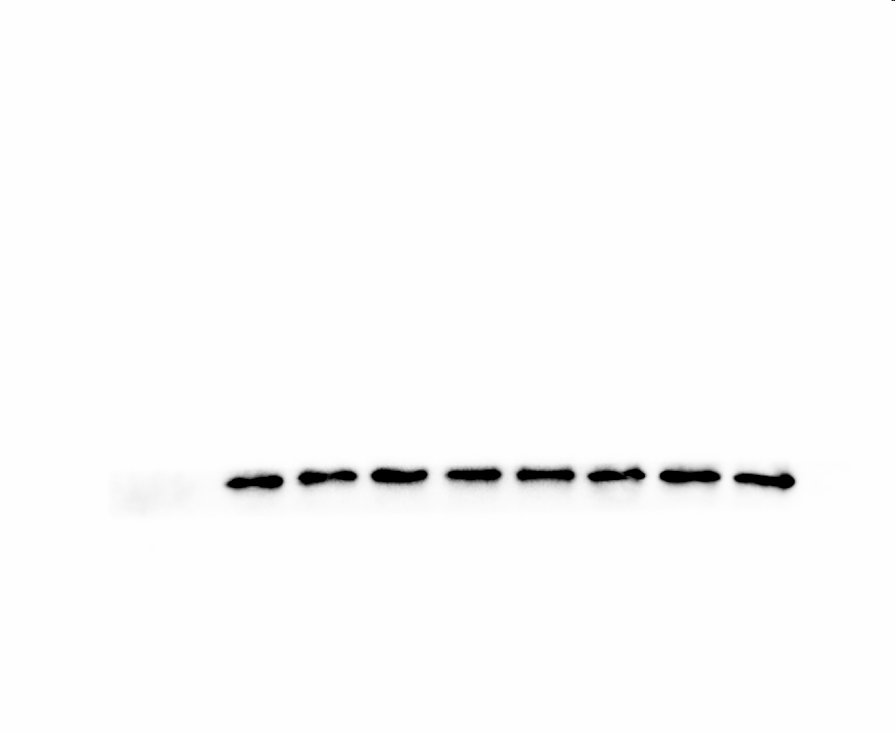

Supplement: Supplementary file 6 — western blots [file 41419_2023_6016_MOESM6_ESM.zip › breast cancer WB/Figure 6J/GADPH 1-2.jpg]

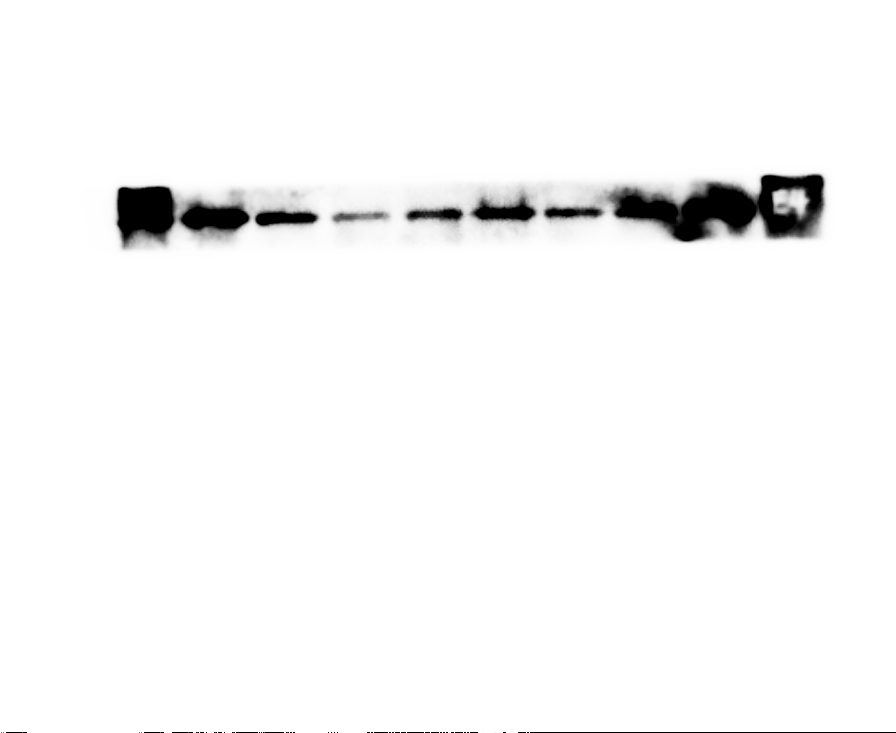

Supplement: Supplementary file 6 — western blots [file 41419_2023_6016_MOESM6_ESM.zip › breast cancer WB/Figure 6J/MMP-9 1-2.jpg]

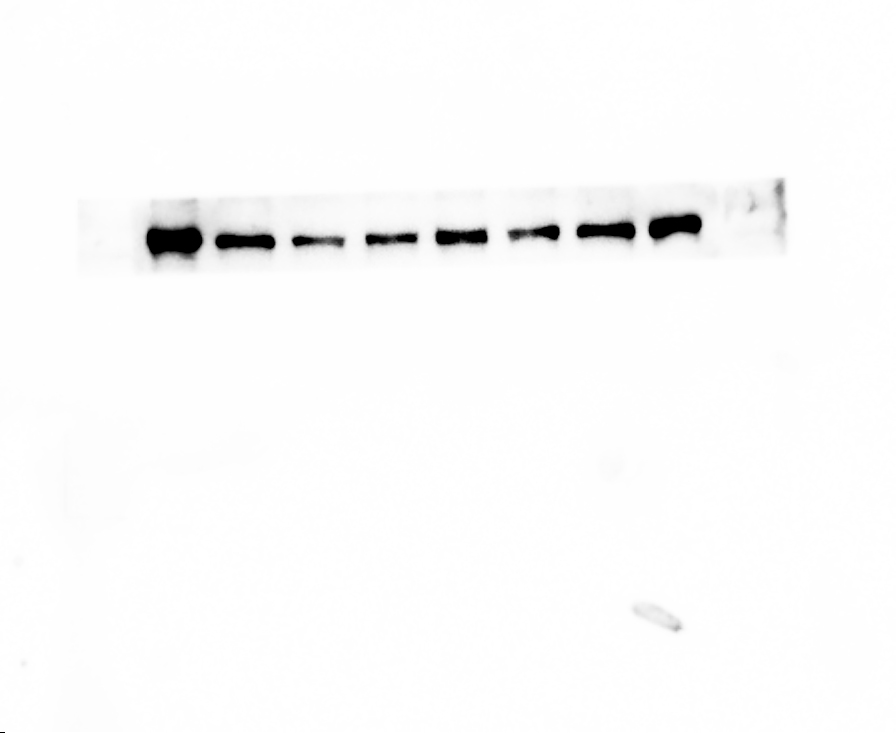

Supplement: Supplementary file 6 — western blots [file 41419_2023_6016_MOESM6_ESM.zip › breast cancer WB/Figure 6J/p-AKT 1-2.jpg]
